# Supplementary material for: Analysis of the determinants for using health research evidence in health planning in Tanzania: a cross-sectional study
Source: PLoS One. 2025 May 28;20(5):e0316508. doi: 10.1371/journal.pone.0316508 (PMC12118992; doi:10.1371/journal.pone.0316508)
Supplement: S1 Appendix — https://doi.org/10.7717/peerj.3077/supp-1. (DOCX) [file pone.0316508.s001.docx]

**S1 Dataset. (SAV)**

Table 1: Social demographic characteristics of respondents

| Variable | Frequency | Percent | Mean ±SD |
| --- | --- | --- | --- |
| **Age** |  |  | 39.51 ±7.86 |
| <25 | 3 | 0.71 |  |
| 26-35 | 159 | 37.68 |  |
| 36-45 | 156 | 36.97 |  |
| 46-55 | 93 | 22.04 |  |
| >46 | 11 | 2.61 |  |
| **Region** |  |  |  |
| Mbeya | 48 | 11.37 |  |
| Dodoma | 50 | 11.85 |  |
| Iringa | 41 | 9.72 |  |
| Mtwara | 49 | 11.61 |  |
| Mwanza | 50 | 11.85 |  |
| Kigoma | 51 | 12.09 |  |
| Kilimanjaro | 45 | 10.66 |  |
| Morogoro | 48 | 11.37 |  |
| Dar es salaam | 40 | 9.48 |  |
| **Type of health facility** |  |  |  |
| Dispensary | 37 | 8.77 |  |
| Health center | 82 | 19.43 |  |
| Council Hospital | 97 | 22.99 |  |
| Reginal referral hospital | 81 | 19.19 |  |
| Zonal hospital | 87 | 20.62 |  |
| CHMT | 38 | 9.00 |  |
| Gender |  |  |  |
| Male | 210 | 49.76 |  |
| Female | 212 | 50.24 |  |
| **Professional background** |  |  |  |
| Doctor | 107 | 25.36 |  |
| Nurse | 99 | 23.46 |  |
| Laboratory scientist | 36 | 8.53 |  |
| Pharmacist | 28 | 6.64 |  |
| Radiographer | 7 | 1.66 |  |
| Environmental Health officer | 13 | 3.08 |  |
| Nutrition officer | 13 | 3.08 |  |
| Social Welfare Officer | 20 | 4.74 |  |
| Physiotherapist | 1 | 0.24 |  |
| Biomedical engineer | 3 | 0.71 |  |
| Planning officer | 1 | 0.24 |  |
| Health secretary | 38 | 9.00 |  |
| Other | 56 | 13.27 |  |
| **Stakeholders' group** |  |  |  |
| RHMT | 49 | 11.61 |  |
| HMT | 280 | 66.35 |  |
| CHMT | 90 | 21.33 |  |
| FBO | 1 | 0.24 |  |
| HGFC | 2 | 0.47 |  |
| **Highest level of education** |  |  |  |
| Certificate | 30 | 7.11 |  |
| Diploma Certificate | 153 | 36.26 |  |
| Undergraduate degree | 180 | 42.65 |  |
| Master’s Degree | 56 | 13.27 |  |
| PhD | 3 | 0.71 |  |
| **Position holds currently in health planning** |  |  |  |
| Chairperson | 30 | 7.11 |  |
| Secretary | 45 | 10.66 |  |
| Technical advisor | 11 | 2.61 |  |
| Member | 335 | 79.38 |  |
| Other | 1 | 0.24 |  |
| **Years participate** |  |  | 5.08 ±3.96 |
| <5 | 283 | 67.06 |  |
| 6-10 | 103 | 24.41 |  |
| >10 | 36 | 8.53 |  |

Table 2: The use of health research evidence

| Variable | Frequency | Percent |
| --- | --- | --- |
| **Ever used evidence in health planning** |  |  |
| No | 14 | 3.32 |
| Yes | 408 | 96.68 |
| **Extent of using research evidence** |  |  |
| Low | 41 | 10.05 |
| Medium | 165 | 40.44 |
| High | 202 | 49.51 |
| **Types of evidence used during health planning (Multiple response)** |  |  |
| Evidence from routine Health information management data | 403 | 98.77 |
| Policy documents | 280 | 68.63 |
| Research publications (general) | 133 | 32.60 |
| Systematic reviews/ meta-analysis | 41 | 10.05 |
| Randomized Control trials | 17 | 4.17 |
| Experimental studies | 30 | 7.35 |
| Non-experimental studies | 20 | 4.90 |
| Expert opinions | 271 | 66.42 |
| Policy beliefs | 162 | 39.71 |
| Other | 17 | 4.17 |
| **Use of research evidence during health planning** |  |  |
| No | 138 | 33.82 |
| Yes | 270 | 66.18 |
| **Use of the Health planning guidelines during health planning** |  |  |
| No | 5 | 1.23 |
| Yes | 403 | 98.77 |
| **Use of the Ruling party manifesto during health planning** |  |  |
| No | 44 | 10.78 |
| Yes | 364 | 89.22 |
| **Use of the Policy documents during health planning** |  |  |
| No | 137 | 33.58 |
| Yes | 271 | 66.42 |
| **Is there an importance of the use of health research evidence during health plan** |  |  |
| No | 5 | 1.23 |
| Yes | 403 | 98.77 |
| **Level of importance of the use of health research evidence** |  |  |
| Very unimportant | 5 | 1.23 |
| Unimportant | 2 | 0.49 |
| Neutral | 14 | 3.43 |
| Important | 50 | 12.25 |
| Very important | 337 | 82.60 |

Table 3: Binary logistic regression for factors associated with using research evidence during health planning.

| Variable | Not use | Use | Unadjusted logistic analysis | | Adjusted logistic analysis | |
| --- | --- | --- | --- | --- | --- | --- |
|  | N (%) | N (%) | OR [95%CI] | p-value | OR [95%CI] | p-value |
| **Age** |  |  |  |  |  |  |
| <35 | 52(32.1) | 110(67.9) | ref |  |  |  |
| 36-45 | 56(35.9) | 100(64.1) | 0.844[0.530,1.343] | 0.4747 |  |  |
| >45 | 35(33.7) | 69(66.4) | 0.932[0.552,1.573] | 0.7919 |  |  |
| **Region** |  |  |  |  |  |  |
| Kilimanjaro | 26(57.8) | 19(42.2) | ref |  | ref |  |
| Mbeya | 17(35.4) | 31(64.6) | 2.495[1.081,5.760] | 0.0322 | 2.604[1.074,6.315] | 0.0342 |
| Dodoma | 17(34.0) | 33(66.0) | 2.656[1.156,6.104] | 0.0214 | 2.514[1.053,6.001] | 0.0378 |
| Iringa | 10(24.4) | 31(75.6) | 4.242[1.680,10.71] | 0.0022 | 4.707[1.772,12.499] | 0.0019 |
| Mtwara | 21(42.9) | 28(57.1) | 1.825[0.805,4.137] | 0.1499 | 1.801[0.727,4.466] | 0.2039 |
| Mwanza | 12(24.0) | 38(76.0) | 4.333[1.801,10.43] | 0.0011 | 4.794[1.893,12.140] | 0.0009 |
| Kigoma | 10(19.6) | 41(80.4) | 5.611[2.259,13.94] | 0.0002 | 5.922[2.224,15.769] | 0.0004 |
| Morogoro | 17(35.4) | 31(64.6) | 2.495[1.081,5.760] | 0.0322 | 2.180[0.901,5.274] | 0.0839 |
| Dar es salaam | 13(32.5) | 27(67.5) | 2.842[1.170,6.904] | 0.0211 | 3.229[1.211,8.611] | 0.0192 |
| **Type of health facility** |  |  |  |  |  |  |
| Dispensary | 17(46.0) | 20(54.1) | ref |  | ref |  |
| Health center | 25(30.5) | 57(69.5) | 1.938[0.871,4.311] | 0.1048 | 2.029[0.829,4.964] | 0.1211 |
| Council Hospital | 45(46.4) | 52(53.6) | 0.982[0.459,2.100] | 0.9631 | 0.809[0.323,2.026] | 0.6511 |
| RRH | 19(23.5) | 62(76.5) | 2.774[1.214,6.335] | 0.0155 | 2.663[1.895,7.920] | 0.0383 |
| RHMT | 27(31.0) | 60(69.0) | 1.889[0.857,4.163] | 0.1147 | 0.350[0.031,3.902] | 0.3932 |
| CHMT | 10(26.3) | 28(73.7) | 2.380[0.903,6.273] | 0.0795 | 5.647[0.602,52.94] | 0.3167 |
| Gender |  |  |  |  |  |  |
| Male | 71(33.8) | 139(66.2) | 1.007[0.673,1.507] | 0.9736 |  |  |
| Female | 72(34.0) | 140(66.0) | ref |  |  |  |
| **Professional background** |  |  |  |  |  |  |
| Doctor | 37(34.6) | 70(65.4) | ref |  |  |  |
| Nurse | 35(35.4) | 64(64.7) | 0.967[0.545,1.714] | 0.9073 |  |  |
| Lab scientist &Pharmacist | 25(35.2) | 46(64.8) | 0.973[0.518,1.825] | 0.9310 |  |  |
| Non-medical | 28(31.5) | 61(68.5) | 1.152[0.633,2.096] | 0.6444 |  |  |
| Other | 18(32.1) | 38(67.7) | 1.116[0.561,2.220] | 0.7548 |  |  |
| **Stakeholders' group** |  |  |  |  |  |  |
| RHMT | 13(26.5) | 36(73.5) | 1.609[0.816,3.171] | 0.1697 | 0.843[0.177,4.012] | 0.8297 |
| CHMT | 26(28.9) | 64(71.1) | 1.430[0.854,2.396] | 0.1741 | 5.647[0.602,52.93] | 0.1295 |
| HMT | 104(36.8) | 179(63.3) | ref |  | ref |  |
| **Highest level of education** |  |  |  |  |  |  |
| Certificate | 15(50.0) | 15(50.0) | ref |  | ref |  |
| Diploma | 57(37.3) | 96(62.8) | 1.684[0.767,3.700] | 0.1943 | 1.609[0.670,3.867] | 0.2874 |
| Undergraduate | 53(29.4) | 127(70.6) | 2.396[1.094,5.249] | 0.0290 | 1.824[0.710,4.685] | 0.2120 |
| Master’s Degree | 18(30.5) | 41(69.5) | 2.278[0.921,5.631] | 0.0747 | 2.082[0.684,6.337] | 0.1968 |
| **Position holds currently in health planning** |  |  |  |  |  |  |
| Secretary | 19(42.2) | 26(57.8) | ref |  | ref |  |
| Chairperson | 8(26.7) | 22(73.3) | 2.009[0.738,5.475] | 0.1724 | 1.933[0.647,5.780] | 0.2381 |
| Technical | 4(36.4) | 7(63.6) | 1.279[0.327,5.000] | 0.7236 | 0.789[0.173,3.599] | 0.7600 |
| Member | 112(33.3) | 224(66.7) | 1.462[0.776,2.754] | 0.2404 | 1.328[0.651,2.708] | 0.4354 |
| **Years participate** |  |  |  |  |  |  |
| <5 | 99(35.0) | 184(65.0) | ref |  |  |  |
| 6-10 | 34(33.0) | 69(67.0) | 1.092[0.677,1.761] | 0.7184 |  |  |
| >10 | 10(27.8) | 26(72.2) | 1.399[0.648,3.019] | 0.3924 |  |  |

Table 4: **Among the following, what is the importance of health research evidence used during health planning**

| Variable | VUIM n (%) | UIM n (%) | Neutral n (%) | IM n (%) | VIM n (%) |
| --- | --- | --- | --- | --- | --- |
| Gives the latest information | 5(1.23) | 3(1.23) | 32(7.84) | 100(24.51) | 268(65.69) |
| Help the policymakers understand a specific problem | 3(0.74) | 4(0.98) | 30(7.35) | 99(24.26) | 272(66.67) |
| Help the policymakers to implement various health interventions | 3(0.74) | 5(1.23) | 24(5.88) | 113(27.70) | 263(64.46) |
| Avoid repeating the failures of others | 3(0.74) | 3(0.74) | 19(4.66) | 101(24.75) | 282(69.12) |
| Introduces health planners to new ideas | 3(0.74) | 2(0.49) | 19(4.66) | 107(26.23) | 277(67.89) |

Table **5: Obstacles that hinder the utilization of health research evidence**

| Variable | SDSA n (%) | DSA n (%) | Neutral n (%) | AG n (%) | SAGR n (%) |
| --- | --- | --- | --- | --- | --- |
| Lack of necessary knowledge and training in research | 12(2.94) | 18(4.41) | 7(1.72) | 67(16.42) | 304(74.51) |
| Inadequate human and non-human resources | 16(3.92) | 18(4.41) | 7(1.72) | 81(19.85) | 286(70.10) |
| Difficult in accessing health research information | 36(8.82) | 41(10.05) | 24(5.88) | 110(26.96) | 197(48.28) |
| Perceptions that health research is for academic purposes | 92(22.55) | 40(9.80) | 16(3.92) | 81(19.85) | 179(43.87) |
| Lack of involvement in research activities | 15(3.68) | 25(6.13) | 11(2.70) | 93(22.79) | 264(64.71) |
| Traditional ways of planning, and Minimal usage of health research evidence among planning team members at the regional and council levels | 36(8.82) | 21(5.15) | 13(3.19) | 92(22.55) | 246(60.29) |
| lack of dissemination | 11(2.70) | 15(3.68) | 17(4.17) | 105(25.74) | 260(63.73) |

Table 6: Opportunities of the use of health research evidence

| Variable | SDSA n (%) | DSA n (%) | Neutral n (%) | AG n (%) | SAGR n (%) |
| --- | --- | --- | --- | --- | --- |
| **Physical opportunities** |  |  |  |  |  |
| Enabling utilization of information generated from the healthcare system at the | 6(1.42) | 9(2.13) | 18(4.27) | 130(30.81) | 259(61.37) |
| Availability of research coordinators | 9(2.13) | 18(4.27) | 22(5.21) | 93(22.04) | 280(66.35) |
| Availability of equipment | 4(0.95) | 16(3.79) | 21(4.98) | 88(20.85) | 293(69.43) |
| Availability of internet | 3(0.71) | 9(2.13) | 19(4.50) | 69(16.35) | 322(76.30) |
| Availability of planned budget for research | 11(2.61) | 17(4.03) | 27(6.40) | 64(15.17) | 303(71.80) |
| Presence of symposiums for research discussions | 9(2.13) | 17(4.03) | 23(5.45) | 106(25.12) | 267(63.27) |
| The existence of universities that provide a chance for dissemination | 8(1.90) | 17(4.03) | 22(5.21) | 103(24.41) | 272(64.45) |
| **Social opportunities** |  |  |  |  |  |
| The presence of disparities among different populations allows for more equitable | 10(2.37) | 24(5.69) | 38(9.00) | 147(34.83) | 203(48.10) |
| The presence of community members in the health planning teams | 11(2.61) | 28(6.64) | 55(13.03) | 119(28.20) | 209(49.53) |
| Improved health literacy in the society, empowering individuals to make informed | 4(0.95) | 19(4.50) | 18(4.27) | 145(34.36) | 236(55.92) |

Table 7: Motivations of the use of health research evidence

| Variable | SDSA n (%) | DSA n (%) | Neutral n (%) | AG n (%) | SAGR n (%) |
| --- | --- | --- | --- | --- | --- |
| **Automatic motivations** |  |  |  |  |  |
| Provision of incentives to health planners | 19(4.50) | 21(4.98) | 13(3.08) | 65(15.40) | 304(72.04) |
| Availability of job training | 3(0.71) | 4(0.95) | 16(3.79) | 90(21.33) | 309(73.22) |
| Availability of Short-term courses | 5(1.18) | 13(3.08) | 21(4.98) | 75(17.77) | 308(72.99) |
| Availability of Long-term course | 18(4.27) | 24(5.69) | 24(5.69) | 105(24.88) | 251(59.48) |
| **Reflective motivations** |  |  |  |  |  |
| Presence of interaction between policymakers, implementers, researchers, and aca | 3(0.71) | 17(4.03) | 21(4.98) | 111(26.30) | 270(63.98) |
| Presence of active stakeholder engagement in health planning | 6(1.42) | 16(3.79) | 33(7.82) | 116(27.49) | 251(59.48) |
| Continuous quality improvement in the healthcare system | 1(0.24) | 10(2.37) | 12(2.84) | 131(31.04) | 268(63.51) |
| Availability of transparency and accountability mechanisms | 5(1.18) | 12(2.84) | 27(6.40) | 98(23.22) | 280(66.35) |

Table 8: Readiness to use knowledge translation tools among planning teams

| Variable | SDSA n (%) | DSA n (%) | Neutral n (%) | AG n (%) | SAGR n (%) |
| --- | --- | --- | --- | --- | --- |
| I know what KT tools is | 56(13.27) | 28(6.64) | 24(5.69) | 161(38.15) | 153(36.26) |
| I know how to use KT tools | 52(12.32) | 31(7.35) | 25(5.92) | 170(40.28) | 144(34.12) |
| My institution provides opportunities for professional development | 86(20.38) | 25(5.92) | 29(6.87) | 120(28.44) | 162(38.39) |
| My institution cares about improving health services for communities through | 24(5.69) | 18(4.27) | 24(5.69) | 103(24.41) | 253(59.95) |
| I believe it is important to translate research findings for policymakers. | 8(1.90) | 9(2.13) | 16(3.79) | 75(17.77) | 314(74.41) |
| My institutions have the budget for KT tools | 156(36.97) | 40(9.48) | 55(13.03) | 74(17.54) | 97(22.99) |
| Using knowledge translation tools is an important consideration for my job promotion | 126(29.86) | 43(10.19) | 56(13.27) | 95(22.51) | 102(24.17) |
| I received training on how to use knowledge translation tools | 168(39.81) | 70(16.59) | 37(8.77) | 86(20.38) | 61(14.45) |
| The use of KT tools is included in the health planning guidelines | 108(25.59) | 35(8.29) | 59(13.98) | 121(28.67) | 99(23.46) |
| Donors support the use of KT tools because they want to have a real impact | 25(5.92) | 28(6.64) | 61(14.45) | 146(34.60) | 162(38.39) |
| Policymakers prefer the use of policy briefs than any other KT tools | 18(4.27) | 33(7.82) | 67(15.88) | 146(34.60) | 158(37.44) |


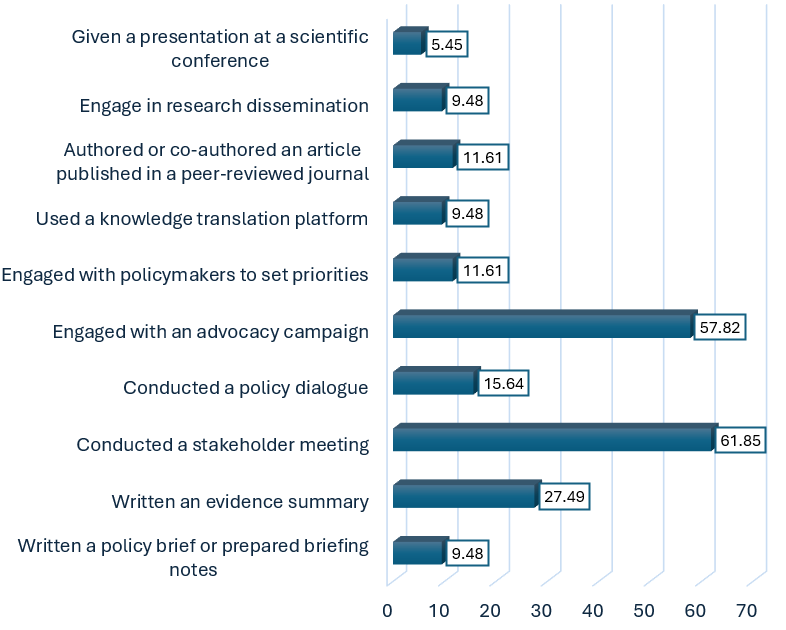


Figure 1: Activities in relation to Knowledge translation ever conducted by respondents


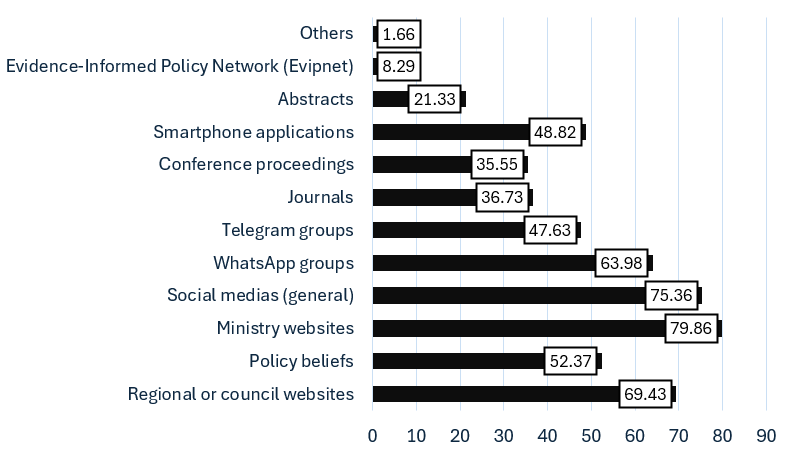


Figure 2: **knowledge translation tools available for use during health planning**

Table 9: Aspects that knowledge translation tools help during the planning

| Variable | SDSA n (%) | DSA n (%) | Neutral n (%) | AG n (%) | SAGR n (%) |
| --- | --- | --- | --- | --- | --- |
| Ensure accuracy of information | 18(4.27) | 45(10.66) | 52(12.32) | 100(23.70) | 207(49.05) |
| Identify priority issues | 20(4.74) | 34(8.06) | 42(9.95) | 135(31.99) | 191(45.26) |
| Save costs | 19(4.50) | 34(8.06) | 62(14.69) | 109(25.83) | 198(46.92) |
| Mitigate risks | 16(3.79) | 37(8.77) | 54(12.80) | 123(29.15) | 192(45.50) |
| Ensure compliance | 14(3.32) | 45(10.66) | 60(14.22) | 123(29.15) | 180(42.65) |

Table 10: **Readiness to use knowledge translation tools in health planning**

| Variable | SDSA n (%) | DSA n (%) | Neutral n (%) | AG n (%) | SAGR n (%) |
| --- | --- | --- | --- | --- | --- |
| **Knowledge translation tools usage** |  |  |  |  |  |
| The planning team know the planning software | 28(6.64) | 31(7.35) | 15(3.55) | 115(27.25) | 233(55.21) |
| The planning team know how to use the Internet during planning | 7(1.66) | 7(1.66) | 8(1.90) | 69(16.35) | 331(78.44) |
| The planning team capable of using smartphone applications | 7(1.66) | 4(0.95) | 17(4.03) | 91(21.56) | 303(71.80) |
| **Knowledge translation tools skills** |  |  |  |  |  |
| Ability to translate research into useful evidence | 33(7.82) | 37(8.77) | 24(5.69) | 180(42.65) | 148(35.07) |
| Can the planning team perform various tasks on planning software | 23(5.45) | 26(6.16) | 31(7.35) | 149(35.31) | 193(45.73) |
| **Knowledge translation tools literacy** |  |  |  |  |  |
| Heard about knowledge translation tools | 45(10.66) | 45(10.66) | 34(8.06) | 159(37.68) | 139(32.94) |
| Able to easily access the knowledge translation tools you need for health planning | 28(6.64) | 43(10.19) | 46(10.90) | 188(44.55) | 117(27.73) |
| Received support or training to find appropriate knowledge translation tools dur | 111(26.30) | 73(17.30) | 27(6.40) | 133(31.52) | 78(18.48) |
| Difficult to understand the content and purpose of the knowledge translation too | 78(18.48) | 76(18.01) | 66(15.64) | 150(35.55) | 52(12.32) |
| Received training on how to use knowledge translation tools, it was helpful | 140(33.18) | 75(17.77) | 46(10.90) | 103(24.41) | 58(13.74) |
| Confidently feel in the ability to use knowledge translation tools effectively | 44(10.43) | 70(16.59) | 56(13.27) | 171(40.52) | 81(19.19) |
| Effective tools are in translating health research evidence into practice | 20(4.74) | 31(7.35) | 65(15.40) | 148(35.07) | 158(37.44) |
| **Knowledge translation tools literacy** |  |  |  |  |  |
| I prefer acquiring new skills on KT tools through workshops, online courses | 1(0.24) | 10(2.37) | 3(0.71) | 61(14.45) | 347(82.23) |
| I am interested in formal training or more informal, peer-based learning environ | 1(0.24) | 5(1.18) | 13(3.08) | 76(18.01) | 327(77.49) |
| videos, articles, interactive modules) are most useful for your learning style | 2(0.47) | 9(2.13) | 7(1.66) | 111(26.30) | 293(69.43) |
| Encounter challenges when using KT tools during health planning | 28(6.64) | 16(3.79) | 36(8.53) | 150(35.55) | 192(45.50) |

**Appendix Questionnaire for Health Planning Team members.**

**Section A: Site Details**

1. Name of the Region: _____________________________________
2. Name of the District Council: ______________________________
3. Name of the Health Facility: _______________________________

**Section B: Respondent Demographic Information**

| 1. Gender: | |
| --- | --- |
| 1. Female | 1. Male |
| 1. What is your current age……. years | |
| 1. What is your professional background? | |
| 1. Doctor | 1. Nurse |
| 1. Laboratory scientist | 1. Pharmacist |
| 1. Radiographer | 1. Environmental Health officer |
| 1. Nutrition officer | 1. Social Welfare Officer |
| 1. Physiotherapist | 1. Biomedical engineer |
| 1. Planning officer | 1. Health secretary |
| 1. Others…specify |  |
| Which stakeholders’ group are you representing during health planning | |
| 1. RHMT | 1. HMT |
| 1. CHMT | 1. FBO |
| 1. HGFC | 1. Private sector |
| 1. NGO (e.g., implementing Partner) | 1. Others (specify) |
| 1. What is your highest level of education? | |
| 1. Certificate | 1. Diploma Certificate |
| 1. Undergraduate degree | 1. Master’s Degree |
| 1. Ph.D. | 1. Others (specify) |
| 1. What is your total number of years of schooling?........... | |
| 1. What position do you hold currently in health planning…………? 2. Chairperson 3. Secretary 4. Technical advisor 5. Member 6. Others…specify | |
| 1. How long have you been participating in planning activities? …………. (years) | |

**Section C: The use of health research evidence**

1. Have you ever used evidence in health planning?
2. Yes
3. No
4. To what extent have you used research evidence
5. Low
6. Medium
7. High
8. What types of evidence do you use during health planning (multiple responses)
9. Evidence from routine Health information management data
10. Policy documents
11. Research publications (general)
12. Systematic reviews/ meta-analysis
13. Randomized Control trials
14. Experimental studies
15. Non-experimental studies
16. Expert opinions
17. Policy beliefs
18. Others, specify
19. Do you use research evidence during health planning?, Yes
20. No
21. Do you use the following documents during health planning?

|  | **Response** | |
| --- | --- | --- |
| **Name of the Document** | Yes | No |
| Health planning guidelines |  |  |
| Ruling party manifesto |  |  |
| Policy documents |  |  |

**Section D: Capability of the use of health research evidence**

1. Is there an importance of the use of health research evidence during health planning?
   1. Yes
   2. No

14. What is the level of importance of the use of health research evidence

a. Very unimportant b. unimportant c. Neutral d. Important e. very important

1. Among the following, what is the importance of health research evidence used during health planning?

Use the scale of: 1 = Very unimportant, 2 = Unimportant, 3 = Neutral 4 = Important, 5 = Very important

| 1. Gives the latest information | **1** | **2** | **3** | **4** | **5** |
| --- | --- | --- | --- | --- | --- |
| 1. Help the policymakers understand a specific problem | **1** | **2** | **3** | **4** | **5** |
| 1. Help the policymakers to implement various health interventions |  |  |  |  |  |
| 1. Avoid repeating the failures of others | **1** | **2** | **3** | **4** | **5** |
| 1. Introduces health planners to new ideas. | **1** | **2** | **3** | **4** | **5** |

1. Rate the following factors that hinder the utilization of health research evidence

Use the scale of: 1 = strongly agree, 2 = somewhat agree, 3 = Neither agree nor disagree, 4 = somewhat disagree, 5 = strongly disagree

| Lack of necessary knowledge and training in research | **1** | **2** | **3** | **4** | **5** |
| --- | --- | --- | --- | --- | --- |
| Inadequate human and non-human resources | **1** | **2** | **3** | **4** | **5** |
| Difficult in accessing health research information |  |  |  |  |  |
| Perceptions that health research is for academic purposes | **1** | **2** | **3** | **4** | **5** |
| Lack of involvement in research activities | **1** | **2** | **3** | **4** | **5** |
| Traditional ways of planning, and Minimal usage of health research evidence among planning team members at the regional and council levels |  |  |  |  |  |
| lack of dissemination |  |  |  |  |  |

**Section E: Opportunities of the use of health research evidence**

16. Rate the following physical opportunities available for the use of health research evidence in health planning.

Use the scale of: 1 = strongly agree, 2 = somewhat agree, 3 = Neither agree nor disagree, 4 = somewhat disagree, 5 = strongly disagree

| Enabling utilization of information generated from the healthcare system at the Regional and council level | **1** | **2** | **3** | **4** | **5** |
| --- | --- | --- | --- | --- | --- |
| Availability of research coordinators | **1** | **2** | **3** | **4** | **5** |
| Availability of equipment |  |  |  |  |  |
| Availability of internet  Availability of planned budget for research | **1** | **2** | **3** | **4** | **5** |
| Presence of symposiums for research discussions | **1** | **2** | **3** | **4** | **5** |
| The existence of universities that provide a chance for dissemination |  |  |  |  |  |

17. Rate the following social opportunities available for the use of health research evidence in health planning.

Use the scale of: 1 = strongly agree, 2 = somewhat agree, 3 = Neither agree nor disagree, 4 = somewhat disagree, 5 = strongly disagree

| The presence of disparities among different populations allows for more equitable distribution of resources and targeted interventions. | **1** | **2** | **3** | **4** | **5** |
| --- | --- | --- | --- | --- | --- |
| The presence of community members in the health planning teams | **1** | **2** | **3** | **4** | **5** |
| Improved health literacy in the society, empowers individuals to make informed health decisions | **1** | **2** | **3** | **4** | **5** |

**Section F: Motivations of the use of health research evidence**

18. Rate the following automatic motivations available for the use of health research evidence in health planning.

Use the scale of 1 = strongly agree, 2 = somewhat agree, 3 = neither agree nor disagree, 4 = somewhat disagree, 5 = strongly disagree

| Provision of incentives to health planners | **1** | **2** | **3** | **4** | **5** |
| --- | --- | --- | --- | --- | --- |
| Availability of job training | **1** | **2** | **3** | **4** | **5** |
| Availability of Short term courses |  |  |  |  |  |
| Availability of Long-term course | **1** | **2** | **3** |  | **5** |

19. Rate the following reflective motivations available for the use of health research evidence in health planning.

Use the scale of: 1 = strongly agree, 2 = somewhat agree, 3 = neither agree nor disagree, 4 = somewhat disagree, 5 = strongly disagree

| Presence of interaction between policymakers, implementers, researchers, and academic institutions | **1** | **2** | **3** | **4** | **5** |
| --- | --- | --- | --- | --- | --- |
| Presence of active stakeholder engagement in health planning | **1** | **2** | **3** | **4** | **5** |
| Continuous quality improvement in the healthcare system | **1** | **2** | **3** | **4** | **5** |
| Availability of transparency and accountability mechanisms | **1** | **2** | **3** | **4** | **5** |

**Section G: Readiness to use knowledge translation tools among planning team**

Please identify the extent to which you agree or disagree with each of the following statements Use the scale of 1 = strongly agree, 2 = somewhat agree, 3 = Neither agree nor disagree, 4 = somewhat disagree, 5 = strongly disagree

I know what is KT tools…………………………………………………………1 2 3 4 5

I know how to use KT tools…………………………………………………….1 2 3 4 5

| My institution provides opportunities for professional development for the use of KT tools. | 1 | 2 | 3 | 4 | 5 |
| --- | --- | --- | --- | --- | --- |
| My institution cares about improving health services for communities through the use of KT tools | 1 | 2 | 3 | 4 | 5 |
| I believe it is important to translate research findings for policymakers. | 1 | 2 | 3 | 4 | 5 |
| My institutions have the budget for KT tools | 1 | 2 | 3 | 4 | 5 |
| Using knowledge translation tools is an important consideration for my job promotion. | 1 | 2 | 3 | 4 | 5 |
| I received training on how to use knowledge translation tools. | 1 | 2 | 3 | 4 | 5 |
| The use of KT tools is included in the health planning guidelines | 1 | 2 | 3 | 4 | 5 |
| Donors support the use of KT tools because they want to have a real impact on health service delivery | 1 | 2 | 3 | 4 | 5 |
| Policymakers prefer the use of policy briefs than any other KT tools |  |  |  |  |  |

Have you conducted any of the following activities in relation to Knowledge translation during health planning? (Select all that apply)

Written a policy brief or prepared briefing notes written an evidence summary

Conducted a stakeholder meeting

Conducted a policy dialogue

Engaged with an advocacy campaign

Engaged with policymakers to set priorities

Used a knowledge translation platform

Authored or co-authored an article published in a peer-reviewed journal

Conducted a systematic and/or rapid review

Engage in research dissemination

Given a presentation at a scientific conference

24. Which of the following knowledge translation tools available for use during health planning.........................................?

1. Regional or council websites
2. Policy beliefs
3. Ministry websites
4. Social medias (general)
5. WhatsApp groups
6. Telegram groups
7. Journals
8. Conference proceedings
9. Smartphone applications
10. Abstracts
11. Plan rep
12. Evidence-Informed Policy Network (Evipnet)
13. Others…specify

25. How do the above-mentioned tools in question 5 help during the planning?

1. Ensure accuracy of information 1. Yes 2.No
2. Identify priority issues. 1. Yes 2. No
3. Save costs 1. Yes, 2. No
4. Mitigate risks 1. Yes, 2. No
5. Ensure compliance 1. Yes, 2. No

26. Rate the following domains of readiness to use knowledge translation tools in health planning. Select according to what extent you agree or disagree with the domains. Use the scale 1 = strongly agree, 2 = somewhat agree, 3 = Neither agree nor disagree, 4 = somewhat disagree, 5 = strongly disagree

| 1. **Knowledge translation tools usage** |  |  |  |  |  |
| --- | --- | --- | --- | --- | --- |
| Does the planning team know the planning software | **1** | **2** | **3** | **4** | **5** |
| Does the planning team know how to use the Internet during planning | **1** | **2** | **3** | **4** | **5** |
| Are the planning team capable of using smart phone applications | **1** | **2** | **3** | **4** | **5** |
| 1. **Knowledge translation tools skills** |  |  |  |  |  |
| Ability to translate research into useful evidence | **1** | **2** | **3** | **4** | **5** |
| Ability to make research interpretation | **1** | **2** | **3** | **4** | **5** |
| Can the planning team perform various tasks on planning software | **1** | **2** | **3** | **4** | **5** |
| 1. **Knowledge translation tools literacy** |  |  |  |  |  |
| heard about knowledge translation tools | **1** | **2** | **3** | **4** | **5** |
| Able to easily access the knowledge translation tools you need for health planning |  |  |  |  |  |
| Received support or training to find appropriate knowledge translation tools during health planning |  |  |  |  |  |
| Difficult to understand the content and purpose of the knowledge translation tools you use during health planning |  |  |  |  |  |
| Received training on how to use knowledge translation tools, it was helpful |  |  |  |  |  |
| Confidently feel in the ability to use knowledge translation tools effectively |  |  |  |  |  |
| Effective tools are in translating health research evidence into practice |  |  |  |  |  |
| 1. **Knowledge translation tools literacy** |  |  |  |  |  |
| I prefer acquiring new skills on KT tools through workshops, online courses, peer mentoring, hands-on practice | **1** | **2** | **3** | **4** | **5** |
| I am interested in formal training or more informal, peer-based learning environments regarding KT tools during health planning |  |  |  |  |  |
| videos, articles, interactive modules) are most useful for your learning style of KT tools |  |  |  |  |  |
| Encounter challenges when using KT tools during health planning |  |  |  |  |  |

**27. Document review checklist**

| Name of the Region |  |
| --- | --- |
| Name of the District |  |
| Name of the Facility |  |
| Name of the assessor |  |
| Date |  |
| Starting time |  |
| End time |  |
|  |  |

| S/N | **looking for evidence that planning teams use research evidence in their plans** | Data source | Are the documents available? | | | NOTES  Where applicable, check which guidelines emphasize on use of evidence and jot down which type of evidence is emphasized (is research evidence part of it?) | |
| --- | --- | --- | --- | --- | --- | --- | --- |
|  |  |  | **Yes** | **No** | **NA** | |  |
| 1 | Do you have a current fiscal year health plan? Review the presence of the current financial year plan | Check the availability of a plan from a Plan rep or hardcopy | ☐ | ☐ | ☐ | |  |
| 2 | Do you have a planning guideline? | Check the availability of hard copy or soft copy of those guidelines | ☐ | ☐ | ☐ | |  |
|  | presence of CCHP |  |  |  |  | |  |
|  | Presence of HF Planning Guide |  |  |  |  | |  |
|  | Presence of RHMT guide |  |  |  |  | |  |
|  | Presence of CHOP |  |  |  |  | |  |
| 3 | Presence of situational analysis in the plan | Check if they used any type of situational analysis i.e.; root cause analysis, fish-borne analysis, SWOT analysis, PESTEL analysis,etc |  |  |  | |  |
| 4 | Presence of invitation letters to attend various scientific conferences | Check the meeting files | ☐ | ☐ | ☐ | |  |
| 5 | Do the health plans have in text citation | Check the available references i.e. hardcopy |  |  |  | |  |
| 6 | Is there a budget for research available |  |  |  |  | |  |
| 7 | Is there a budget allocated for research in their health plan?  Review the budget for research that is allocated | Check the itemized amount of the budget in the plan |  |  |  | |  |
| 8 | Is the research coordinator among the planning team members who prepared the plan? Review the list of planning team members | The cover page of the plan |  |  |  | |  |
