# Supplementary material for: Analysis of the determinants for using health research evidence in health planning in Tanzania: a cross-sectional study
Source: PLoS One. 2025 May 28;20(5):e0316508. doi: 10.1371/journal.pone.0316508 (PMC12118992; doi:10.1371/journal.pone.0316508)
Supplement: Attachment — Submitted filename: PONE-D-24-56677_R1_reviewer MANUSCRIPT WITH REVIEW COMMENTS.pdf [file pone.0316508.s004.pdf]

PLOS ONE

Analyzing the determinants for using health research evidence in health planning in Tanzania: a cross-sectional study.

--Manuscript Draft--

**Manuscript Number:**	PONE-D-24-56677R1
**Article Type:**	Research Article
**Full Title:**	Analyzing the determinants for using health research evidence in health planning in Tanzania: a cross-sectional study.
**Short Title:**	The use of health research evidence during health planning
**Corresponding Author:**	Pius Kagoma, MD, MPH<br>University of Dodoma College of Health and Allied Sciences: The University of Dodoma College of Health Sciences<br>DODOMA, TANZANIA, UNITED REPUBLIC OF
**Keywords:**	Determinants; Research evidence use; Health planning; Tanzania
**Abstract:**	Introduction: Achieving Universal Health Coverage (UHC) requires utilizing research evidence to inform the health planning process. However, little information is available on the determinants for using research evidence in planning in Lower Middle-Income Countries (LMICs), including Tanzania. This study aims to investigate the determinants of using health research evidence in health planning in Tanzania.<br><br>Materials and methods: This quantitative study employed a cross-sectional design. Data on health research evidence and the factors influencing its use were collected using a structured questionnaire from 422 respondents within 9 regions of Tanzania from October to December 2023. The data were analyzed using STATA version 18 for descriptive and inferential statistics. The association between categorical variables was assessed using a chi-square test, while regression analysis was conducted to identify determinants, both at a 95% confidence level. Results: The study revealed that 270 (66.2%) of the health planning team members strongly agreed to use health research evidence during planning. However, significant barriers were identified, including lack of dissemination (74.5%), inadequate human and non-human resources (70.0%), and insufficient knowledge and training in research (63.7%). A chi-square test confirmed significant associations between these barriers and the reduced use of research evidence ($p<0.05$). Conversely, more than 70% of respondents identified opportunities such as the availability of research coordinators, university partnerships, available research budgets, and internet access, all significantly associated with increased health research evidence use. More than 50% of participants reported motivational factors like continuous quality improvement agenda in the healthcare sector, availability of short and long-term courses, on-the-job training, and provision of incentives like extra duty allowances were positively linked to research utilization.Conclusion: The study found that 66.2% of health planning team members used health research evidence in planning, but barriers like lack of dissemination, resource shortages, and inadequate training persisted. Interventions should focus on improving dissemination, resources, and training. Future research should explore strategies for enhancing these interventions.
**Order of Authors:**	Pius Kagoma, MD, MPH
	Richard Mongi, PhD
	Albino Kalolo, MD, PhD
**Response to Reviewers:**	P.O Box 1923,<br>Dodoma.<br>14th March 2025.<br>Editor-In-Chief,<br>PLOS ONE<br><br>RE: Submission of Revisions of the Manuscript for Publication<br>I would like to submit the revisions of the manuscript titled “Analyzing the Determinants

Powered by Editorial Manager® and ProduXion Manager® from Aries Systems Corporation

	for Using Health Research Evidence in Health Planning in Tanzania: A Cross-sectional Study". This manuscript presents the findings of a quantitative study aimed at understanding the factors influencing the use of health research evidence among health planning teams in Tanzania.<br>The relevance of this research lies in its potential to inform policy and practice interventions aimed at enhancing the utilization of evidence-based practices in healthcare planning, particularly in low-resource settings including Tanzania.<br>This manuscript is a research article and a new submission to PLOS ONE. The content of this manuscript has not been published or submitted elsewhere for consideration.<br><br>Sincerely,<br><br>Dr. Pius Kagoma<br>Corresponding Author
**Additional Information:**	
**Question**	**Response**
**Financial Disclosure**<br><br>Enter a financial disclosure statement that describes the sources of funding for the work included in this submission. Review the submission guidelines for detailed requirements. View published research articles from *PLOS ONE* for specific examples.<br><br>This statement is required for submission and **will appear in the published article** if the submission is accepted. Please make sure it is accurate.<br><br>**Funded studies**<br>Enter a statement with the following details:<br>• Initials of the authors who received each award<br>• Grant numbers awarded to each author<br>• The full name of each funder<br>• URL of each funder website<br>• Did the sponsors or funders play any role in the study design, data collection and analysis, decision to publish, or preparation of the manuscript?<br><br>Did you receive funding for this work?	The author(s) received no specific funding for this work.
**Competing Interests**<br><br>Use the instructions below to enter a competing interest statement for this submission. On behalf of all authors, disclose any competing interests that	The authors have declared that no competing interests exist.

<table>
<tr><td>could be perceived to bias this work—acknowledging all financial support and any other relevant financial or non-financial competing interests.

This statement is required for submission and will appear in the published article if the submission is accepted. Please make sure it is accurate and that any funding sources listed in your Funding Information later in the submission form are also declared in your Financial Disclosure statement.

View published research articles from PLOS ONE for specific examples.

NO authors have competing interests

Enter: The authors have declared that no competing interests exist.

Authors with competing interests

Enter competing interest details beginning with this statement:

I have read the journal's policy and the authors of this manuscript have the following competing interests: [insert competing interests here]

* typeset</td><td></td></tr>
<tr><td>Ethics Statement

Enter an ethics statement for this submission. This statement is required if the study involved:

• Human participants
• Human specimens or tissue
• Vertebrate animals or cephalopods
• Vertebrate embryos or tissues
• Field research

Write "N/A" if the submission does not require an ethics statement.</td><td>The study was granted ethical approval by the University of Dodoma Ethical Committee with Ref. No. MA.84/261/102/'A'/64/91. Permission to conduct the study and consent to participate in the study was sought from relevant authorities and participants, respectively. Participants received information about the purpose of the study and data protection.</td></tr>
</table>

Powered by Editorial Manager® and ProduXion Manager® from Aries Systems Corporation

General guidance is provided below. Consult the submission guidelines for detailed instructions. **Make sure that all information entered here is included in the Methods section of the manuscript.**<br><br>**Format for specific study types**<br><br>**Human Subject Research (involving human participants and/or tissue)**<br>• Give the name of the institutional review board or ethics committee that approved the study<br>• Include the approval number and/or a statement indicating approval of this research<br>• Indicate the form of consent obtained (written/oral) or the reason that consent was not obtained (e.g. the data were analyzed anonymously)<br><br>**Animal Research (involving vertebrate animals, embryos or tissues)**<br>• Provide the name of the Institutional Animal Care and Use Committee (IACUC) or other relevant ethics board that reviewed the study protocol, and indicate whether they approved this research or granted a formal waiver of ethical approval<br>• Include an approval number if one was obtained<br>• If the study involved *non-human primates*, add *additional details* about animal welfare and steps taken to ameliorate suffering<br>• If anesthesia, euthanasia, or any kind of animal sacrifice is part of the study, include briefly which substances and/or methods were applied<br><br>**Field Research**<br><br>Include the following details if this study involves the collection of plant, animal, or other materials from a natural setting:<br>• Field permit number<br>• Name of the institution or relevant body that granted permission	
**Data Availability**<br><br>Authors are required to make all data	Yes - all data are fully available without restriction

Powered by Editorial Manager® and ProduXion Manager® from Aries Systems Corporation

underlying the findings described fully available, without restriction, and from the time of publication. PLOS allows rare exceptions to address legal and ethical concerns. See the PLOS Data Policy and FAQ for detailed information.<br><br>A Data Availability Statement describing where the data can be found is required at submission. Your answers to this question constitute the Data Availability Statement and **will be published in the article**, if accepted.<br><br>**Important:** Stating 'data available on request from the author' is not sufficient. If your data are only available upon request, select 'No' for the first question and explain your exceptional situation in the text box.<br><br>Do the authors confirm that all data underlying the findings described in their manuscript are fully available without restriction?	
**Describe where the data may be found in full sentences. If you are copying our sample text, replace any instances of XXX with the appropriate details.**<br><br>• If the data are **held or will be held in a public repository**, include URLs, accession numbers or DOIs. If this information will only be available after acceptance, indicate this by ticking the box below. For example: *All XXX files are available from the XXX database (accession number(s) XXX, XXX.*).<br>• If the data are all contained **within the manuscript and/or Supporting Information files**, enter the following: *All relevant data are within the manuscript and its Supporting Information files.*<br>• If neither of these applies but you are able to provide **details of access elsewhere**, with or without limitations, please do so. For example:<br><br>*Data cannot be shared publicly because of [XXX]. Data are available from the*	All relevant data are within the manuscript and its Supporting Information files.

Powered by Editorial Manager® and ProduXion Manager® from Aries Systems Corporation

XXX Institutional Data Access / Ethics Committee (contact via XXX) for researchers who meet the criteria for access to confidential data.<br><br>*The data underlying the results presented in the study are available from (include the name of the third party and contact information or URL).*<br>• This text is appropriate if the data are owned by a third party and authors do not have permission to share the data.<br><br>* typeset	
Additional data availability information:	Tick here if your circumstances are not covered by the questions above and you need the journal's help to make your data available.

Manuscript Click here to access/download;Manuscript;Manuscript.docx

Analyzing the determinants for using health research evidence in health planning in Tanzania: a cross-sectional study.

Pius Kagoma*[1&2,] Richard Mongi[1], Albino Kalolo[3, 4]

[1]*Department of Public Health and Community Nursing, the University of Dodoma, Dodoma, Tanzania;*

[2]*Division of Health, Social Welfare and Nutrition Services, President's Office-Regional Administration Local Government, Dodoma, Tanzania;*

[3]*Department of Public Health, St. Francis University College of Health and Allied Sciences, Morogoro, Tanzania;*

[4]*Centre for Reforms, Innovation, Health Policies, and Implementation Research, Dodoma, Tanzania.*

*Corresponding author: Pius Kagoma; +255 (0) 76 5909942; P.O. Box 1923 Dodoma, Tanzania; piuskagoma@gmail.com

Richard Mongi; +255 (0) 67 6953510; P.O. Box 259, Dodoma, Tanzania; richiemongi@gmail.com

Albino Kalolo; +255 (0) 764340819; P.O. Box 175, Ifakara, Morogoro, Tanzania; kaloloa@gmail.com

Abstract

Introduction: Achieving Universal Health Coverage (UHC) requires utilizing research evidence to inform the health planning process. However, little information is available on the determinants for using research evidence in planning in Lower Middle-Income Countries (LMICs), including Tanzania. This study aims to investigate the determinants of using health research evidence in health planning in Tanzania.

Materials and methods: This quantitative study employed a cross-sectional design. Data on health research evidence and the factors influencing its use were collected using a structured questionnaire from 422 respondents within 9 regions of Tanzania from October to December

2023. ~~The data were analyzed using STATA version 18 for descriptive and inferential statistics.~~ The association between categorical variables was assessed using a chi-square test, while regression analysis was conducted to identify determinants, both at a 95% confidence level. **Results:** The study revealed that 270 (66.2%) of the health planning team members strongly agreed to use health research evidence during planning. However, significant barriers were identified, including lack of dissemination (74.5%), inadequate human and non-human resources (70.0%), and insufficient knowledge and training in research (63.7%). A chi-square test confirmed significant associations between these barriers and the reduced use of research evidence ($p<0.05$). Conversely, more than 70% of respondents identified opportunities such as the availability of research coordinators, university partnerships, available research budgets, and internet access, all significantly associated with increased health research evidence use. More than 50% of participants reported motivational factors like continuous quality improvement agenda in the healthcare sector, availability of short and long-term courses, on-the-job training, and provision of incentives like extra duty allowances were positively linked to research utilization.

Conclusion: The study found that 66.2% of health planning team members used health research evidence in planning, but barriers like lack of dissemination, resource shortages, and inadequate training persisted. Interventions should focus on improving dissemination, resources, and training. Future research should explore strategies for enhancing these interventions.

Keywords: Determinants, Research evidence use, Health planning, Tanzania.

INTRODUCTION

Health research evidence plays an essential role in shaping effective health policies and interventions, which are crucial for improving public health outcomes (1). In resource-limited settings like Tanzania, where health challenges are complex and resources are constrained, the strategic use of research evidence is particularly important. It can guide decision-makers in identifying priority areas, optimizing resource allocation, and implementing interventions that

are both cost-effective and impactful. Moreover, the use of evidence can help health planning teams avoid repeating past failures and introduce innovative solutions (2). Despite its recognized importance, the extent to which health research evidence is utilized by health planning teams in Tanzania remains unclear. Understanding the factors that influence this utilization is essential for promoting evidence-based decision-making, which is critical for achieving the ambitious health targets set forth in the Sustainable Development Goals (SDGs), particularly Universal Health Coverage (UHC)(3).

Efforts to promote the integration of research evidence into health planning and decision-making have been made in various settings. These include the establishment of knowledge translation platforms, research dissemination strategies, health conferences, and capacity-building initiatives targeting health planning teams. However, the success of these efforts varies significantly across contexts, largely depending on factors such as institutional support, the capacity of the health workforce, and access to reliable data. In Tanzania, while some initiatives aim to bridge the gap between research and practice, the actual impact of these efforts on health planning remains under-explored.

Research across different settings has demonstrated that the use of health research evidence in decision-making is influenced by a variety of factors. These include organizational culture, the availability of resources, the expertise of the planning teams, and access to research outputs [4]. In a study conducted in Tanzania (4), it was found that health planning teams in Tanzania consist of professionals from various disciplines, including Administrators (9%), Medical doctors (25.36%), Nurses (23.46%), Laboratory scientists (8.53%), Pharmacists (6.64%), Radiographers (1.66%), Environmental health officers (3.08%), Nutrition officers (3.08%), Social welfare officers (4.74%), and others (14.46%). Their primary responsibility is to plan, implement, and evaluate health interventions [4]. However, several factors may limit their ability to integrate research evidence into their work fully (5). These include inadequate dissemination of research findings, lack of access to research evidence, limited skills in interpreting research data, and insufficient organizational support (3,6–8).

While previous studies have explored various factors influencing the use of health research evidence in policy and planning, there remains a gap in understanding the specific determinants

that affect the utilization of health research evidence among health planning teams in Tanzania(4,9). This study seeks to fill this gap by analyzing the key factors that influence evidence use among these teams at both the regional and council levels, guided by the COM-B Model (Capability, Opportunity, and Motivation) as the theoretical framework (10), which has been widely used in understanding evidence-based decision-making processes. The COM-B model is particularly suited to the Tanzanian context because it focuses on the essential components of behavior change Capability, Opportunity, and Motivation which are critical for understanding and addressing the factors influencing the use of health research evidence by planning teams. By examining these elements, the model helps identify specific barriers and enablers that can be targeted to improve evidence-based decision-making in Tanzania's resource-limited. By applying this theoretical lens, the study aims to provide a deeper understanding of the barriers and facilitators to using research evidence in health planning, with the following specific objectives: 1) Analyze the current usage of health research evidence among planning team members at the regional and council levels, 2) Analyze the capacity of health planning team members to utilize health research evidence, 3) Identify opportunities for enhancing the use of health research evidence in health planning, 4) Identify opportunities for enhancing the use of health research evidence in health planning, 5) Explore the motivations for using health research evidence among health planning members at both regional and council levels. The findings of this study are expected to contribute to the development of strategies that enhance the integration of research evidence into health planning processes, ultimately leading to more effective health interventions and improved health outcomes in Tanzania.

Conceptual framework

The conceptual framework for this study was developed after several consultative meetings with different stakeholders and researchers. The theory was adopted and modified from the (Capability, Opportunity, and Motivation) COM-B model. The COM- B Model at the center of a proposed framework is a behavior system involving three essential conditions: Capability, Opportunity, and Motivation, what we term the ‘COM-B system(10,11). This study investigated how the capability, opportunity, and motivation determinants can influence the behavior of the use of health research evidence during health planning as shown in Figure 1

…………………………………………………………………

Insert figure 1

………………………………………………………………….

Figure 1: The domains of the COM-B Model modified from Michie et al,.2011(10)

Materials and Methods

Study setting

This study was conducted between October and December 2023 in the United Republic of Tanzania, a Lower and Middle low-income country located in East Africa, with a population of about 62 million people. The allocated budget for the country's Ministry of Health in 2023/2024 was estimated to be 443.6 million US dollars, with 1.2 million US dollars (0.27%) allocated for evidence production. The health research evidence users in Tanzania involve three important Ministries, namely; the Ministry of Health (MoH), The President's Office Regional Administration and Local Government (PO-RALG), and Health management. The health evidence producers in Tanzania are the National Institute for Medical Research (NIMR), the Tanzania Commission for Science and Technology (COSTECH), Public and Private Universities, Health related institutions or authorities, local and international non-governmental organizations (NGOs), and civil society organizations as shown in Table 1.

Table 1: A list of health research evidence producers and users in Tanzania

SN	Evidence users
1	Ministry of Health
2	President's Office Regional Administration and Local Government
3	Ministry of Education Science and Technology (MEST)

4	Prime Minister's Office
5	Regional Health Management Teams
6	Council Health Management Teams
7	Healthcare workers
8	Non-Governmental Organizations (NGOs)
9	Civil Society Organizations (CSOs)
10	Private Sector
**Evidence producers**	
1	National Institute for Medical Research (NIMR)
2	Tanzania Commission for Science and Technology (COSTECH)
3	Public Universities; The Muhimbili University of Health and Allied Sciences (MUHAS), University of Dar es Salaam (UDSM), University of Dodoma (UDOM)
4	Private Universities; Catholic University of Health and Allied Sciences (CUHAS), Kilimanjaro Christian Medical Centre (KCMC), Hubert Kairuki Memorial University (HKMU), St. John's University of Tanzania (SJUT), St. Augustine University of Tanzania (SAUT), Kampala International University (KIU), St. Francis University College of Health and Allied Sciences (SFUCHAS)
5	Kilimanjaro Clinical Research Institute (KCRI)
6	Ifakara Health Institute (IHI)
7	Non-Governmental Organizations (NGOs)
8	Civil society organizations (CSOs)
9	Private sector

The study sites

The study site was eighteen (18) Councils of the Local Government Authorities within the nine (9) regions out of 26 Regions of Tanzania Mainland from nine (9) geographical zones as summarized in Table 2. The nine zones are selected to seek the country's geographical representation. Together, these regions have a total population of 21,119,700, which represents 35.7% of the Tanzanian population. These regions are heterogeneous in population size, distribution of health facilities, human resources for health, and institutions carrying out health research activities. A similar approach has been used in major Tanzanian health studies(4,5,12);

This approach provided a comprehensive understanding of the status quo for the use of health research evidence in health planning, and heterogeneous study population for random sampling hence acting as a representative snapshot of all regions in Tanzania. The study was conducted in 63 randomly selected health facilities, nine (9) Regional Referral Hospitals (RRHs), eighteen (18) District Hospitals (DHs), Eighteen (18) Health Centres (HCs), and eighteen (18) Dispensaries. To ensure the inclusion of urban and rural health facilities, stratification was conducted followed by random sampling.

Table 2: Summary of study sites

ZONE	REGION	COUNCIL
		Siha District
Northern	Kilimanjaro	Moshi Municipal
		Chamwino District
Central zone	Dodoma	Dodoma City
		Kigamboni Municipal
Dar es Salaam	Dar es Salaam	Dar es Salaam City
		Morogoro Municipal
Eastern	Mororgoro	Gairo District
		Kigoma District
Western	Kigoma	Kigoma Municipal
		Mbeya City
Southwest highland	Mbeya	Kyela District
		Kwimba District
Lake	Mwanza	Mwanza City
		Mtwara Municipal
Southern	Mtwara	Nanyamba
	Iringa	Iringa Municipal
Southern Highland		Iringa District

Study population

This study involved members of health planning teams at the regional and council levels in Tanzania. Eligible participants were those holding health planning roles in healthcare facilities or administrative positions, with at least one year of experience in health planning. Individuals who

were not part of the health planning team or had less than one year of experience were excluded from the study.

The health planning landscape in Tanzania

The health planning process in Tanzania is conducted at two levels; At the Council level, the health facility prepares its plans as well and the Council Health Management Team (CHMT) prepares its plan, whereby plans from the facility and CHMT are later consolidated to form Comprehensive Council plan (CCHP). The preparation of health facility plans is guided by the Health facility planning guidelines and CCHP guidelines.(13). At the regional level, the Regional Health Management Teams (RHMTs) prepare their plans using the RHMT planning guide and the Regional Referral Hospitals (RRHs) plans using the Comprehensive Operational Plan Guide (CHOP)(14). When the plans are completed are sent to the Ministry of Health and President’s Office-Regional Administration and Local Government for final assessment before being sent to the Ministry of Finance for funding. At all levels, we are planning to use the guidelines and the routine data collected from each level.(14).

Study Design

The study employs a quantitative cross-sectional design focusing on nine regions of Tanzania. (15,16).

Sample size and Sampling procedures

The sample size involved the health planning team members from the RRHs, DHs, HCs, and Dispensaries randomly selected from the nine regions. The sample size was 422 calculated from the Cochrane formula (1977). To date, in Tanzania, there is no cited reference for the percentage of the use of health research evidence in health planning therefore the assumed proportion of 50% was used (17).

$$n = \frac{Z^2 * p\,(1 - p)}{d^2} \quad \text{(Cochran (1977)}$$

Where by

N-sample size

Z-confidence interval 95% 1.96, P-proportional from previous study d = margin of error, which is approximately 5% Thus,

$$\mathbf{n= \frac{(1.96)^2 \times 50(100 - 50)}{(5)^2}}$$

=**384** including 10% of non-response sample 384 plus 10%=38+384=422

This study employed a multistage sampling technique for the selection of the study units as summarized in Figure 2. The sampling stages are zones, regions, councils, and public primary health facilities. The first stage was a random selection of one region from each of the nine Zones of the country. In the second stage, in each selected region, councils were clustered into rural and urban, and then one rural and one urban council was selected from each region followed by a random selection of the health facilities, see Figure 3. The technique is convenient for studying large and diverse populations (5). The sampling stages were zones, regions, councils, and public primary health facilities.

…………………………………………………..

Insert figure 2

…………………………………………………

Measurement of variables

Dependent variable

The dependent variable of this study, the use of health research evidence was measured using a a set of four (4) questions adapted from a previous study(18). The questions were a mixture of binary and multiple-choice questions. Those questions were divided into four areas. The mean score was calculated from those sets of questions; the score had a value of 0 and 1. Those respondents who scored 1 were regarded to have used the health research evidence, while those

who scored 0 have not used the health research evidence. For multiple choice questions, the respondents were provided with a set of predefined options, allowing them to select the one that best represents their response, which will capture varying degrees or categories on the use of health research evidence.

Independent Variables

The independent variables in this study encompass numerous factors including demographic information such as sex, age, and education level; professional attributes such as position within the health facility and years of schooling; and contextual elements including the type of health facility, stakeholders group represented district and region. The study aimed to gain insights into the determinants that influence the use of health research evidence during health planning. The determinants had a combination of three constructs which are, capability determinants, opportunity determinants, and motivation determinants both derived from the COM-B Model.

Capability determinants were Assessed using 12 Likert-scale questions (19) (1 = strongly agree to 5 = strongly disagree) and one multiple-choice question. Mean scores and standard deviations were calculated, and chi-square tests were used for categorical responses.

Opportunity determinants were evaluated through 17 Likert-scale questions, with responses assigned numerical values (1 to 5). Mean or median scores were used to quantify overall tendencies, and multiple regression analysis was conducted to determine their influence on the dependent variable.

Motivation determinants were measured using 8 Likert-scale questions, analyzed similarly to opportunity determinants, with multiple regression analysis applied to assess relationships with the dependent variable.

Data collection procedures and tools

Quantitative data was collected face to face person by using the Swahili version of the survey after translation of the English version of the survey and document review checklist (see supplemental file) to planning team members at national, regional, council, and health facility levels. The questions from the tool were adopted and modified from previous studies (18,20). The survey collected information on the use of health research evidence and its determinants from the sampled participants using Open Data Kit software (ODK).

The document review checklist was used to guide the document review to see whether the available plans have any evidence of being prepared using health research evidence. The checklist contains a list of questions that will help the review of the plans made at the facility, council, regional, and national levels. The checklist is used to collect information on the available health plans if they are prepared by using health research evidence. Data was collected by a research assistant, who was trained for three days in data collection methods, tools, and ethics. They were selected according to their experience in the health system. Pilot testing of the tools was conducted at Dodoma RHMT and Chamwino District Council at Chamwino CHMT, Chamwino Council Hospital, Chamwino Health Centre, and Buigiri Dispensary to enhance the validity, reliability, and effectiveness of the tool in capturing the intended information.

Data analysis

Responses were reported on all survey items in all response categories and summarized using frequencies and percentages for categorical variables and means and standard deviation for non-categorical variables were computed. Given that the outcome variable had two categories (0=Negative, 1=Positive), A binary logistic regression model was used to assess factors associated with the perception of implementers on the prime vendor system. The model results are regression parameter estimates and odds ratios (OR). The data analysis was conducted using STATA version 18, and the significance of all statistical tests was established at a 5% significance level.

Data validity and reliability

To ensure validity and reliability, the validity of quantitative data collection tools was reviewed by independent subject matter experts from the University of Dodoma (UDOM). The reliability assessment of the questionnaire was done using the internal consistency test, with the alpha reliability coefficient being the statistic. (21,22). The range of the alpha coefficient, also known as Cronbach's alpha was 0.71 acceptable to this study. Moreover, exploratory factor analysis to establish the construct validity of the questionnaire and pilot study conducted at Chamwino DC before field data collection to ensure clarity of the data collection tool.

Ethics and Dissemination

The study was granted ethical approval by the University of Dodoma Ethical Committee with **Ref. No. MA.84/261/102/'A'/64/91.** Permission to conduct the study and consent to participate in the study was sought from relevant authorities and participants, respectively. Participants received information about the purpose of the study and data protection. Permission to conduct the study was also obtained from the Local Government authorities and the Ministry of Health. Written informed consent was obtained from all participants before their involvement in the study. Participants were informed of their right to withdraw from the study at any time, and their participation was entirely voluntary. Confidentiality was strictly maintained by ensuring the anonymity of all documents containing participants' information.

Results

The finding from this study is presented in three parts using tables, percentages, and charts, Part one shows the respondents' demographic characteristics and stakeholder group. Part two shows the proportion of the respondents who reported using Health research evidence in health planning and, part three shows the association between health research use in planning and the COM-B constructs (capability, Motivation, and opportunity).

Demographic characteristics

Table 1 describes the respondents' characteristics and the stakeholder group they represent. There was an almost equal number of female 50.2 % and male 49.8% respondents with a mean age of 39.51 ±7.86years old. Most of them (42.7%) had undergraduate education followed by a Diploma (36.3%) and less than one-third (14.0%) had a master's degree and above. Few (7.1%) respondents had certificate-level education. Professional background results revealed that the majority 107 (25.4%) of the respondents were medical doctors followed by nurses 99 (23.5%). Physiotherapists and panning offices were very few each accounting for 0. 24% of the respondents.

Table 3: Demographic characteristics of respondents

**Variable**	**Frequency**	**Percent**
**Age**		
<25	3	0.7

26-35	159	37.7
36-45	156	37.0
46-55	93	22.4
>46	11	2.6
**Region**		
Mbeya	48	11.4
Dodoma	50	11.9
Iringa	41	9.7
Mtwara	49	11.6
Mwanza	50	11.85
Kigoma	51	12.1
Kilimanjaro	45	10.7
Morogoro	48	11.4
Dar es salaam	40	9.5
**Type of health facility**		
Dispensary	37	8.8
Health center	82	19.4
Council Hospital	97	23.0
Regional referral hospital	81	19.2
RHMT	87	20.6
CHMT	38	9.0
Gender		
Male	210	49.76
Female	212	50.24
**Professional background**		
Doctor	107	25.36
Nurse	99	23.46
Laboratory scientist	36	8.53
Pharmacist	28	6.64
Radiographer	7	1.66
Environmental Health officer	13	3.08
Nutrition officer	13	3.08
Social Welfare Officer	20	4.74
Physiotherapist	1	0.24
Biomedical engineer	3	0.71
Planning officer	1	0.24
Health Secretary	38	9.00
Other	56	13.3
**Stakeholders' group**		
RHMT	49	11.6
HMT	280	66.4
CHMT	90	21.3
FBO	1	0.2
HGFC	2	0.5
**Highest level of education**		

Certificate	30	7.1
Diploma Certificate	153	36.3
Undergraduate degree	180	42.7
Master’s Degree	56	13.3
PhD	3	0.7
**The position holds currently in health planning.**		
Chairperson	30	7.1
Secretary	45	10.7
Technical Advisor	11	2.6
Member	335	79.4
Other	1	0.2
**Years participate**		
<5	283	67.1
6-10	103	24.4
>10	36	8.5

Proportion of the use of health research evidence

Table 4 provides data on the use of health research evidence in a particular context. It shows that the majority of participants (96.7%) have used routine data, with 49.5% reporting a high extent of use. Additionally, evidence from routine data was the most commonly used type of evidence during health planning, with 98.8% of participants reporting its use. Most participants (66.2%) have used health research evidence as shown in Figure 3, and a significant majority (98.8%) have used health planning guidelines. Moreover, the majority of participants (98.8%) also indicated the importance of the use of health research evidence, with 82.6% considering it very important. These findings suggest a strong reliance on routine data and a high recognition of the importance of health research evidence in the context studied.

Table 4: The proportion of the use of health research evidence during health planning

Variable	Frequency	Percent
**Ever used routine data**		
No	14	3.3
Yes	408	96.7
**Extent of use of routine data**		
Low	41	10.1
Medium	165	40.4
High	202	49.5
**Type of evidence used during health planning (Multiple response)**		
Evidence from routine data	403	98.8

Policy documents	280	68.6
Research publications (general)	133	32.6
Systematic reviews/ meta-analysis	41	10.1
Randomized Control trials	17	4.2
Experimental studies	30	7.4
Non-experimental studies	20	4.9
Expert opinions	271	66.4
Policy beliefs	162	39.7
Other	17	4.2
**Use of health research evidence**		
No	138	33.8
Yes	270	66.2
**Use of the Health planning guidelines**		
No	5	1.2
Yes	403	98.8
**Use of the Ruling party manifesto**		
No	44	10.8
Yes	364	89.2
**Use of the Policy documents**		
No	137	33.6
Yes	271	66.4
**Importance of the use of health research evidence**		
No	5	1.2
Yes	403	98.8
**Level of importance of the use of health research evidence**		
Very unimportant	5	1.2
Unimportant	2	0.5
Neutral	14	3.4
Important	50	12.3
Very important	337	82.6

……………………………………………………………………………………

Insert figure 3

……………………………………………………………………………………..

Figure 4: Proportion of the use of health research evidence.

The factors associated with the use of health research evidence during health planning.

The table presents the results of a binary logistic regression analysis examining factors associated with the use of research evidence during health planning. The analysis includes variables such as age, region, type of health facility, gender, professional background, stakeholder group, highest level of education, position held in health planning, and years of participation. The adjusted odds ratios (OR), confidence intervals (CI), and p-values are provided to indicate the strength and statistical significance of associations.

Significant Variables and Interpretation

Region: The analysis compares various regions to the reference category, Kilimanjaro. The odds ratios (OR) show how likely individuals from other regions are to use health research evidence in health planning compared to those from Kilimanjaro. For instance, if a region has an OR greater than 1, individuals from that region are more likely to use research evidence than those from Kilimanjaro. If the OR is less than 1, they are less likely. The p-values indicate whether these differences are statistically significant.

Level of Education: Compared to individuals with a diploma (reference category), those with higher education levels were more likely to use research evidence:

Undergraduate degree (OR = 2.396, CI: [1.094, 5.249], p-value = 0.0290): Those with an undergraduate degree are over twice as likely to use research evidence compared to diploma holders.

Master's degree (OR = 2.278, CI: [0.921, 5.631], p-value = 0.0747): Similar to undergraduates, master's degree holders are also more likely to use research evidence in health planning although were not significant.

These OR values reflect the increased likelihood of individuals with higher education levels using research evidence compared to the reference group, with p-values indicating the strength of this relationship.

Years of Participation:

6–10 years (OR = 1.092, CI: [0.677,1.761], p-value = 0.7184): Individuals with 6–10 years of participation in health planning show a higher likelihood of using research evidence compared to those with fewer years, though this was not statistically significant.

More than 10 years (OR = 1.399, CI: [0.648,3.019], p-value = 0.3924): Those with over 10 years of participation have a 40% higher likelihood of using research evidence compared to those with fewer years of experience, and this difference was also not statistically significant.

The adjusted odds ratios reflect the likelihood of each category of the variable influencing the use of research evidence, controlling for other variables in the model. Significance is determined by both the size of the OR and the p-value, with confidence intervals providing a range within which the true effect likely falls.

Table 5: Binary logistic regression for factors associated with the use of research evidence during health planning.

Variable	Not use	Use	Unadjusted logistic analysis		Adjusted logistic analysis	
	N (%)	N (%)	OR [95%CI]	p-value	OR [95%CI]	p-value
**Age**						
<35	52(32.1)	110(67.9)	ref			
36-45	56(35.9)	100(64.1)	0.844[0.530,1.343]	0.4747		
>45	35(33.7)	69(66.4)	0.932[0.552,1.573]	0.7919		
**Region**						
Kilimanjaro	26(57.8)	19(42.2)	ref		ref	
Mbeya	17(35.4)	31(64.6)	2.495[1.081,5.760]	0.0322	2.604[1.074,6.315]	0.0342
Dodoma	17(34.0)	33(66.0)	2.656[1.156,6.104]	0.0214	2.514[1.053,6.001]	0.0378
Iringa	10(24.4)	31(75.6)	4.242[1.680,10.71]	0.0022	4.707[1.772,12.499]	0.0019
Mtwara	21(42.9)	28(57.1)	1.825[0.805,4.137]	0.1499	1.801[0.727,4.466]	0.2039
Mwanza	12(24.0)	38(76.0)	4.333[1.801,10.43]	0.0011	4.794[1.893,12.140]	0.0009
Kigoma	10(19.6)	41(80.4)	5.611[2.259,13.94]	0.0002	5.922[2.224,15.769]	0.0004
Morogoro	17(35.4)	31(64.6)	2.495[1.081,5.760]	0.0322	2.180[0.901,5.274]	0.0839
Dar es salaam	13(32.5)	27(67.5)	2.842[1.170,6.904]	0.0211	3.229[1.211,8.611]	0.0192
**Type of health facility**						
Dispensary	17(46.0)	20(54.1)	ref		ref	
Health center	25(30.5)	57(69.5)	1.938[0.871,4.311]	0.1048	2.029[0.829,4.964]	0.1211
Council	45(46.4)	52(53.6)	0.982[0.459,2.100]	0.9631	0.809[0.323,2.026]	0.6511

Hospital						
RRH	19(23.5)	62(76.5)	2.774[1.214,6.335]	0.0155	2.663[1.895,7.920]	0.0383
RHMT	27(31.0)	60(69.0)	1.889[0.857,4.163]	0.1147	0.350[0.031,3.902]	0.3932
CHMT	10(26.3)	28(73.7)	2.380[0.903,6.273]	0.0795	5.647[0.602,52.94]	0.3167
Gender						
Male	71(33.8)	139(66.2)	1.007[0.673,1.507]	0.9736		
Female	72(34.0)	140(66.0)	ref			
**Professional background**						
Doctor	37(34.6)	70(65.4)	ref			
Nurse	35(35.4)	64(64.7)	0.967[0.545,1.714]	0.9073		
Lab scientist &Pharmacist	25(35.2)	46(64.8)	0.973[0.518,1.825]	0.9310		
Non-medical	28(31.5)	61(68.5)	1.152[0.633,2.096]	0.6444		
Other	18(32.1)	38(67.7)	1.116[0.561,2.220]	0.7548		
**Stakeholders' group**						
RHMT	13(26.5)	36(73.5)	1.609[0.816,3.171]	0.1697	0.843[0.177,4.012]	0.8297
CHMT	26(28.9)	64(71.1)	1.430[0.854,2.396]	0.1741	5.647[0.602,52.93]	0.1295
HMT	104(36.8)	179(63.3)	ref		ref	
**Highest level of education**						
Certificate	15(50.0)	15(50.0)	ref		ref	
Diploma	57(37.3)	96(62.8)	1.684[0.767,3.700]	0.1943	1.609[0.670,3.867]	0.2874
Undergraduate	53(29.4)	127(70.6)	2.396[1.094,5.249]	0.0290	1.824[0.710,4.685]	0.2120
Master’s Degree	18(30.5)	41(69.5)	2.278[0.921,5.631]	0.0747	2.082[0.684,6.337]	0.1968
**Position holds currently in health planning**						
Secretary	19(42.2)	26(57.8)	ref		ref	
Chairperson	8(26.7)	22(73.3)	2.009[0.738,5.475]	0.1724	1.933[0.647,5.780]	0.2381
Technical	4(36.4)	7(63.6)	1.279[0.327,5.000]	0.7236	0.789[0.173,3.599]	0.7600
Member	112(33.3)	224(66.7)	1.462[0.776,2.754]	0.2404	1.328[0.651,2.708]	0.4354
**Years participate**						
<5	99(35.0)	184(65.0)	ref			
6-10	34(33.0)	69(67.0)	1.092[0.677,1.761]	0.7184		
>10	10(27.8)	26(72.2)	1.399[0.648,3.019]	0.3924		

Capability: The knowledge and skills of health research evidence use

Table 6 presents the findings from a survey on the capacity to use health research evidence (knowledge and skills). Each row in the table represents a different variable related to the use of health research evidence, and the columns display the responses categorized into five levels of

importance: Very Unimportant (VUIM), Unimportant (UIM), Neutral, Important (IM), and Very Important (VIM).

From the data, it's clear that the majority of respondents perceive the use of health research evidence as very important or important across the various variables. In the variable "Gives the latest information," the majority of respondents rated it as Very Important (65.7%) or Important (24.5%). Similarly, in the variable "Help the policymakers understand a specific problem," a substantial proportion of respondents indicated that it is Very Important (66.7%) or Important (24.3%). Overall, the data suggests that there is a strong recognition of the significance of health research evidence in various aspects of policymaking and intervention implementation.

Table 6: Knowledge and skills of health research evidence use

Variable	VUIM n (%)	UIM n (%)	Neutral n (%)	IM n (%)	VIM n (%)
Gives the latest information	5(1.2)	3(1.2)	32(7.8)	100(24.51)	268(65.7)
Help the policymakers understand a specific problem	3(0.7)	4(1.0)	30(7.4)	99(24.3)	272(66.7)
Help the policymakers to implement various health interventions	3(0.7)	5(1.2)	24(5.9)	113(27.7)	263(64.5)
Avoid repeating the failures of others	3(0.7)	3(0.7)	19(4.7)	101(24.8)	282(69.1)
Introduces health planners to new ideas	3(0.7)	2(0.5)	19(4.7)	107(26.2)	277(67.9)

Table 7 provides information on the obstacles hindering the utilization of health research evidence. The variables include Lack of necessary knowledge and training in research, Inadequate human and non-human resources, Difficulty in accessing health research evidence, Perceptions that health research is for academic purposes, Lack of involvement in research activities, and Traditional ways of planning, as well as the minimal usage of health research evidence among planning team members at the regional and council levels, and lack of dissemination.

The data is presented in terms of the number and percentage of participants who strongly disagree (SDSA), disagree (DSA), are neutral, agree (AG), and strongly agree (SAGR) with each obstacle. This data provides insight into the specific challenges that impact the utilization of health research evidence within the context of the study. The highest percentage is for "Perceptions that health research is for academic purposes" at 22.6%, and the lowest percentage is for "Lack of necessary knowledge and training in research" at 2.9%.

Table **7: Obstacles that hinder the utilization of health research evidence**

Variable	SDSA n (%)	DSA n (%)	Neutral n (%)	AG n (%)	SAGR n (%)
Lack of necessary knowledge and training in research	12(2.94)	18(4.4)	7(1.7)	67(16.4)	304(74.5)
Inadequate human and non-human resources	16(3.92)	18(4.4)	7(1.7)	81(19.9)	286(70.1)
Difficult in accessing health research evidence	36(8.82)	41(10.1)	24(5.9)	110(27.0)	197(48.3)
Perceptions that health research is for academic purposes	92(22.55)	40(9.8)	16(3.9)	81(19.9)	179(43.9)
Lack of involvement in research activities	15(3.68)	25(6.1)	11(2.7)	93(22.8)	264(64.7)
Traditional ways of planning, and Minimal usage of health research evidence among planning team members at the regional and council levels	36(8.82)	21(5.2)	13(3.2)	92(22.6)	246(60.3)
lack of dissemination	11(2.70)	15(3.7)	17(4.2)	105(25.7)	260(63.7)

Opportunities for the use of health research evidence

Table 8 indicates the opportunities for the use of health research evidence, helping stakeholders to identify areas for improvement and investment to optimize the use of health research

evidence. The table presents the opportunities for utilizing health research evidence, categorized into physical and social opportunities.

For physical opportunities, the table highlights the availability of resources such as research coordinators, equipment, internet access, a planned budget for research, and the presence of symposiums for research discussions. The percentages indicate the distribution of responses, ranging from strongly disagree to strongly agree, reflecting the varying levels of agreement among the respondents regarding these physical opportunities. In the physical opportunities category, the highest percentage is 76.3%, representing the availability of the internet, while the lowest percentage is 0.7%, indicating the availability of Internet.

In the social opportunities section, the table emphasizes the presence of disparities among different populations, the involvement of community members in health planning teams, and the enhancement of health literacy in society. The percentages provide an insight into the attitudes and perceptions of the respondents toward these social opportunities. In the social opportunities category, the highest percentage is 55.9%, reflecting improved health literacy in society, and the lowest percentage is 2.4%, which represents the presence of disparities among different populations allowing for more equitable representation.

Table 8: Opportunities for the use of health research evidence

Variable	SDSA n (%)	DSA n (%)	Neutral n (%)	AG n (%)	SAGR n (%)
**Physical opportunities**					
Enabling the utilization of information generated from the healthcare system	6(1.4)	9(2.1)	18(4.3)	130(30.8)	259(61.4)
Availability of research coordinators	9(2.1)	18(4.3)	22(5.2)	93(22.0)	280(66.4)
Availability of equipment	4(1.0)	16(3.8)	21(5.0)	88(20.9)	293(69.4)
Availability of internet	3(0.7)	9(2.1)	19(4.5)	69(16.4)	322(76.3)
Availability of planned budget for research	11(2.6)	17(4.0)	27(6.4)	64(15.2)	303(71.8)
Presence of symposiums for research discussions	9(2.1)	17(4.0)	23(5.5)	106(25.1)	267(63.3)

**Social opportunities**					
The presence of disparities among different populations allows for more equitable	10(2.4)	24(5.7)	38(9.0)	147(34.8)	203(48.1)
The presence of community members in the health planning teams	11(2.6)	28(6.6)	55(13.0)	119(28.2)	209(49.5)
Improved health literacy in the society, empowering individuals to make informed	4(1.0)	19(4.5)	18(4.3)	145(34.4)	236(55.9)

Motivations for the use of health research evidence

Table 9, based on the data presented, we can observe that the automatic motivations for the use of health research evidence are primarily centered around the availability of transparency and accountability mechanisms 66.4% strongly agreeing, and 23.2% agreeing with the statement availability of job training 73.2% strongly agreeing and 21.3% agreeing respectively. Provision of incentives to health planners, with 72.4% strongly agreeing and 15.4% agreeing respectively.

In contrast, reflective motivations such as continuous quality improvement in the healthcare system and the presence of active stakeholder engagement in health planning also garnered significant support, with 63.51% and 59.48% strongly agreeing or agreeing, respectively. This indicates a strong emphasis on the proactive involvement of various stakeholders and the continuous enhancement of the healthcare system based on research evidence.

Table 9: Motivations of the use of health research evidence

Variable	SDSA n (%)	DSA n (%)	Neutral n (%)	AG n (%)	SAGR n (%)
**Automatic motivations**					
Provision of incentives to health planners	19(4.5)	21(5.0)	13(3.1)	65(15.4)	304(72.0)
Availability of job training	3(0.7)	4(1.0)	16(3.8)	90(21.3)	309(73.2)
Availability of Short-term courses	5(1.2)	13(3.1)	21(5.0)	75(17.8)	308(73.0)
Availability of Long-term course	18(4.3)	24(5.7)	24(5.7)	105(24.9)	251(59.5)

**Reflective motivations**					
Presence of interaction between policymakers, implementers, researchers, and academicians	3(0.7)	17(4.0)	21(5.0)	111(26.3)	270(64.0)
Presence of active stakeholder engagement in health planning	6(1.4)	16(3.8)	33(7.8)	116(27.5)	251(59.5)
Continuous quality improvement in the healthcare system	1(0.2)	10(2.4)	12(2.8)	131(31.0)	268(63.5)
Availability of transparency and accountability mechanisms	5(1.2)	12(2.8)	27(6.4)	98(23.2)	280(66.4)

Findings from the document review checklist

The analyzed data from Table 10 indicates that the majority of planning teams incorporate research evidence into their health plans. A substantial 97.8% of respondents confirmed having a current fiscal year health plan, and 98.9% reported having a planning guideline. Most plans include key health frameworks and guides, with 70.8% having the CCHP, 83.2% using the HF Planning Guide, and 89.9% incorporating situational analysis. Scientific engagement is evidenced by 75.3% of teams receiving invitation letters for conferences. However, challenges remain, as only 22.5% have a budget for research, and only 23.6% allocate a budget specifically for research within the health plan. Additionally, just 28.1% include a research coordinator in the planning team, and 43.8% of the plans are well-cited. This highlights a strong foundation in research usage but also gaps in financial and personnel support for research-driven planning.

Table 10: looking for evidence that planning teams use health research evidence in their plans

Variable	No	Yes	Not applicable
	n (%)	n (%)	n (%)
Do you have a current fiscal year health plan?	1(1.1)	87(97.8)	1(1.1)
Do you have a planning guideline?	1(1.12)	88(98.9)	0(0.0)
Presence of CCHP	12(13.5)	63(70.8)	14(15.7)
HF Planning Guide	13(14.6)	74(83.2)	2(2.3)
RHMT guide	26(29.2)	20(22.5)	43(48.3)

CHOP	30(33.7)	24(27.0)	35(39.3)
Presence of situational analysis in the plan	8(9.0)	80(89.9)	1(1.1)
Presence of invitation letters to attend various scientific conferences	22(24.7)	67(75.3)	0(0.0)
Are the well-cited plans	50(56.2)	39(43.8)	0(0.0)
Is there a budget for research available	69(77.5)	20(22.5)	0(0.0)
Is there a research-allocated budget in their health plan?	68(76.4)	21(23.6)	0(0.0)
Is the research coordinator among the planning team members who prepared the plan	64(71.9)	25(28.1)	0(0.0)

Discussion

This study represents the first comprehensive analysis of the use of health research evidence in Tanzania. It aimed to analyze the determinants of the use of health research evidence by deep-diving into the three determinants influencing behavior i.e. Capability, Opportunity, and Motivation. The findings from this study indicated that all three determinants significantly impact the ability of health planners to use health research evidence during health planning. However, we found that the use of research evidence is associated with having a degree, more than 10 years of involvement in health planning activities, and prior knowledge in research. We found several facilitator determinants such as organizational support infrastructures like, the availability of computers and the internet, the availability of research experts and research coordinators, availability of academic institutions that give a chance for dissemination of research. On the other hand, barrier determinants included lack of human resources, lack of funding for research activities, lack of necessary knowledge and training, perceptions that research is for academic purposes, and lack of dissemination.

The findings of this study on the proportion of the use of health research evidence demonstrate a high level of reliance on health research evidence, particularly routine data, during health planning. The majority of participants (96.7%) reported using routine data, with nearly half

(49.5%) using it extensively, which is consistent with findings from similar studies. For instance, a study by Lavis et al (23) found that routine data and health statistics are frequently utilized in policy-making and health-planning processes, underscoring the central role of such data in evidence-based decision-making. Moreover, the widespread use of health planning guidelines (98.8%) and the high proportion of participants (98.8%) recognizing the importance of health research evidence align with findings from studies on the use of research evidence in health decision-making process which emphasize the role of guidelines and evidence in shaping health policies (24,25). The high rate of health research evidence use (66.2%) in this study also mirrors the findings which reported similar levels of research evidence use in healthcare settings(26). These results highlight the growing recognition and integration of research evidence in health planning, reflecting broader trends in evidence-informed policymaking across health systems in Tanzania and globally.

Explicitly, capability determinants such as knowledge and skills were found to be critical, with health planning team members who had higher levels of education (Undergraduate, a master level, and above) and access to training more likely to use health research evidence to almost 70%, with the capability constraints faces more those with certificate and diploma level, lack of necessary knowledge and training in research accounts up to 70%, being the most barrier to utilization of health research evidence, inadequate human and non-human resources, difficult in accessing research evidence, perception that research is for academic purposes and lack of dissemination. These findings are consistent with systematic reviews conducted in other low- and middle-income countries, where similar capability constraints such as poor access to good quality relevant research, and lack of timely research output have been identified as major barriers to evidence-based health planning (27,28).

In comparing our results with existing literature on Opportunity determinants (both physical and social), it is evident that the availability of resources and institutional support plays a crucial role in enabling the use of research evidence. Our study revealed that health planning teams with better access to physical opportunities such as health information systems, availability of research coordinators, equipment, internet, research budgets, presence of symposiums for research discussion, the existence of academic institutions such as universities, and social opportunities like the presence of community members representatives within the health

planning teams and improved health literacy in the community were more inclined to utilize research findings in their work. This aligns with findings from two studies conducted in LMICs (29,30), which highlighted the importance of institutional support in facilitating the use of research evidence in health policy. However, our study also noted a distinct challenge in the Tanzanian context with limited access to relevant health research evidence due to infrastructural constraints, a barrier less emphasized in studies from more resource-rich settings (31,32).

Particularly the automatic motivations to utilize health research evidence were also found to be significant, especially on the provision of incentives to health planners to greater than 72%. Other motivations include job training, and short and long-term courses that influenced the motivations to use the health research evidence. The automatic motivation factors included the presence of interaction between policymakers, implementers, researchers, and academicians, and continuous quality improvement agenda in the health sector. Health planners who perceived the use of research evidence as beneficial and aligned with their professional goals were more likely to engage with it actively. This is in line with the COM-B model, which underscores the role of motivation in behavior change (10). Our findings add to the body of literature by highlighting the specific motivational barriers in Tanzania, such as a lack of a clear roadmap or framework for the utilization of health research evidence, which can reduce the enthusiasm for utilizing research in health planning (33)(34).

The findings from the documents review checklist revealed a high level of integration of research evidence in health planning aligning with similar studies that underscore the importance of research-informed frameworks in public health planning (35)(36). The near-universal adoption of fiscal-year health plans (97.8%) and planning guidelines (98.9%) is consistent with global trends in evidence-based health planning, which emphasizes standardized planning processes (37). The presence of documents like CCHP and HF Planning guide mirrors findings from an urban strategic planning using blue-green infrastructure (38), who highlighted their role in improving health outcomes, the limited financial resources dedicated to health research in this study 22.5% suggests a common barrier. Similarly, the low inclusion of research coordinators (28.1%) echoes observations by a study designed for research approach during learning (39) on the limited interdisciplinary engagement in health planning. These gaps in budget allocation and

personnel highlight persistent structural challenges to fully operationalizing research evidence-backed plans, despite a broad commitment to evidence-based frameworks.

The study provided valuable insights into the factors influencing the use of health research evidence during health planning in Tanzania. The results from the binary logistic regression analysis highlight significant associations between region, level of education, and years of participation in health planning. Notably, region was a significant predictor, with participants from regions other than Kilimanjaro demonstrating varying likelihoods of using research evidence, as indicated by the odds ratios. Educational attainment also emerged as a key determinant; individuals with undergraduate degrees were over twice as likely to use health research evidence compared to those with a diploma, with statistically significant results (p-value = 0.029). However, while master's degree holders showed a similar trend, the relationship was not statistically significant (p-value = 0.0747). The analysis of years of participation revealed that those with more than 10 years of experience were 40% more likely to use health research evidence, though this association was not statistically significant. These findings align with other studies that emphasize the importance of education and experience in evidence-based health planning (25,27). The adjusted odds ratios and confidence intervals provide a nuanced understanding of the strength and variability of these associations, contributing to a growing body of literature on the determinants of research utilization in public health policy and planning.

The implications of these findings are essential for improving health planning in Tanzania. To enhance the use of health research evidence, interventions must address all three determinants: enhancing the capability of health planners through capacity building, improving opportunities by ensuring better access to human and non-human resources and organizational support, and fostering motivation by recognizing and rewarding health planners that use evidence-based decision-making. These strategies align with recommendations from similar studies in other LMICs, suggesting that a multifaceted approach including the development of middle range theory (MRT) outlining the main facilitators for utilization of evidence for malaria treatment policy in Uganda and the importance of measuring total brain activity in neuroimaging are essential for promoting the use of research in health planning (40)(41).

Conclusion

This study provides a comprehensive assessment of the determinants influencing the use of health research evidence in Tanzania, focusing on capability, opportunity, and motivation. The findings demonstrate that all three determinants significantly impact the behavior of health planners, highlighting those factors such as higher education levels, years of experience in health planning, and prior research knowledge contribute to greater use of health research evidence. Organizational infrastructure and institutional support further facilitate this process, while barriers include insufficient human resources, lack of funding, inadequate training, and perceptions that research is solely for academic purposes.

The study also reveals a high reliance on routine data and health planning guidelines, indicating the growing recognition of research evidence in health decision-making. However, addressing the barriers to research evidence use will require targeted interventions that strengthen the capability of health planners, improve access to resources and institutional support, and enhance motivation through incentives and professional development.

Future research should explore the long-term impact of these interventions, as well as contextual factors like political will and leadership, to fully understand how these determinants collectively shape health planning behavior. A multifaceted approach i.e. a generic framework for strengthening Knowledge Management in Tanzania (KMT) that will have a triad pathway: Capacity building on healthcare workers and researchers, infrastructure development, and funding that will improve access to health research database and resources within the healthcare system, and organizational support through the establishment of guidelines for the use of health research evidence in the decision-making process. This is essential for promoting the use of health research evidence, ultimately improving health outcomes in Tanzania and other low- and middle-income countries with similar challenges (42,43)

Abbreviations

CCHP	Comprehensive Council Health Planning
CHOP	Comprehensive Hospital Operational Plan
LGAs	Local Government Authorities
LMICs	Lower-and Middle-Income Countries

MoH	Ministry of Health
PO-RALG	President's Office-Regional Administration and Local Government
RHMT	Regional Health Management Team
SDGs	Sustainable Development Goals
UHC	Universal Health Coverage
WHO	World Health Organization

Acknowledgments

We would like to acknowledge the contribution of the University of Dodoma faculty members from the School of Nursing and Public Health for their input in the process of development of this manuscript. We would like to express our sincere thanks to the study facilities, the RHMTs, and the CHMTs for their support. We are grateful to the research assistants (Edmund Bunyaga, Theresia Ngungulu, Jestina Nyondo, Devina Mafuta Micky Masanyaji, and Ally Kinyaga) for their assistance during data collection.

Author Contributions

PK; Conceptualization, design of the work, data collection and analysis, preparing the manuscript draft, developing the methodology, results, discussion, and conclusion. RM, Conceptualization, critically reviewing the methodology and critically reviewing the manuscript, AK, Conceptualization, critically reviewing the methodology. All authors revised the work and approved the paper to be submitted for publication.

REFERENCES

1. Khalid FJI. The Impact of Poor Planning and Management on the Duration of Construction Projects. Multi-Knowl Electron Compr J Educ Sci Publ. 2019;(2):1–20.

2. Onwujekwe O, Etiaba E, Mbachu C, Arize I, Nwankwor C, Ezenwaka U, et al. Does improving the skills of researchers and decision-makers in health policy and systems research lead to enhanced evidence-based decision making in Nigeria?—A short term evaluation. Oelke N, editor. PLOS ONE. 2020 Sep 3;15(9):e0238365.

3. Henriksson DK, Peterson SS, Waiswa P, Fredriksson M. Decision-making in district health planning in Uganda: Does use of district-specific evidence matter? Health Res Policy Syst. 2019 Jun 6;17(1).

4. Kagoma P, Mongi R, Kapologwe NA, Kengia J, Kalolo A. Health research evidence: its current usage in health planning, determinants and readiness to use knowledge translation tools among health planning teams in Tanzania—an exploratory mixed-methods study protocol. BMJ Open. 2024 Jun;14(6):e081517.

5. Kengia JT, Kalolo A, Barash D, Chwa C, Hayirli TC, Kapologwe NA, et al. Research capacity, motivators and barriers to conducting research among healthcare providers in Tanzania's public health system: a mixed methods study. Hum Resour Health. 2023 Sep 5;21(1):73.

6. Van De Goor I, Hämäläinen RM, Syed A, Juel Lau C, Sandu P, Spitters H, et al. Determinants of evidence use in public health policy making: Results from a study across six EU countries. Health Policy. 2017 Mar;121(3):273–81.

7. Van Kammen J, Savigny DD, Sewankambo N. Using knowledge brokering to promote evidence-based policy-making: the need for support structures. Bull World Health Organ. 2006;84(8).

8. Guilbert JJ. The World Health Report 2006: Working together for health [1]. Educ Health Change Learn Pract. 2006;19(3):385–7.

9. Mboera LEG, Rumisha SF, Mbata D, Mremi IR, Lyimo EP, Joachim C. Data utilisation and factors influencing the performance of the health management information system in Tanzania. BMC Health Serv Res. 2021;21(1):4–11.

10. Michie S, van Stralen MM, West R. The behaviour change wheel: A new method for characterising and designing behaviour change interventions. Implement Sci. 2011 Apr 23;6(1).

11. De Leo A, Bayes S, Bloxsome D, Butt J. Exploring the usability of the COM-B model and Theoretical Domains Framework (TDF) to define the helpers of and hindrances to evidence-based practice in midwifery. Implement Sci Commun. 2021 Dec;2(1):7.

12. Kapologwe NA, Kalolo A, Kibusi SM, Chaula Z, Nswilla A, Teuscher T, et al. Understanding the implementation of Direct Health Facility Financing and its effect on health system performance in Tanzania: A non-controlled before and after mixed method study protocol. Health Res Policy Syst. 2019 Jan 30;17(1).

13. United THE, Of R. Comprehensive Council Health Planning Guideline Fifth Edition. 2019;

14. Ministry of Health. Guideline for Developing Comprehensive Hospital Operational Plan (CHOP) for Regional Referral Hospitals. 2016;(August).

15. Mwilike B, Nalwadda G, Kagawa M, Malima K, Mselle L, Horiuchi S. Knowledge of danger signs during pregnancy and subsequent healthcare seeking actions among women in Urban Tanzania: a cross-sectional study. BMC Pregnancy Childbirth. 2018 Dec;18(1):4.

16. Lyimo AA, Guo J, Mushy SE, Mwilike BE. Use of contraceptives and associated factors among male adolescents in rural secondary schools, Coast Region, Tanzania: a school-based cross-sectional study. Contracept Reprod Med. 2024 Mar 1;9(1):8.

17. Wahed T, Kaukab SST, Saha NC, Khan IA, Khanam F, Chowdhury F, et al. Knowledge of, attitudes toward, and preventive practices relating to cholera and oral cholera vaccine among urban high-risk groups: findings of a cross-sectional study in Dhaka, Bangladesh. BMC Public Health. 2013 Dec;13(1):242.

18. Subedi D. Explanatory sequential mixed method design as the third research community of knowledge claim. Am J Educ Res. 2016;4(7):570–7.

19. Taherdoost H. What Is the Best Response Scale for Survey and Questionnaire Design; Review of Different Lengths of Rating Scale / Attitude Scale / Likert Scale. 2019;8(1).

20. Shiyanbola OO, Rao D. Using an exploratory sequential mixed methods design to adapt an Illness Perception Questionnaire for African Americans with diabetes : the mixed data integration process. 2021;9(1):796–817.

21. Surucu L, Maslakci A. Validity and Reliability in Quantitative Research. Bus Manag Stud Int J. 2020;8(3):2694–726.

22. Jiyenze MK, Tundui C, Sirili N, Mollel H. Effects of implementing a health sector strategic plan : Eidence from Tanzania. East Afr Med J. 2022;99(9):5147–55.

23. Lavis J. Assessing country-level efforts to link research to action. Bull World Health Organ. 2006 Aug 1;84(8):620–8.

24. Hanney SR, Kanya L, Pokhrel S, Jones TH, Boaz A. How to strengthen a health research system: WHO's review, whose literature and who is providing leadership? Health Res Policy Syst. 2020 Dec;18(1):72.

25. Orton L, Lloyd-Williams F, Taylor-Robinson D, O'Flaherty M, Capewell S. The Use of Research Evidence in Public Health Decision Making Processes: Systematic Review. Ross JS, editor. PLoS ONE. 2011 Jul 26;6(7):e21704.

26. Innvær S, Vist G, Trommald M, Oxman A. Health policy-makers' perceptions of their use of evidence: a systematic review. J Health Serv Res Policy. 2002 Oct 1;7(4):239–44.

27. Oliver K, Innvar S, Lorenc T, Woodman J, Thomas J. A systematic review of barriers to and facilitators of the use of evidence by policymakers. BMC Health Serv Res. 2014 Dec;14(1):2.

28. Koon AD. A scoping review of the uses and institutionalisation of knowledge for health policy in low- and middle-income countries. 2020;

29. Lavis JN, Paulsen EJ, Oxman AD, Moynihan R. Evidence-informed health policy 2 – Survey of organizations that support the use of research evidence. Implement Sci. 2008;3(1):1–17.

30. Lavis JN, Wilson MG, Grimshaw JM, Haynes RB, Hanna S, Raina P, et al. Effects of an evidence service on health-system policy makers' use of research evidence: A protocol for a randomised controlled trial. 2011;

31. Moat KA, Lavis JN. 10 best resources for . . . evidence-informed health policy making.

32. Gavine A. Maximising the availability and use of high-quality evidence for policymaking: collaborative, targeted and efficient evidence reviews. PALGRAVE Commun. 2018;

33. Armstrong R, Waters E, Dobbins M, Anderson L, Moore L, Petticrew M, et al. Knowledge translation strategies to improve the use of evidence in public health decision making in local government: intervention design and implementation plan. Implement Sci. 2013 Dec;8(1):121.

34. Armstrong JS. Guidelines for Science: Evidence and Checklists.

35. Smith JD, Hasan M. Quantitative approaches for the evaluation of implementation research studies. Psychiatry Res. 2020 Jan;283:112521.

36. Roberts-Lewis SF, Baxter HA, Mein G, Quirke-McFarlane S, Leggat FJ, Garner HM, et al. The Use of Social Media for Dissemination of Research Evidence to Health and Social Care Practitioners: Protocol for a Systematic Review. JMIR Res Protoc. 2023 May 12;12:e45684.

37. Brown G, Kyttä M, Reed P. Using community surveys with participatory mapping to monitor comprehensive plan implementation. Landsc Urban Plan. 2022 Feb;218:104306.

38. Sörensen J, Persson AS, Olsson JA. A data management framework for strategic urban planning using blue-green infrastructure. J Environ Manage. 2021 Dec;299:113658.

39. Lim FV, Nguyen TTH. Design-based research approach for teacher learning: a case study from Singapore. ELT J. 2022 Oct 14;76(4):452–64.

40. Nabyonga-Orem J, Ssengooba F, Macq J, Criel B. Malaria treatment policy change in Uganda: what role did evidence play? 2014;

41. Hyder F, Rothman DL. Evidence for the importance of measuring total brain activity in neuroimaging.

42. Bennett NJ. Using perceptions as evidence to improve conservation and environmental management. Conserv Biol. 2016;30(3).

43. Hollingworth SA. What do we need to know? Data sources to support evidence-based decisions using health technology assessment in Ghana. . Epidemiology. 2020;

Supporting information captions

S1 Dataset used in the study

Figure

Click here to access/download;Figure;Fig1.docx

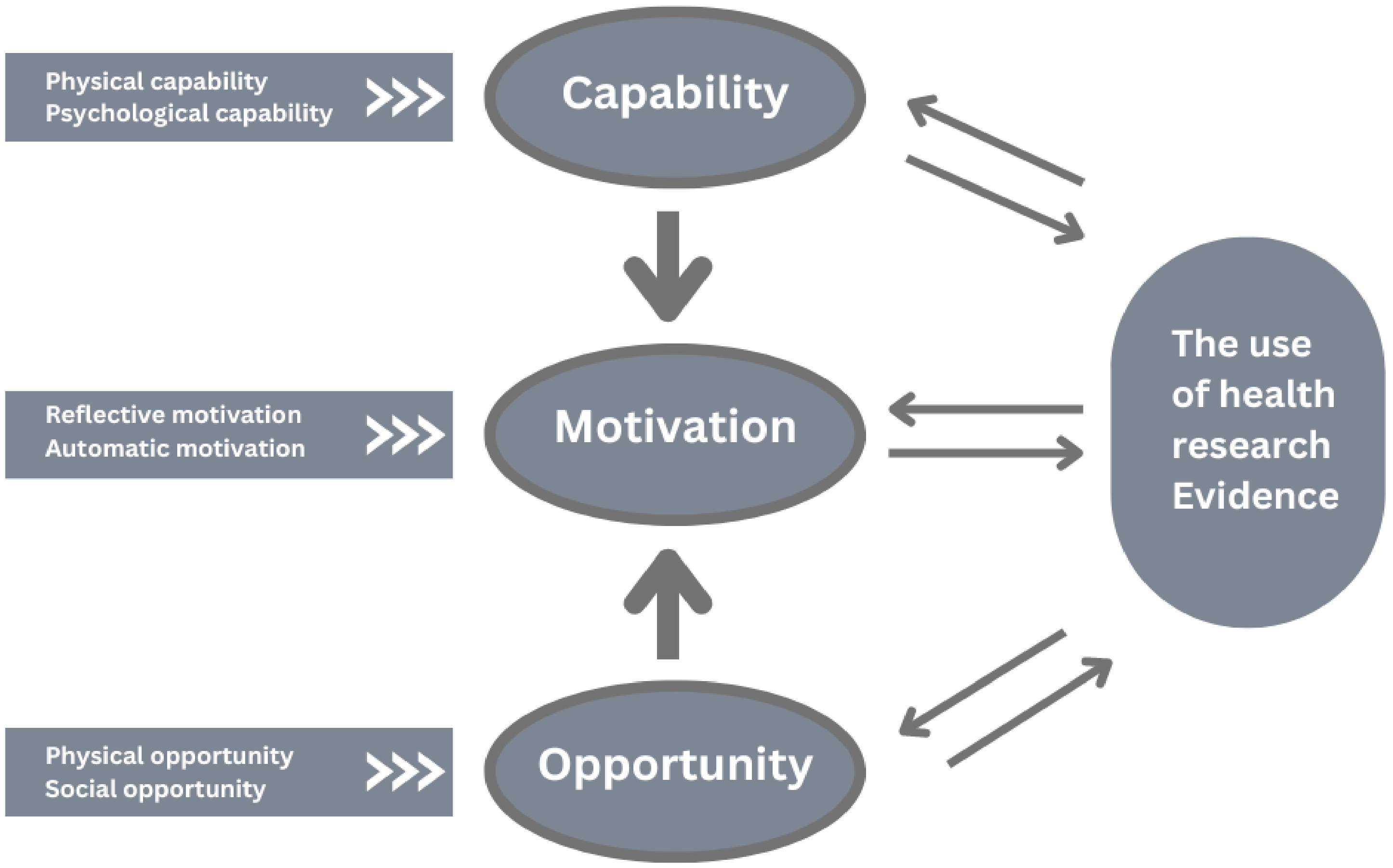

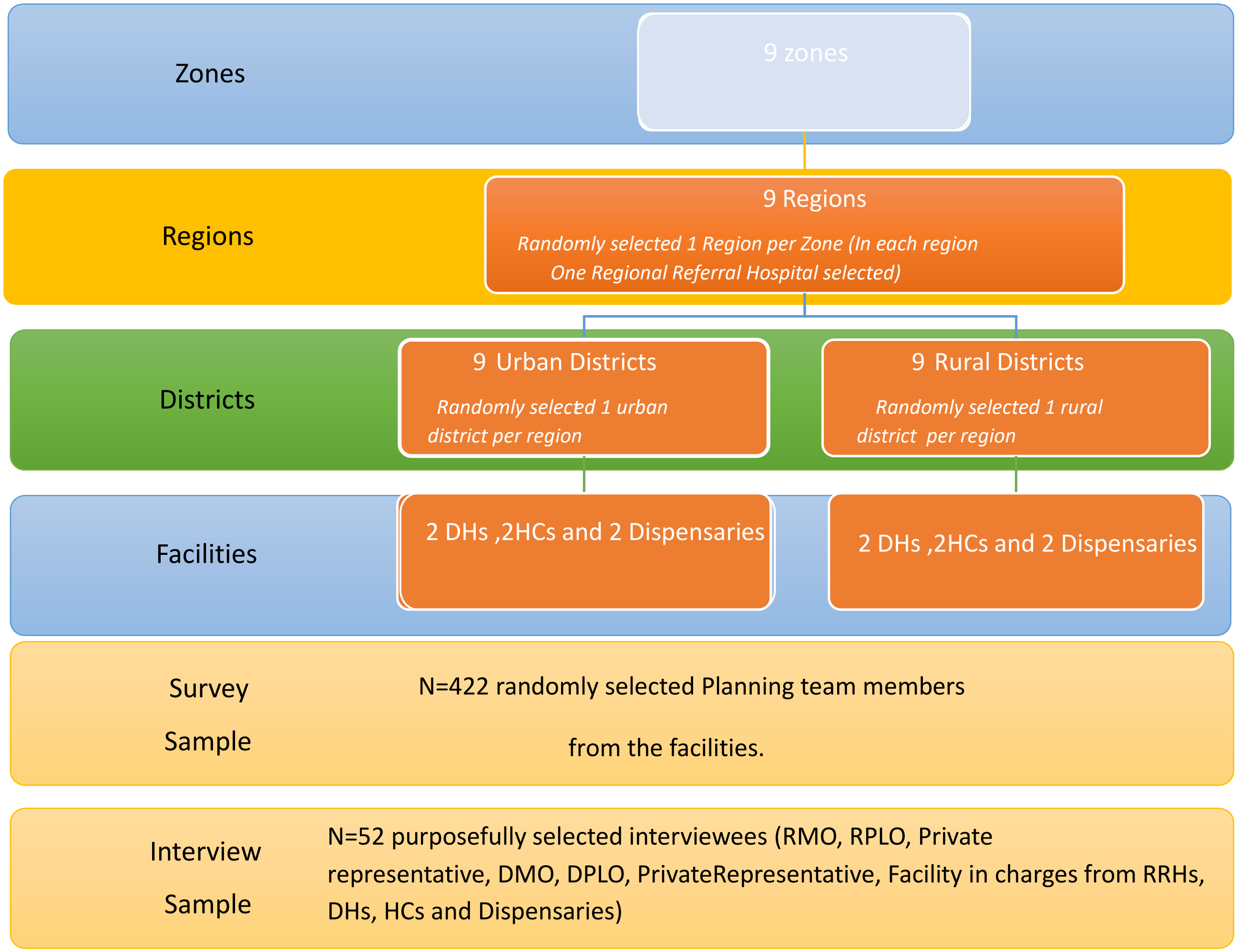

Fig.3 Sampling design

 Click here to access/download;Figure;Fig3.docx

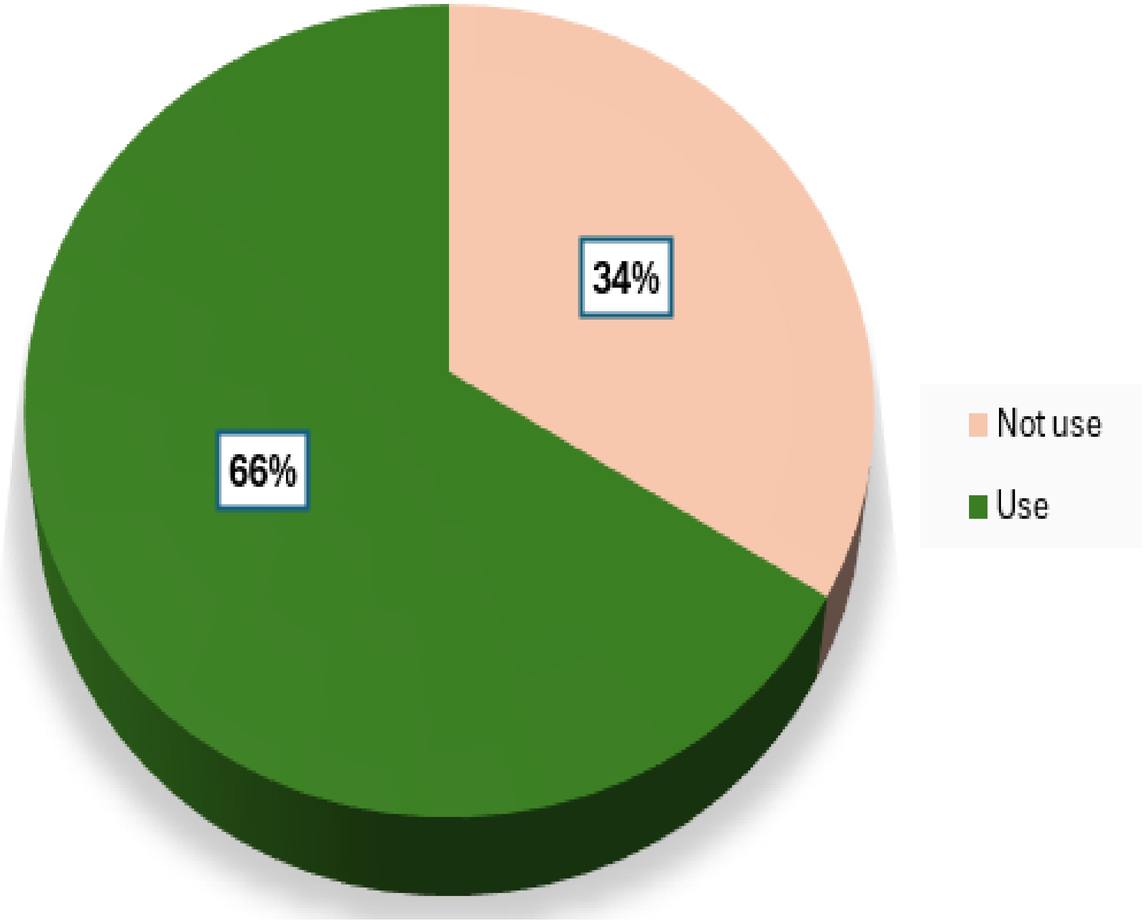

Click here to access/download
Supporting Information
Supporting information.docx

Analyzing the determinants for using health research evidence in health planning in Tanzania: a cross-sectional study.

Pius Kagoma*[1&2,] Richard Mongi[1], Albino Kalolo[3, 4]

[1]Department of Public Health and Community Nursing, the University of Dodoma, Dodoma, Tanzania;
[2]Division of Health, Social Welfare and Nutrition Services, President's Office-Regional Administration Local Government, Dodoma, Tanzania;
[3]Department of Public Health, St. Francis University College of Health and Allied Sciences, Morogoro, Tanzania;
[4]Centre for Reforms, Innovation, Health Policies, and Implementation Research, Dodoma, Tanzania.

*Corresponding author: Pius Kagoma; +255 (0) 76 5909942; P.O. Box 1923 Dodoma, Tanzania; piuskagoma@gmail.com

Richard Mongi; +255 (0) 67 6953510; P.O. Box 259, Dodoma, Tanzania; richiemongi@gmail.com

Albino Kalolo; +255 (0) 764340819; P.O. Box 175, Ifakara, Morogoro, Tanzania; kaloloa@gmail.com

Abstract

Introduction: Achieving Universal Health Coverage (UHC) requires utilizing research evidence to inform the health planning~~decision making process~~. However, little information is available on the determinants for using research evidence in planning in Lower Middle-Income Countries (LMICs), including Tanzania. This study ~~paper~~ aims to investigate the determinants of using health research evidence in health planning in Tanzania.

Materials and methods: This quantitative study employed a cross-sectional ~~study~~ design. Data on health research evidence and the factors influencing its use ~~Data on health research evidence and its determinants~~ were collected using a structured questionnaire from 422

respondents within ~~from~~ 9 regions of Tanzania from October to December 2023. The data were analyzed using STATA version 18 for descriptive and inferential statistics. The association between categorical variables was assessed using a chi-square test, while regression analysis was conducted to identify determinants, both at a 95% confidence level. ~~The association between variables was determined using a chi-square test at a 95% confidence level.~~

Results: The study revealed that 270 (66.2%) of the health planning team members ~~participants~~ strongly agreed to use health research evidence during planning. However, significant barriers were identified, including lack of dissemination (74.5%), inadequate human and non-human resources (70.0%), and insufficient knowledge and training in research (63.7%). A chi-square test confirmed significant associations between these barriers and the reduced use of research evidence ($p<0.05$). Conversely, more than 70% of respondents identified opportunities such as the availability of research coordinators, university partnerships, available research budgets, and internet access, all significantly associated with increased health research evidence use. More than 50% of participants reported motivational factors that like continuous quality improvement agenda in the healthcare sector, availability of short and long-term courses, on-the-job training, and provision of incentives like extra duty allowances were positively linked to research utilization.

Conclusion: The study found that 66.2% of health planning team members ~~participants~~ used health research evidence in planning, but barriers like lack of dissemination, resource shortages, and inadequate training persisted. Interventions should focus on improving dissemination, resources, and training. Future research should explore strategies for enhancing these interventions.

Keywords: Determinants, Research evidence use, Health planning, Tanzania.

Formatted: Font: (Default) Times New Roman, 12 pt

Formatted: Font: (Default) Times New Roman, 12 pt

Formatted: Font: (Default) Times New Roman, 12 pt

Formatted: Font: (Default) Times New Roman, 12 pt

INTRODUCTION

Health research evidence plays an essential role in shaping effective health policies and interventions, which are crucial for improving public health outcomes (1). In resource-limited

settings like Tanzania, where health challenges are complex and resources are constrained, the strategic use of research evidence is particularly important. It can guide decision-makers in identifying priority areas, optimizing resource allocation, and implementing interventions that are both cost-effective and impactful. Moreover, the use of evidence can help health planning teams avoid repeating past failures and introduce innovative solutions (2). Despite its recognized importance, the extent to which health research evidence is utilized by health planning teams in Tanzania remains unclear. Understanding the factors that influence this utilization is essential for promoting evidence-based decision-making, which is critical for achieving the ambitious health targets set forth in the Sustainable Development Goals (SDGs), particularly Universal Health Coverage (UHC)(3).

Efforts to promote the integration of research evidence into health planning and decision-making have been made in various settings. These include the establishment of knowledge translation platforms, research dissemination strategies, health conferences, and capacity-building initiatives targeting health planning teams. However, the success of these efforts varies significantly across contexts, largely depending on factors such as institutional support, the capacity of the health workforce, and access to reliable data. In Tanzania, while some initiatives aim to bridge the gap between research and practice, the actual impact of these efforts on health planning remains under-explored.

Research across different settings has demonstrated that the use of health research evidence in decision-making is influenced by a variety of factors. These include organizational culture, the availability of resources, the expertise of the planning teams, and access to research outputs [4]. In a study conducted by Kagoma et al (4), it was found that health planning teams in Tanzania consist of professionals from various disciplines, including Administrators (9%), Medical doctors (25.36%), Nurses (23.46%), Laboratory scientists (8.53%), Pharmacists (6.64%), Radiographers (1.66%), Environmental health officers (3.08%), Nutrition officers (3.08%), Social welfare officers (4.74%), and others (14.46%). Their primary responsibility is to plan, implement, and evaluate health interventions [4]. However, several factors may limit their ability to fully integrate research evidence into their work (5). These include inadequate dissemination of research findings, lack of access to research evidence, limited skills in interpreting research data, and insufficient organizational support (3,6–8).

While previous studies have explored various factors influencing the use of health research evidence in policy and planning, there remains a gap in understanding the specific determinants that affect the utilization of health research evidence among health planning teams in Tanzania(4,9). This study seeks to fill this gap by analyzing the key factors that influence evidence use among these teams at both the regional and council levels, guided by the COM-B Model (Capability, Opportunity, and Motivation) as the theoretical framework (10), which has been widely used in understanding evidence-based decision-making processes. The COM-B model is particularly suited to the Tanzanian context because it focuses on the essential components of behavior change Capability, Opportunity, and Motivation which are critical for understanding and addressing the factors influencing the use of health research evidence by planning teams. By examining these elements, the model helps identify specific barriers and enablers that can be targeted to improve evidence-based decision-making in Tanzania's resource-limited. By applying this theoretical lens, the study aims to provide a deeper understanding of the barriers and facilitators to using research evidence in health planning, with the following specific objectives: 1) Analyze the current usage of health research evidence among planning team members at the regional and council levels, 2) Analyze the capacity of health planning team members to utilize health research evidence, 3) Identify opportunities for enhancing the use of health research evidence in health planning, 4) Identify opportunities for enhancing the use of health research evidence in health planning, 5) Explore the motivations for using health research evidence among health planning members at both regional and council levels. The findings of this study are expected to contribute to the development of strategies that enhance the integration of research evidence into health planning processes, ultimately leading to more effective health interventions and improved health outcomes in Tanzania.

Conceptual framework

The conceptual framework for this study was developed after several consultative meetings with different stakeholders and researchers. The theory was adopted and modified from the (Capability, Opportunity, and Motivation) COM-B model. The COM- B Model at the center of a proposed framework is a behavior system involving three essential conditions: Capability, Opportunity, and Motivation, what we term the ‘COM-B system(10,11). This study investigated

how the capability, opportunity, and motivation determinants can influence the behavior of the use of health research evidence during health planning as shown in Figure 1

…………………………………………………………………

Insert figure 1

………………………………………………………………….

Figure 1: The domains of the COM-B Model modified from Michie et al,.2011(10)

Materials and Methods

Study setting

This study was conducted between October and December 2023 in the United Republic of Tanzania, a Lower and Middle low-income country located in East Africa, with a population of about 62 million people. The allocated budget for the country's Ministry of Health in 2023/2024 was estimated to be 443.6 million US dollars, with 1.2 million US dollars (0.27%) allocated for evidence production. The health research evidence users in Tanzania involve three important Ministries, namely; the Ministry of Health (MoH), The President's Office Regional Administration and Local Government (PO-RALG), and Health management. The health evidence producers in Tanzania are the National Institute for Medical Research (NIMR), the Tanzania Commission for Science and Technology (COSTECH), Public and Private Universities, Health related institutions or authorities, local and international non-governmental organizations (NGOs), and civil society organizations as shown in Table 1.

Table 1: A list of health research evidence producers and users in Tanzania

SN	Evidence users

1	Ministry of Health
2	President’s Office Regional Administration and Local Government
3	Ministry of Education Science and Technology (MEST)
4	Prime Minister’s Office
5	Regional Health Management Teams
6	Council Health Management Teams
7	Healthcare workers
8	Non-Governmental Organizations (NGOs)
9	Civil Society Organizations (CSOs)
10	Private Sector
**Evidence producers**	
1	National Institute for Medical Research (NIMR)
2	Tanzania Commission for Science and Technology (COSTECH)
3	Public Universities; The Muhimbili University of Health and Allied Sciences (MUHAS), University of Dar es Salaam (UDSM), University of Dodoma (UDOM)
4	Private Universities; Catholic University of Health and Allied Sciences (CUHAS), Kilimanjaro Christian Medical Centre (KCMC), Hubert Kairuki Memorial University (HKMU), St. John’s University of Tanzania (SJUT), St. Augustine University of Tanzania (SAUT), Kampala International University (KIU), St. Francis University College of Health and Allied Sciences (SFUCHAS)
5	Kilimanjaro Clinical Research Institute (KCRI)
6	Ifakara Health Institute (IHI)
7	Non-Governmental Organizations (NGOs)
8	Civil society organizations (CSOs)
9	Private sector

The study sites

The study site was eighteen (18) Councils of the Local Government Authorities within the nine (9) regions out of 26 Regions of Tanzania Mainland from nine (9) geographical zones as summarized in Table 2 ~~and Figure 2~~. The nine zones are selected to seek the country’s geographical representation. Together, these regions have a total population of 21,119,700,

which represents 35.7% of the Tanzanian population. These regions are heterogeneous in population size, distribution of health facilities, human resources for health, and institutions carrying out health research activities. A similar approach has been used in major Tanzanian health studies(4,5,12); This approach provided a comprehensive understanding of the status quo for the use of health research evidence in health planning, and heterogeneous study population for random sampling hence acting as a representative snapshot of all regions in Tanzania. The study was conducted in 63 randomly selected health facilities, nine (9) Regional Referral Hospitals (RRHs), eighteen (18) District Hospitals (DHs), Eighteen (18) Health Centres (HCs), and eighteen (18) Dispensaries. To ensure the inclusion of urban and rural health facilities, stratification was conducted followed by random sampling.

Table 2: Summary of study sites

ZONE	REGION	COUNCIL
Northern		Siha District
	Kilimanjaro	Moshi Municipal
Central zone		Chamwino District
	Dodoma	Dodoma City
Dar es Salaam		Kigamboni Municipal
	Dar es Salaam	Dar es Salaam City
Eastern		Morogoro Municipal
	Mororgoro	Gairo District
Western		Kigoma District
	Kigoma	Kigoma Municipal
Southwest highland		Mbeya City
	Mbeya	Kyela District
Lake		Kwimba District
	Mwanza	Mwanza City
Southern		Mtwara Municipal
	Mtwara	Nanyamba
Southern Highland	Iringa	Iringa Municipal
		Iringa District

Insert figure 2

Figure 2: Distribution of sampled facilities by zones, regions, and districts

Study population

This study involved members of health planning teams at the regional and council levels in Tanzania. Eligible participants were those holding health planning roles in healthcare facilities or administrative positions, with at least one year of experience in health planning. Individuals who were not part of the health planning team or had less than one year of experience were excluded from the study.

Formatted: Right: 0", Line spacing: 1.5 lines

The health planning landscape in Tanzania

The health planning process in Tanzania is conducted at two levels; At the Council level, the health facility prepares its plans as well and the Council Health Management Team (CHMT) prepares its plan, whereby plans from the facility and CHMT are later consolidated to form Comprehensive Council plan (CCHP). The preparation of health facility plans is guided by the Health facility planning guidelines and CCHP guidelines.(13). At the regional level, the Regional Health Management Teams (RHMTs) prepare their plans using the RHMT planning guide and the Regional Referral Hospitals (RRHs) plans using the Comprehensive Operational Plan Guide (CHOP)(14). When the plans are completed are sent to the Ministry of Health and President's Office-Regional Administration and Local Government for final assessment before being sent to the Ministry of Finance for funding. At all levels, we are planning to use the guidelines and the routine data collected from each level.(14).

Study Design

The study employs adopts a quantitative cross-sectional design focusing on nine regions of Tanzania. (15,16).

Sample size and Sampling procedures

The sample size involved the health planning team members from the RRHs, DHs, HCs, and Dispensaries randomly selected from the nine regions. The sample size was 422 calculated from

the Cochrane formula (1977). To date, in Tanzania there is no cited reference for the percentage of the use of health research evidence in health planning therefore the assumed ~~marginal error of~~proportion of 50% was used (17).

$$n = \frac{Z^2 * p(1-p)}{d^2} \text{ (Cochran (1977))}$$

Where by

N-sample size

Z-confidence interval 95% 1.96, P-proportional from previous study d = margin of error, which is approximately 5% Thus,

$$\mathbf{n = \frac{(1.96)^2 \times 50(100-50)}{(5)^2}}$$

=**384** including 10% of non-response sample 384 plus 10%=38+384=422

This study employed a multistage sampling technique for the selection of the study units as summarized in Figure 2~~3~~. The sampling stages are zones, regions, councils, and public primary health facilities. The first stage was a random selection of one region from each of the nine Zones of the country. In the second stage, in each selected region, councils were clustered into rural and urban, and then one rural and one urban council was ~~will be~~ selected from each region followed by a random selection of the health facilities, see Figure 3. The technique is convenient for studying large and diverse populations (5). The sampling stages were zones, regions, councils, and public primary health facilities.

…………………………………………………..

Insert figure 2~~3~~

……………………………………………………

Measurement of variables

~~Variables and their measurements.~~

Formatted: Font: (Default) Times New Roman, 12 pt, Bold

Formatted: No bullets or numbering

Dependent variable

The dependent variable of this ~~study i.e. the~~study, the use of health research evidence was measured using a ~~obtained from~~ a set of four (4) questions adapted from previous study(18) ~~that were measured~~. The questions were a mixture of binary and multiple-choice questions. Those questions were divided into four areas. The mean score was calculated from those sets of questions; the score had a value of 0 and 1. Those respondents who scored 1 were regarded to have used the health research evidence, while those who scored 0 ~~were regarded to~~ have not used the health research evidence. For multiple choice questions, the respondents were provided with a set of predefined options, allowing them to select the one that best represents their response, which will capture varying degrees or categories on the use of health research evidence.

Independent Variables

The independent variables in this study encompass numerous factors including demographic information such as sex, age, and education level; professional attributes such as position within the health facility and years of schooling; and contextual elements including the type of health facility, stakeholders group represented district~~,~~ and region. The study aimed to gain insights into the determinants that influence the use of health research evidence during health planning. The determinants had a combination of three constructs which are, capability determinants, opportunity determinants, and motivation determinants both derived from the COM-B Model.

Capability determinants were Assessed using 12 Likert-scale questions (19) (1 = strongly agree to 5 = strongly disagree) and one multiple-choice question. Mean scores and standard deviations were calculated, and chi-square tests were used for categorical responses. ~~were obtained from a set of twelve (12) Likert scale questions with a scale of 1 to 5 (1 = strongly agree, 2 = somewhat agree, 3 = neither agree nor disagree, 4 = somewhat disagree, 5 = strongly disagree) and one multiple choice question. The mean and standard deviation of the responses to assess central tendency and variability were calculated. For a multiple-choice question, the frequency distribution of each response category was analyzed. Additionally, a chi-square test~~

~~was calculated to determine if the distribution of responses was significantly different from what would be expected by chance.~~

Opportunity determinants~~,~~ were evaluated through 17 Likert-scale questions, with responses assigned numerical values (1 to 5). Mean or median scores were used to quantify overall tendencies, and multiple regression analysis was conducted to determine their influence on the dependent variable. ~~were obtained from a set of seventeen (17) Likert scale questions with a scale of 1 to 5 (1 = strongly agree, 2 = somewhat agree, 3 = neither agree nor disagree, 4 = somewhat disagree, 5 = strongly disagree). We assigned numerical values (1 to 5) to these responses, where lower values indicate stronger agreement. Next, the mean or median was calculated of these numerical values to quantify the overall tendency. The multiple regression analysis was used to assess the relationship between the independent variable and dependent variables.~~

Formatted: Font: Not Bold

Motivation determinants **were** measured using 8 Likert-scale questions, analyzed similarly to opportunity determinants, with multiple regression analysis applied to assess relationships with the dependent variable.~~were obtained from a set of eight (8) Likert scale questions with a scale of 1 to 5 (1 = strongly agree, 2 = somewhat agree, 3 = neither agree nor disagree, 4 = somewhat disagree, 5 = strongly disagree). We assigned numerical values (1 to 5) to these responses, where lower values indicate stronger agreement. Next, the mean or median was calculated of these numerical values to quantify the overall tendency. The multiple regression analysis was used to assess the relationship between the independent variable and dependent variables.~~

Data collection procedures and tools

Quantitative data was collected face to face person by using the Swahili version of the survey after translation of the English version of the survey and document review checklist (see supplemental file) to planning team members at national, regional, council, and health facility levels. The questions from the tool were adopted and modified from previous studies (18,20). The survey collected information on the use of health research evidence and its determinants from the sampled participants using Open Data Kit software (ODK).

The document review checklist was used to guide the document review to see whether the available plans have any evidence of being prepared using health research evidence. The

checklist contains a list of questions that will help the review of the plans made at the facility, council, regional, and national levels. The checklist is used to collect information on the available health plans if they are prepared by using health research evidence. Data was collected by a research assistant, who was trained for three days in data collection methods, tools, and ethics. They were selected according to their experience in the health system. Pilot testing of the tools was conducted at Dodoma RHMT and Chamwino District Council at Chamwino CHMT, Chamwino Council Hospital, Chamwino Health Centre, and Buigiri Dispensary to enhance the validity, reliability, and effectiveness of the tool in capturing the intended information.

Data analysis

Responses were reported on all survey items in all response categories and summarized using frequencies and percentages for categorical variables and means and standard deviation for non-categorical variables were computed. Given that the outcome variable had two categories (0=Negative, 1=Positive), A binary logistic regression model was used to assess factors associated with the perception of implementers on the prime vendor system. The model results are regression parameter estimates and odds ratios (OR). The data analysis was conducted using STATA version 18, and the significance of all statistical tests was established at a 5% significance level.

Data validity and reliability

To ensure validity and reliability, the validity of quantitative data collection tools was reviewed by independent subject matter experts from the University of Dodoma (UDOM). The reliability assessment of the questionnaire was done using the internal consistency test, with the alpha reliability coefficient being the statistic. (21,22). The range of the alpha coefficient, also known as Cronbach's alpha was 0.71 acceptable to this study. Moreover, exploratory factor analysis to establish the construct validity of the questionnaire and pilot study conducted at Chamwino DC before field data collection to ensure clarity of the data collection tool.

Ethics and Dissemination

The study was granted ethical approval by the University of Dodoma Ethical Committee with **Ref. No. MA.84/261/102/'A'/64/91.** Permission to conduct the study and consent to participate

in the study was sought from relevant authorities and participants, respectively. Participants received information about the purpose of the study and data protection. Permission to conduct the study was also obtained from the Local Government authorities and the Ministry of Health. Written informed consent was obtained from all participants before their involvement in the study. Participants were informed of their right to withdraw from the study at any time, and their participation was entirely voluntary. Confidentiality was strictly maintained by ensuring the anonymity of all documents containing participants' information. The findings of the study were disseminated to relevant stakeholders through collaborative communication with the Ministry of Health, RHMT/CHMT, Tanzania Health Summit as well as publications to target researchers, practitioners, implementers, and policymakers.

Results

The finding from this study is presented in three parts using tables, percentages, and charts, Part one shows the respondents' demographic characteristics and stakeholder group. Part two shows the proportion of the respondents who reported using Health research evidence in health planning and, part three shows the association between health research use in planning and the COM-B constructs (capability, Motivation, and opportunity).

Demographic characteristics

Table 1 describes the respondents' characteristics and the stakeholder group they represent. There was an almost equal number of female 50.2 % and male 49.8% respondents with a mean age of 39.51 ±7.86years old. Most of them (42.7%) had undergraduate education followed by a Diploma (36.3%) and less than one-third (14.0%) had a master's degree and above. Few (7.1%) respondents ‘had certificate-level education. Professional background results revealed that the majority 107 (25.4%) of the respondents were medical doctors followed by nurses 99 (23.5%). Physiotherapists and panning offices were very few each accounting for 0. 24% of the respondents.

Table 3: Demographic characteristics of respondents

Variable	Frequency	Percent

Formatted Table

**Age**		
<25	3	0.7
26-35	159	37.7
36-45	156	37.0
46-55	93	22.4
>46	11	2.6
**Region**		
Mbeya	48	11.4
Dodoma	50	11.9
Iringa	41	9.7
Mtwara	49	11.6
Mwanza	50	11.85
Kigoma	51	12.1
Kilimanjaro	45	10.7
Morogoro	48	11.4
Dar es salaam	40	9.5
**Type of health facility**		
Dispensary	37	8.8
Health center	82	19.4
Council Hospital	97	23.0
Regional referral hospital	81	19.2
RHMT	87	20.6
CHMT	38	9.0
Gender		
Male	210	49.76
Female	212	50.24
**Professional background**		
Doctor	107	25.36
Nurse	99	23.46
Laboratory scientist	36	8.53
Pharmacist	28	6.64
Radiographer	7	1.66
Environmental Health officer	13	3.08
Nutrition officer	13	3.08
Social Welfare Officer	20	4.74
Physiotherapist	1	0.24
Biomedical engineer	3	0.71
Planning officer	1	0.24
Health Secretary	38	9.00
Other	56	13.3
**Stakeholders' group**		
RHMT	49	11.6
HMT	280	66.4
CHMT	90	21.3
FBO	1	0.2

HGFC	2	0.5
**Highest level of education**		
Certificate	30	7.1
Diploma Certificate	153	36.3
Undergraduate degree	180	42.7
Master’s Degree	56	13.3
PhD	3	0.7
**The position holds currently in health planning.**		
Chairperson	30	7.1
Secretary	45	10.7
Technical Advisor	11	2.6
Member	335	79.4
Other	1	0.2
**Years participate**		
<5	283	67.1
6-10	103	24.4
>10	36	8.5

Proportion of the use of health research evidence

Table 4 provides data on the use of health research evidence in a particular context. It shows that the majority of participants (96.7%) have used routine data, with 49.5% reporting a high extent of use. Additionally, evidence from routine data was the most commonly used type of evidence during health planning, with 98.8% of participants reporting its use. Most participants (66.2%) have used health research evidence as shown in Figure 34, and a significant majority (98.8%) have used health planning guidelines. Moreover, the majority of participants (98.8%) also indicated the importance of the use of health research evidence, with 82.6% considering it very important. These findings suggest a strong reliance on routine data and a high recognition of the importance of health research evidence in the context studied.

Table 4: The proportion of the use of health research evidence during health planning

Variable	Frequency	Percent
**Ever used routine data**		
No	14	3.3
Yes	408	96.7
**Extent of use of routine data**		
Low	41	10.1
Medium	165	40.4
High	202	49.5

**Type of evidence used during health planning (Multiple response)**		
Evidence from routine data	403	98.8
Policy documents	280	68.6
Research publications (general)	133	32.6
Systematic reviews/ meta-analysis	41	10.1
Randomized Control trials	17	4.2
Experimental studies	30	7.4
Non-experimental studies	20	4.9
Expert opinions	271	66.4
Policy beliefs	162	39.7
Other	17	4.2
**Use of health research evidence**		
No	138	33.8
Yes	270	66.2
**Use of the Health planning guidelines**		
No	5	1.2
Yes	403	98.8
**Use of the Ruling party manifesto**		
No	44	10.8
Yes	364	89.2
**Use of the Policy documents**		
No	137	33.6
Yes	271	66.4
**Importance of the use of health research evidence**		
No	5	1.2
Yes	403	98.8
**Level of importance of the use of health research evidence**		
Very unimportant	5	1.2
Unimportant	2	0.5
Neutral	14	3.4
Important	50	12.3
Very important	337	82.6

…………………………………………………………………………………

Insert figure 34

…………………………………………………………………………………..

Figure 4: Proportion of the use of health research evidence.

The factors associated with the use of health research evidence during health planning.

The table presents the results of a binary logistic regression analysis examining factors associated with the use of research evidence during health planning. The analysis includes variables such as age, region, type of health facility, gender, professional background, stakeholder group, highest level of education, position held in health planning, and years of participation. The adjusted odds ratios (OR), confidence intervals (CI), and p-values are provided to indicate the strength and statistical significance of associations.

Significant Variables and Interpretation

Region: The analysis compares various regions to the reference category, Kilimanjaro. The odds ratios (OR) show how likely individuals from other regions are to use health research evidence in health planning compared to those from Kilimanjaro. For instance, if a region has an OR greater than 1, individuals from that region are more likely to use research evidence than those from Kilimanjaro. If the OR is less than 1, they are less likely. The p-values indicate whether these differences are statistically significant.

Formatted: No bullets or numbering

Level of Education: Compared to individuals with a diploma (reference category), those with higher education levels were more likely to use research evidence:

Formatted: Indent: Left: 0.5", No bullets or numbering

- Undergraduate degree (OR = 2.396, CI: [1.094, 5.249], p-value = 0.0290): Those with an undergraduate degree are over twice as likely to use research evidence compared to diploma holders.
- Master's degree (OR = 2.278, CI: [0.921, 5.631], p-value = 0.0747): Similar to undergraduates, master's degree holders are also more likely to use research evidence in health planning although were not significant.

These OR values reflect the increased likelihood of individuals with higher education levels using research evidence compared to the reference group, with p-values indicating the strength of this relationship.

Years of Participation:

Formatted: Indent: Left: 0.5", No bullets or numbering

- 6–10 years (OR = 1.092, CI: [0.677,1.761], p-value = 0.7184): Individuals with 6–10 years of participation in health planning show a higher likelihood of using research evidence compared to those with fewer years, though this was not statistically significant.
- More than 10 years (OR = 1.399, CI: [0.648,3.019], p-value = 0.3924): Those with over 10 years of participation have a 40% higher likelihood of using research evidence compared to those with fewer years of experience, and this difference was also not statistically significant.

The adjusted odds ratios reflect the likelihood of each category of the variable influencing the use of research evidence, controlling for other variables in the model. Significance is determined by both the size of the OR and the p-value, with confidence intervals providing a range within which the true effect likely falls.

Table 5: Binary logistic regression for factors associated with the use of research evidence during health planning.

Variable	Not use	Use	Unadjusted logistic analysis		Adjusted logistic analysis	
	N (%)	N (%)	OR [95%CI]	p-value	OR [95%CI]	p-value
**Age**						
<35	52(32.1)	110(67.9)	ref			
36-45	56(35.9)	100(64.1)	0.844[0.530,1.343]	0.4747		
>45	35(33.7)	69(66.4)	0.932[0.552,1.573]	0.7919		
**Region**						
Kilimanjaro	26(57.8)	19(42.2)	ref		ref	
Mbeya	17(35.4)	31(64.6)	2.495[1.081,5.760]	0.0322	2.604[1.074,6.315]	0.0342
Dodoma	17(34.0)	33(66.0)	2.656[1.156,6.104]	0.0214	2.514[1.053,6.001]	0.0378
Iringa	10(24.4)	31(75.6)	4.242[1.680,10.71]	0.0022	4.707[1.772,12.499]	0.0019
Mtwara	21(42.9)	28(57.1)	1.825[0.805,4.137]	0.1499	1.801[0.727,4.466]	0.2039

Mwanza	12(24.0)	38(76.0)	4.333[1.801,10.43]	0.0011	4.794[1.893,12.140]	0.0009
Kigoma	10(19.6)	41(80.4)	5.611[2.259,13.94]	0.0002	5.922[2.224,15.769]	0.0004
Morogoro	17(35.4)	31(64.6)	2.495[1.081,5.760]	0.0322	2.180[0.901,5.274]	0.0839
Dar es salaam	13(32.5)	27(67.5)	2.842[1.170,6.904]	0.0211	3.229[1.211,8.611]	0.0192
**Type of health facility**						
Dispensary	17(46.0)	20(54.1)	ref		ref	
Health center	25(30.5)	57(69.5)	1.938[0.871,4.311]	0.1048	2.029[0.829,4.964]	0.1211
Council Hospital	45(46.4)	52(53.6)	0.982[0.459,2.100]	0.9631	0.809[0.323,2.026]	0.6511
RRH	19(23.5)	62(76.5)	2.774[1.214,6.335]	0.0155	2.663[1.895,7.920]	0.0383
RHMT	27(31.0)	60(69.0)	1.889[0.857,4.163]	0.1147	0.350[0.031,3.902]	0.3932
CHMT	10(26.3)	28(73.7)	2.380[0.903,6.273]	0.0795	5.647[0.602,52.94]	0.3167
Gender						
Male	71(33.8)	139(66.2)	1.007[0.673,1.507]	0.9736		
Female	72(34.0)	140(66.0)	ref			
**Professional background**						
Doctor	37(34.6)	70(65.4)	ref			
Nurse	35(35.4)	64(64.7)	0.967[0.545,1.714]	0.9073		
Lab scientist &Pharmacist	25(35.2)	46(64.8)	0.973[0.518,1.825]	0.9310		
Non-medical	28(31.5)	61(68.5)	1.152[0.633,2.096]	0.6444		
Other	18(32.1)	38(67.7)	1.116[0.561,2.220]	0.7548		
**Stakeholders' group**						
RHMT	13(26.5)	36(73.5)	1.609[0.816,3.171]	0.1697	0.843[0.177,4.012]	0.8297
CHMT	26(28.9)	64(71.1)	1.430[0.854,2.396]	0.1741	5.647[0.602,52.93]	0.1295
HMT	104(36.8)	179(63.3)	ref		ref	
**Highest level of education**						
Certificate	15(50.0)	15(50.0)	ref		ref	
Diploma	57(37.3)	96(62.8)	1.684[0.767,3.700]	0.1943	1.609[0.670,3.867]	0.2874
Undergraduate	53(29.4)	127(70.6)	2.396[1.094,5.249]	0.0290	1.824[0.710,4.685]	0.2120
Master’s Degree	18(30.5)	41(69.5)	2.278[0.921,5.631]	0.0747	2.082[0.684,6.337]	0.1968
**Position holds currently in health planning**						
Secretary	19(42.2)	26(57.8)	ref		ref	
Chairperson	8(26.7)	22(73.3)	2.009[0.738,5.475]	0.1724	1.933[0.647,5.780]	0.2381
Technical	4(36.4)	7(63.6)	1.279[0.327,5.000]	0.7236	0.789[0.173,3.599]	0.7600
Member	112(33.3)	224(66.7)	1.462[0.776,2.754]	0.2404	1.328[0.651,2.708]	0.4354
**Years participate**						
<5	99(35.0)	184(65.0)	ref			
6-10	34(33.0)	69(67.0)	1.092[0.677,1.761]	0.7184		
>10	10(27.8)	26(72.2)	1.399[0.648,3.019]	0.3924		

Capability: The knowledge and skills of health research evidence use

Table 6 presents the findings from a survey on the capacity to use health research evidence (knowledge and skills). Each row in the table represents a different variable related to the use of health research evidence, and the columns display the responses categorized into five levels of importance: Very Unimportant (VUIM), Unimportant (UIM), Neutral, Important (IM), and Very Important (VIM).

From the data, it's clear that the majority of respondents perceive the use of health research evidence as very important or important across the various variables. In the variable "Gives the latest information," the majority of respondents rated it as Very Important (65.7%) or Important (24.5%). Similarly, in the variable "Help the policymakers understand a specific problem," a substantial proportion of respondents indicated that it is Very Important (66.7%) or Important (24.3%). Overall, the data suggests that there is a strong recognition of the significance of health research evidence in various aspects of policymaking and intervention implementation.

Table 6: Knowledge and skills of health research evidence use

Variable	VUIM n (%)	UIM n (%)	Neutral n (%)	IM n (%)	VIM n (%)
Gives the latest information	5(1.2)	3(1.2)	32(7.8)	100(24.51)	268(65.7)
Help the policymakers understand a specific problem	3(0.7)	4(1.0)	30(7.4)	99(24.3)	272(66.7)
Help the policymakers to implement various health interventions	3(0.7)	5(1.2)	24(5.9)	113(27.7)	263(64.5)
Avoid repeating the failures of others	3(0.7)	3(0.7)	19(4.7)	101(24.8)	282(69.1)
Introduces health planners to new ideas	3(0.7)	2(0.5)	19(4.7)	107(26.2)	277(67.9)

Table 7 provides information on the obstacles hindering the utilization of health research evidence. The variables include Lack of necessary knowledge and training in research,

Inadequate human and non-human resources, Difficulty in accessing health research evidence, Perceptions that health research is for academic purposes, Lack of involvement in research activities, and Traditional ways of planning, as well as the minimal usage of health research evidence among planning team members at the regional and council levels, and lack of dissemination.

The data is presented in terms of the number and percentage of participants who strongly disagree (SDSA), disagree (DSA), are neutral, agree (AG), and strongly agree (SAGR) with each obstacle. This data provides insight into the specific challenges that impact the utilization of health research evidence within the context of the study. The highest percentage is for "Perceptions that health research is for academic purposes" at 22.6%, and the lowest percentage is for "Lack of necessary knowledge and training in research" at 2.9%.

Table **7: Obstacles that hinder the utilization of health research evidence**

Variable	SDSA n (%)	DSA n (%)	Neutral n (%)	AG n (%)	SAGR n (%)
Lack of necessary knowledge and training in research	12(2.94)	18(4.4)	7(1.7)	67(16.4)	304(74.5)
Inadequate human and non-human resources	16(3.92)	18(4.4)	7(1.7)	81(19.9)	286(70.1)
Difficult in accessing health research evidence	36(8.82)	41(10.1)	24(5.9)	110(27.0)	197(48.3)
Perceptions that health research is for academic purposes	92(22.55)	40(9.8)	16(3.9)	81(19.9)	179(43.9)
Lack of involvement in research activities	15(3.68)	25(6.1)	11(2.7)	93(22.8)	264(64.7)
Traditional ways of planning, and Minimal usage of health research evidence among planning team members at the regional and council levels	36(8.82)	21(5.2)	13(3.2)	92(22.6)	246(60.3)
lack of dissemination	11(2.70)	15(3.7)	17(4.2)	105(25.7)	260(63.7)

Opportunities for the use of health research evidence

Table 8 indicates the opportunities for the use of health research evidence, helping stakeholders to identify areas for improvement and investment to optimize the use of health research evidence. The table presents the opportunities for utilizing health research evidence, categorized into physical and social opportunities.

For physical opportunities, the table highlights the availability of resources such as research coordinators, equipment, internet access, a planned budget for research, and the presence of symposiums for research discussions. The percentages indicate the distribution of responses, ranging from strongly disagree to strongly agree, reflecting the varying levels of agreement among the respondents regarding these physical opportunities. In the physical opportunities category, the highest percentage is 76.3%, representing the availability of the internet, while the lowest percentage is 0.7%, indicating the availability of Internet.

In the social opportunities section, the table emphasizes the presence of disparities among different populations, the involvement of community members in health planning teams, and the enhancement of health literacy in society. The percentages provide an insight into the attitudes and perceptions of the respondents toward these social opportunities. In the social opportunities category, the highest percentage is 55.9%, reflecting improved health literacy in society, and the lowest percentage is 2.4%, which represents the presence of disparities among different populations allowing for more equitable representation.

Table 8: Opportunities for the use of health research evidence

Variable	SDSA n (%)	DSA n (%)	Neutral n (%)	AG n (%)	SAGR n (%)
**Physical opportunities**					
Enabling the utilization of information generated from the healthcare system	6(1.4)	9(2.1)	18(4.3)	130(30.8)	259(61.4)
Availability of research coordinators	9(2.1)	18(4.3)	22(5.2)	93(22.0)	280(66.4)
Availability of equipment	4(1.0)	16(3.8)	21(5.0)	88(20.9)	293(69.4)

Availability of internet	3(0.7)	9(2.1)	19(4.5)	69(16.4)	322(76.3)
Availability of planned budget for research	11(2.6)	17(4.0)	27(6.4)	64(15.2)	303(71.8)
Presence of symposiums for research discussions	9(2.1)	17(4.0)	23(5.5)	106(25.1)	267(63.3)
**Social opportunities**					
The presence of disparities among different populations allows for more equitable	10(2.4)	24(5.7)	38(9.0)	147(34.8)	203(48.1)
The presence of community members in the health planning teams	11(2.6)	28(6.6)	55(13.0)	119(28.2)	209(49.5)
Improved health literacy in the society, empowering individuals to make informed	4(1.0)	19(4.5)	18(4.3)	145(34.4)	236(55.9)

Motivations for the use of health research evidence

Table 9, based on the data presented, we can observe that the automatic motivations for the use of health research evidence are primarily centered around the availability of transparency and accountability mechanisms 66.4% strongly agreeing, and 23.2% agreeing with the statement availability of job training 73.2% strongly agreeing and 21.3% agreeing respectively. Provision of incentives to health planners, with 72.4% strongly agreeing and 15.4% agreeing respectively.

In contrast, reflective motivations such as continuous quality improvement in the healthcare system and the presence of active stakeholder engagement in health planning also garnered significant support, with 63.51% and 59.48% strongly agreeing or agreeing, respectively. This indicates a strong emphasis on the proactive involvement of various stakeholders and the continuous enhancement of the healthcare system based on research evidence.

Table 9: Motivations of the use of health research evidence

Variable	SDSA n (%)	DSA n (%)	Neutral n (%)	AG n (%)	SAGR n (%)
**Automatic motivations**					

Provision of incentives to health planners	19(4.5)	21(5.0)	13(3.1)	65(15.4)	304(72.0)
Availability of job training	3(0.7)	4(1.0)	16(3.8)	90(21.3)	309(73.2)
Availability of Short-term courses	5(1.2)	13(3.1)	21(5.0)	75(17.8)	308(73.0)
Availability of Long-term course	18(4.3)	24(5.7)	24(5.7)	105(24.9)	251(59.5)
**Reflective motivations**					
Presence of interaction between policymakers, implementers, researchers, and academicians	3(0.7)	17(4.0)	21(5.0)	111(26.3)	270(64.0)
Presence of active stakeholder engagement in health planning	6(1.4)	16(3.8)	33(7.8)	116(27.5)	251(59.5)
Continuous quality improvement in the healthcare system	1(0.2)	10(2.4)	12(2.8)	131(31.0)	268(63.5)
Availability of transparency and accountability mechanisms	5(1.2)	12(2.8)	27(6.4)	98(23.2)	280(66.4)

Findings from the document review checklist

The analyzed data from Table 10 indicates that the majority of planning teams incorporate research evidence into their health plans. A substantial 97.8% of respondents confirmed having a current fiscal year health plan, and 98.9% reported having a planning guideline. Most plans include key health frameworks and guides, with 70.8% having the CCHP, 83.2% using the HF Planning Guide, and 89.9% incorporating situational analysis. Scientific engagement is evidenced by 75.3% of teams receiving invitation letters for conferences. However, challenges remain, as only 22.5% have a budget for research, and only 23.6% allocate a budget specifically for research within the health plan. Additionally, just 28.1% include a research coordinator in the planning team, and 43.8% of the plans are well-cited. This highlights a strong foundation in research usage but also gaps in financial and personnel support for research-driven planning.

Table 10: looking for evidence that planning teams use health research evidence in their plans

Variable	No	Yes	Not applicable
	n (%)	n (%)	n (%)

Do you have a current fiscal year health plan?	1(1.1)	87(97.8)	1(1.1)
Do you have a planning guideline?	1(1.12)	88(98.9)	0(0.0)
Presence of CCHP	12(13.5)	63(70.8)	14(15.7)
HF Planning Guide	13(14.6)	74(83.2)	2(2.3)
RHMT guide	26(29.2)	20(22.5)	43(48.3)
CHOP	30(33.7)	24(27.0)	35(39.3)
Presence of situational analysis in the plan	8(9.0)	80(89.9)	1(1.1)
Presence of invitation letters to attend various scientific conferences	22(24.7)	67(75.3)	0(0.0)
Are the well-cited plans	50(56.2)	39(43.8)	0(0.0)
Is there a budget for research available	69(77.5)	20(22.5)	0(0.0)
Is there a research-allocated budget in their health plan?	68(76.4)	21(23.6)	0(0.0)
Is the research coordinator among the planning team members who prepared the plan	64(71.9)	25(28.1)	0(0.0)

Discussion

This study represents the first comprehensive analysis of the use of health research evidence in Tanzania. It aimed to analyze the determinants of the use of health research evidence by deep-diving into the three determinants influencing behavior i.e. Capability, Opportunity, and Motivation. The findings from this study indicated that all three determinants significantly impact the ability of health planners to use health research evidence during health planning. However, we found that the use of research evidence is associated with having a degree, more than 10 years of involvement in health planning activities, and prior knowledge in research. We found several facilitator determinants such as organizational support infrastructures like, the availability of computers and the internet, the availability of research experts and research coordinators, availability of academic institutions that give a chance for dissemination of research. On the other hand, barrier determinants included lack of human resources, lack of

funding for research activities, lack of necessary knowledge and training, perceptions that research is for academic purposes, and lack of dissemination.

~~Proportion of the use of health research evidence~~

The findings of this study on the proportion of the use of health research evidence demonstrate a high level of reliance on health research evidence, particularly routine data, during health planning. The majority of participants (96.7%) reported using routine data, with nearly half (49.5%) using it extensively, which is consistent with findings from similar studies. For instance, a study by Lavis et al (23) found that routine data and health statistics are frequently utilized in policy-making and health-planning processes, underscoring the central role of such data in evidence-based decision-making. Moreover, the widespread use of health planning guidelines (98.8%) and the high proportion of participants (98.8%) recognizing the importance of health research evidence align with findings from studies on the use of research evidence in health decision-making process which emphasize the role of guidelines and evidence in shaping health policies (24,25). The high rate of health research evidence use (66.2%) in this study also mirrors the findings which reported similar levels of research evidence use in healthcare settings(26). These results highlight the growing recognition and integration of research evidence in health planning, reflecting broader trends in evidence-informed policymaking across health systems in Tanzania and globally.

~~Capability: The knowledge and skills of health research evidence use~~

Explicitly, capability determinants such as knowledge and skills were found to be critical, with health planning team members who had higher levels of education (Undergraduate, a master level, and above) and access to training more likely to use health research evidence to almost 70%, with the capability constraints faces more those with certificate and diploma level, lack of necessary knowledge and training in research accounts up to 70%, being the most barrier to utilization of health research evidence, inadequate human and non-human resources, difficult in accessing research evidence, perception that research is for academic purposes and lack of dissemination. These findings are consistent with systematic reviews conducted in other low- and middle-income countries, where similar capability constraints such as poor access to good quality relevant research, and lack of timely research output have been identified as major barriers to evidence-based health planning (27,28).

Formatted: Left, Indent: Left: -0.38", Space After: 8 pt, Line spacing: Multiple 1.08 li, Adjust space between Latin and Asian text, Adjust space between Asian text and numbers

~~Opportunities for the use of health research evidence~~

In comparing our results with existing literature on Opportunity determinants (both physical and social), it is evident that the availability of resources and institutional support plays a crucial role in enabling the use of research evidence. Our study revealed that health planning teams with better access to physical opportunities such as health information system, availability of research coordinators, equipment, internet, research budgets, presence of symposiums for research discussion, the existence of academic institutions such as universities, and social opportunities like the presence of community members representatives within the health planning teams and improved health literacy in the community were more inclined to utilize research findings in their work. This aligns with findings from two studies conducted in LMICs (29,30), which highlighted the importance of institutional support in facilitating the use of research evidence in health policy. However, our study also noted a distinct challenge in the Tanzanian context with limited access to relevant health research evidence due to infrastructural constraints, a barrier less emphasized in studies from more resource-rich settings (31,32).

~~Motivations for the use of health research evidence~~

Particularly the automatic motivations to utilize health research evidence were also found to be significant, especially on the provision of incentives to health planners to greater than 72%. Other motivations include job training, and short and long-term courses that influenced the motivations to use the health research evidence. The automatic motivation factors included the presence of interaction between policymakers, implementers, researchers, and academicians, and continuous quality improvement agenda in the health sector. Health planners who perceived the use of research evidence as beneficial and aligned with their professional goals were more likely to engage with it actively. This is in line with the COM-B model, which underscores the role of motivation in behavior change (10). Our findings add to the body of literature by highlighting the specific motivational barriers in Tanzania, such as a lack of a clear roadmap or framework for the utilization of health research evidence, which can reduce the enthusiasm for utilizing research in health planning (33)(34).

~~Document review checklist~~

The findings from the documents review checklist revealed a high level of integration of research evidence in health planning aligning with similar studies that underscore the importance of research-informed frameworks in public health planning (35)(36). The near-universal adoption of fiscal-year health plans (97.8%) and planning guidelines (98.9%) is consistent with global trends in evidence-based health planning, which emphasizes standardized planning processes (37). The presence of documents like CCHP and HF Planning guide mirrors findings from an urban strategic planning using blue-green infrastructure (38), who highlighted their role in improving health outcomes, the limited financial resources dedicated to health research in this study 22.5% suggests a common barrier. Similarly, the low inclusion of research coordinators (28.1%) echoes observations by a study designed for research approach during learning (39) on the limited interdisciplinary engagement in health planning. These gaps in budget allocation and personnel highlight persistent structural challenges to fully operationalizing research evidence-backed plans, despite a broad commitment to evidence-based frameworks.

The study provided valuable insights into the factors influencing the use of health research evidence during health planning in Tanzania. The results from the binary logistic regression analysis highlight significant associations between region, level of education, and years of participation in health planning. Notably, region was a significant predictor, with participants from regions other than Kilimanjaro demonstrating varying likelihoods of using research evidence, as indicated by the odds ratios. Educational attainment also emerged as a key determinant; individuals with undergraduate degrees were over twice as likely to use health research evidence compared to those with a diploma, with statistically significant results (p-value = 0.029). However, while master's degree holders showed a similar trend, the relationship was not statistically significant (p-value = 0.0747). The analysis of years of participation revealed that those with more than 10 years of experience were 40% more likely to use health research evidence, though this association was not statistically significant. These findings align with other studies that emphasize the importance of education and experience in evidence-based health planning (25,27). The adjusted odds ratios and confidence intervals provide a nuanced understanding of the strength and variability of these associations, contributing to a growing body of literature on the determinants of research utilization in public health policy and planning.

The implications of these findings are essential for improving health planning in Tanzania. To enhance the use of health research evidence, interventions must address all three determinants: enhancing the capability of health planners through capacity building, improving opportunities by ensuring better access to human and non-human resources and organizational support, and fostering motivation by recognizing and rewarding health planners that use evidence-based decision-making. These strategies align with recommendations from similar studies in other LMICs, suggesting that a multifaceted approach including the development of middle range theory (MRT) outlining the main facilitators for utilization of evidence for malaria treatment policy in Uganda and the importance of measuring total brain activity in neuroimaging are essential for promoting the use of research in health planning (40)(41).

Conclusion

This study provides a comprehensive assessment of the determinants influencing the use of health research evidence in Tanzania, focusing on capability, opportunity, and motivation. The findings demonstrate that all three determinants significantly impact the behavior of health planners, highlighting those factors such as higher education levels, years of experience in health planning, and prior research knowledge contribute to greater use of health research evidence. Organizational infrastructure and institutional support further facilitate this process, while barriers include insufficient human resources, lack of funding, inadequate training, and perceptions that research is solely for academic purposes.

The study also reveals a high reliance on routine data and health planning guidelines, indicating the growing recognition of research evidence in health decision-making. However, addressing the barriers to research evidence use will require targeted interventions that strengthen the capability of health planners, improve access to resources and institutional support, and enhance motivation through incentives and professional development.

Future research should explore the long-term impact of these interventions, as well as contextual factors like political will and leadership, to fully understand how these determinants collectively shape health planning behavior. A multifaceted approach i.e. a generic framework for strengthening Knowledge Management in Tanzania (KMT) that will have a triad pathway:

Capacity building on healthcare workers and researchers, infrastructure development, and funding that will improve access to health research database and resources within the healthcare system, and organizational support through the establishment of guidelines for the use of health research evidence in the decision-making process. This is essential for promoting the use of health research evidence, ultimately improving health outcomes in Tanzania and other low- and middle-income countries with similar challenges (42,43)

Abbreviations

CCHP	Comprehensive Council Health Planning
CHOP	Comprehensive Hospital Operational Plan
LGAs	Local Government Authorities
LMICs	Lower-and Middle-Income Countries
MoH	Ministry of Health
PO-RALG	President's Office-Regional Administration and Local Government
RHMT	Regional Health Management Team
SDGs	Sustainable Development Goals
UHC	Universal Health Coverage
WHO	World Health Organization

Acknowledgments

We would like to acknowledge the contribution of the University of Dodoma faculty members from the School of Nursing and Public Health for their input in the process of development of this manuscript. We would like to express our sincere thanks to the study facilities, the RHMTs, and the CHMTs for their support. We are grateful to the research assistants (Edmund Bunyaga, Theresia Ngungulu, Jestina Nyondo, Devina Mafuta Micky Masanyaji, and Ally Kinyaga) for their assistance during data collection.

Author Contributions

PK; Conceptualization, design of the work, data collection and analysis, preparing the manuscript draft, developing the methodology, results, discussion, and conclusion. RM, Conceptualization, critically reviewing the methodology and critically reviewing the manuscript, AK, Conceptualization, critically reviewing the methodology. All authors revised the work and approved the paper to be submitted for publication.

REFERENCES

1. Khalid FJI. The Impact of Poor Planning and Management on the Duration of Construction Projects. Multi-Knowl Electron Compr J Educ Sci Publ. 2019;(2):1–20.

2. Onwujekwe O, Etiaba E, Mbachu C, Arize I, Nwankwor C, Ezenwaka U, et al. Does improving the skills of researchers and decision-makers in health policy and systems research lead to enhanced evidence-based decision making in Nigeria?—A short term evaluation. Oelke N, editor. PLOS ONE. 2020 Sep 3;15(9):e0238365.

3. Henriksson DK, Peterson SS, Waiswa P, Fredriksson M. Decision-making in district health planning in Uganda: Does use of district-specific evidence matter? Health Res Policy Syst. 2019 Jun 6;17(1).

4. Kagoma P, Mongi R, Kapologwe NA, Kengia J, Kalolo A. Health research evidence: its current usage in health planning, determinants and readiness to use knowledge translation tools among health planning teams in Tanzania—an exploratory mixed-methods study protocol. BMJ Open. 2024 Jun;14(6):e081517.

5. Kengia JT, Kalolo A, Barash D, Chwa C, Hayirli TC, Kapologwe NA, et al. Research capacity, motivators and barriers to conducting research among healthcare providers in Tanzania's public health system: a mixed methods study. Hum Resour Health. 2023 Sep 5;21(1):73.

6. Van De Goor I, Hämäläinen RM, Syed A, Juel Lau C, Sandu P, Spitters H, et al. Determinants of evidence use in public health policy making: Results from a study across six EU countries. Health Policy. 2017 Mar;121(3):273–81.

7. Van Kammen J, Savigny DD, Sewankambo N. Using knowledge brokering to promote evidence-based policy-making: the need for support structures. Bull World Health Organ. 2006;84(8).

8. Guilbert JJ. The World Health Report 2006: Working together for health [1]. Educ Health Change Learn Pract. 2006;19(3):385–7.

9. Mboera LEG, Rumisha SF, Mbata D, Mremi IR, Lyimo EP, Joachim C. Data utilisation and factors influencing the performance of the health management information system in Tanzania. BMC Health Serv Res. 2021;21(1):4–11.

10. Michie S, van Stralen MM, West R. The behaviour change wheel: A new method for characterising and designing behaviour change interventions. Implement Sci. 2011 Apr 23;6(1).

11. De Leo A, Bayes S, Bloxsome D, Butt J. Exploring the usability of the COM-B model and Theoretical Domains Framework (TDF) to define the helpers of and hindrances to evidence-based practice in midwifery. Implement Sci Commun. 2021 Dec;2(1):7.

12. Kapologwe NA, Kalolo A, Kibusi SM, Chaula Z, Nswilla A, Teuscher T, et al. Understanding the implementation of Direct Health Facility Financing and its effect on health system performance in Tanzania: A non-controlled before and after mixed method study protocol. Health Res Policy Syst. 2019 Jan 30;17(1).

13. United THE, Of R. Comprehensive Council Health Planning Guideline Fifth Edition. 2019;

14. Ministry of Health. Guideline for Developing Comprehensive Hospital Operational Plan (CHOP) for Regional Referral Hospitals. 2016;(August).

15. Mwilike B, Nalwadda G, Kagawa M, Malima K, Mselle L, Horiuchi S. Knowledge of danger signs during pregnancy and subsequent healthcare seeking actions among women in Urban Tanzania: a cross-sectional study. BMC Pregnancy Childbirth. 2018 Dec;18(1):4.

16. Lyimo AA, Guo J, Mushy SE, Mwilike BE. Use of contraceptives and associated factors among male adolescents in rural secondary schools, Coast Region, Tanzania: a school-based cross-sectional study. Contracept Reprod Med. 2024 Mar 1;9(1):8.

17. Wahed T, Kaukab SST, Saha NC, Khan IA, Khanam F, Chowdhury F, et al. Knowledge of, attitudes toward, and preventive practices relating to cholera and oral cholera vaccine among urban high-risk groups: findings of a cross-sectional study in Dhaka, Bangladesh. BMC Public Health. 2013 Dec;13(1):242.

18. Subedi D. Explanatory sequential mixed method design as the third research community of knowledge claim. Am J Educ Res. 2016;4(7):570–7.

19. Taherdoost H. What Is the Best Response Scale for Survey and Questionnaire Design; Review of Different Lengths of Rating Scale / Attitude Scale / Likert Scale. 2019;8(1).

20. Shiyanbola OO, Rao D. Using an exploratory sequential mixed methods design to adapt an Illness Perception Questionnaire for African Americans with diabetes : the mixed data integration process. 2021;9(1):796–817.

21. Surucu L, Maslakci A. Validity and Reliability in Quantitative Research. Bus Manag Stud Int J. 2020;8(3):2694–726.

22. Jiyenze MK, Tundui C, Sirili N, Mollel H. Effects of implementing a health sector strategic plan : Eidence from Tanzania. East Afr Med J. 2022;99(9):5147–55.

23. Lavis J. Assessing country-level efforts to link research to action. Bull World Health Organ. 2006 Aug 1;84(8):620–8.

24. Hanney SR, Kanya L, Pokhrel S, Jones TH, Boaz A. How to strengthen a health research system: WHO's review, whose literature and who is providing leadership? Health Res Policy Syst. 2020 Dec;18(1):72.

25. Orton L, Lloyd-Williams F, Taylor-Robinson D, O'Flaherty M, Capewell S. The Use of Research Evidence in Public Health Decision Making Processes: Systematic Review. Ross JS, editor. PLoS ONE. 2011 Jul 26;6(7):e21704.

26. Innvær S, Vist G, Trommald M, Oxman A. Health policy-makers' perceptions of their use of evidence: a systematic review. J Health Serv Res Policy. 2002 Oct 1;7(4):239–44.

27. Oliver K, Innvar S, Lorenc T, Woodman J, Thomas J. A systematic review of barriers to and facilitators of the use of evidence by policymakers. BMC Health Serv Res. 2014 Dec;14(1):2.

28. Koon AD. A scoping review of the uses and institutionalisation of knowledge for health policy in low- and middle-income countries. 2020;

29. Lavis JN, Paulsen EJ, Oxman AD, Moynihan R. Evidence-informed health policy 2 – Survey of organizations that support the use of research evidence. Implement Sci. 2008;3(1):1–17.

30. Lavis JN, Wilson MG, Grimshaw JM, Haynes RB, Hanna S, Raina P, et al. Effects of an evidence service on health-system policy makers' use of research evidence: A protocol for a randomised controlled trial. 2011;

31. Moat KA, Lavis JN. 10 best resources for . . . evidence-informed health policy making.

32. Gavine A. Maximising the availability and use of high-quality evidence for policymaking: collaborative, targeted and efficient evidence reviews. PALGRAVE Commun. 2018;

33. Armstrong R, Waters E, Dobbins M, Anderson L, Moore L, Petticrew M, et al. Knowledge translation strategies to improve the use of evidence in public health decision making in local government: intervention design and implementation plan. Implement Sci. 2013 Dec;8(1):121.

34. Armstrong JS. Guidelines for Science: Evidence and Checklists.

35. Smith JD, Hasan M. Quantitative approaches for the evaluation of implementation research studies. Psychiatry Res. 2020 Jan;283:112521.

36. Roberts-Lewis SF, Baxter HA, Mein G, Quirke-McFarlane S, Leggat FJ, Garner HM, et al. The Use of Social Media for Dissemination of Research Evidence to Health and Social Care Practitioners: Protocol for a Systematic Review. JMIR Res Protoc. 2023 May 12;12:e45684.

37. Brown G, Kyttä M, Reed P. Using community surveys with participatory mapping to monitor comprehensive plan implementation. Landsc Urban Plan. 2022 Feb;218:104306.

38. Sörensen J, Persson AS, Olsson JA. A data management framework for strategic urban planning using blue-green infrastructure. J Environ Manage. 2021 Dec;299:113658.

39. Lim FV, Nguyen TTH. Design-based research approach for teacher learning: a case study from Singapore. ELT J. 2022 Oct 14;76(4):452–64.

40. Nabyonga-Orem J, Ssengooba F, Macq J, Criel B. Malaria treatment policy change in Uganda: what role did evidence play? 2014;

41. Hyder F, Rothman DL. Evidence for the importance of measuring total brain activity in neuroimaging.

42. Bennett NJ. Using perceptions as evidence to improve conservation and environmental management. Conserv Biol. 2016;30(3).

43. Hollingworth SA. What do we need to know? Data sources to support evidence-based decisions using health technology assessment in Ghana. . Epidemiology. 2020;

Supporting information captions

S1 Dataset used in the study

Formatted: Indent: Left: 0.01", No bullets or numbering

Analyzing the determinants for using health research evidence in health planning in Tanzania: a cross-sectional study

Response to reviewer's comments

We would like to thank the reviewer for the comments provided to improve this study. We have revised the manuscript as shown in the point-to-point responses to the reviewer's comments provided in the table matrix below.

Comments	Authors Response
**Journal requirements:**	
1. Please ensure that your manuscript meets PLOS ONE's style requirements, including those for file naming. The PLOS ONE style templates can be found at https://journals.plos.org/plosone/s/file?id=wjVg/PLOSOne_formatting_sample_main_body.pdf and https://journals.plos.org/plosone/s/file?id=ba62/PLOSOne_formatting_sample_title_authors_affiliations.pdf	Thank you for your comment. We have read PLOS ONE's style requirement and have used it to make revisions including file naming for our dataset
2. Please provide additional details regarding participant consent. In the ethics statement in the Methods and online submission information, please ensure that you have specified (1) whether consent was informed and (2) what type you obtained (for instance, written or verbal, and if verbal, how it was documented and witnessed). If your study included minors, state whether you obtained consent from parents or guardians. If the need for consent was waived by the ethics committee, please include this information. If you are reporting a retrospective study of medical records or archived samples, please ensure that you have discussed whether all data were fully anonymized before you accessed them and/or whether the IRB or ethics committee waived the requirement for informed consent. If patients provided informed written consent to have data from their medical records used in research, please include this information.	Thank you for the comment. We confirm that informed written consent was obtained from all participants, details have been clarified in the Ethics Statement of the manuscript.

3. When completing the data availability statement of the submission form, you indicated that you will make your data available on acceptance. We strongly recommend all authors decide on a data sharing plan before acceptance, as the process can be lengthy and hold up publication timelines. Please note that, though access restrictions are acceptable now, your entire data will need to be made freely accessible if your manuscript is accepted for publication. This policy applies to all data except where public deposition would breach compliance with the protocol approved by your research ethics board. If you are unable to adhere to our open data policy, please kindly revise your statement to explain your reasoning and we will seek the editor's input on an exemption. Please be assured that, once you have provided your new statement, the assessment of your exemption will not hold up the peer review process.	Thank you for your guidance. We confirm that we will make the data fully available before acceptance ensuring compliance with our research ethics board, we have clarified this in the Data Availability Statement and are open to further guidance from the editor.
4. Please amend either the abstract on the online submission form (via Edit Submission) or the abstract in the manuscript so that they are identical.	Thank you for your feedback. We have carefully reviewed both the abstract in the online submission form and the abstract in the manuscript to ensure they are identical.
5. We note that Figure 2 in your submission contain [map/satellite] images which may be copyrighted. All PLOS content is published under the Creative Commons Attribution License (CC BY 4.0), which means that the manuscript, images, and Supporting Information files will be freely available online, and any third party is permitted to access, download, copy, distribute, and use these materials in any way, even commercially, with proper attribution. For these reasons, we cannot publish previously copyrighted maps or satellite images created using proprietary data, such as Google software (Google Maps, Street View, and Earth). For more information, see our copyright guidelines: http://journals.plos.org/plosone/s/licenses-and-copyright.<br>We require you to either (1) present written permission from the copyright holder to publish these figures specifically under the CC BY 4.0	Thank you for your feedback. We have reviewed Figure 2 and acknowledge the copyright requirements. To comply with the CC BY 4.0 license, we have removed the figure as required.

license, or (2) remove the figures from your submission:<br>1. You may seek permission from the original copyright holder of Figure 2 to publish the content specifically under the CC BY 4.0 license.<br>We recommend that you contact the original copyright holder with the Content Permission Form (http://journals.plos.org/plosone/s/file?id=7c09/content-permission-form.pdf) and the following text:<br>“I request permission for the open-access journal PLOS ONE to publish XXX under the Creative Commons Attribution License (CCAL) CC BY 4.0 (http://creativecommons.org/licenses/by/4.0/). Please be aware that this license allows unrestricted use and distribution, even commercially, by third parties. Please reply and provide explicit written permission to publish XXX under a CC BY license and complete the attached form.”<br>Please upload the completed Content Permission Form or other proof of granted permissions as an "Other" file with your submission.<br>In the figure caption of the copyrighted figure, please include the following text: “Reprinted from [ref] under a CC BY license, with permission from [name of publisher], original copyright [original copyright year].”<br>2. If you are unable to obtain permission from the original copyright holder to publish these figures under the CC BY 4.0 license or if the copyright holder’s requirements are incompatible with the CC BY 4.0 license, please either i) remove the figure or ii) supply a replacement figure that complies with the CC BY 4.0 license. Please check copyright information on all replacement figures and update the figure caption with source information. If applicable, please specify in the figure caption text when a figure is similar but not identical to the original image and is therefore for illustrative purposes only.<br>The following resources for replacing copyrighted map figures may be helpful:<br>USGS National Map Viewer (public domain): http://viewer.nationalmap.gov/viewer/	

The Gateway to Astronaut Photography of Earth (public domain): http://eol.jsc.nasa.gov/sseop/clickmap/<br>Maps at the CIA (public domain): https://www.cia.gov/library/publications/the-world-factbook/index.html and https://www.cia.gov/library/publications/cia-maps-publications/index.html<br>NASA Earth Observatory (public domain): http://earthobservatory.nasa.gov/<br>Landsat: http://landsat.visibleearth.nasa.gov/<br>USGS EROS (Earth Resources Observatory and Science (EROS) Center) (public domain): http://eros.usgs.gov/#<br>Natural Earth (public domain): http://www.naturalearthdata.com/	
6. Please include captions for your Supporting Information files at the end of your manuscript, and update any in-text citations to match accordingly. Please see our Supporting Information guidelines for more information: http://journals.plos.org/plosone/s/supporting-information.	Thank you for your feedback. We have added captions for the Supporting Information files at the end of the manuscript and updated the in-text citations accordingly.
**Additional Editor Comments:**	
In the methodology section, you have narrated the scoring tools (likert scales and questions), but no source was cited. Please insert the citation for the source(s) in the text with their corresponding references in the reference list.	Thank you for your nice comment. We have added the appropriate citations for the scoring tools (Likert scales and questions) in the methodology section and included the corresponding references in the reference list.
Please read critically the comments raised by the reviewer and address each comment accordingly.	
**Reviewer #1**	
Specific comments:<br>1. Methodology-The methodology is not clear. For example, there is no specific subsection to describe the characteristics of the study participants. Also, calculation of the sample size was wrongly done by considering marginal error	Thank you for your comment. We have revised the methodology section to clearly define the study participants' characteristics and corrected the sample

of 50% which does not exist.<br>-Definition or measurement of the dependent variable which is use of health research evidence is not clear. Authors stated that four questions were used for assessing the use of health research evidence without stating the source of this approach. This affects the authenticity and also reproducibility of the results from the current study.	size calculation by using an appropriate margin of error. The previously used 50% was an assumed proportion; however, there was an error in how it was presented, which we have now rectified for clarity. Additionally, we have provided a more explicit description of how the use of health research evidence was assessed, including details on the measurement of the dependent variable. To enhance the authenticity and reproducibility of our study, we have also included citations for the questions used in the assessment.
Results: Are not precise and lack a clear message to the readers.	Thank you for your valuable feedback. We appreciate your insights and have carefully revised the results section to enhance clarity and ensure a precise and coherent message for the readers. We have also refined our language and structure to improve readability and effectively communicate our key findings.
**Reviewer #2:**	
I have read the manuscript and I found it good and I do not have any significant comments regarding it. Introduction is good, methodology was described very well. Results also were clear. Discussion and conclusion were good as well	Thank you so much for your thoughtful review and kind words. We truly appreciate your time and effort in reviewing our manuscript. Your constructive feedback encourages us, and we are grateful that you found the work

	well-structured and clear. Thank you again for your support.
