# Supplementary material for: Analysis of the determinants for using health research evidence in health planning in Tanzania: a cross-sectional study
Source: PLoS One. 2025 May 28;20(5):e0316508. doi: 10.1371/journal.pone.0316508 (PMC12118992; doi:10.1371/journal.pone.0316508)
Supplement: Attachment — Submitted filename: PONE-D-24-MANUSCRIP WITH REVIEW COMMENTS.docx [file pone.0316508.s002.docx]

**PLOS ONE**

## Analyzing the determinants for using health research evidence in health planning in Tanzania: a cross-sectional study.

--Manuscript Draft--

| **Manuscript Number:** | PONE-D-24-56677 |
| --- | --- |
| **Article Type:** | Research Article |
| **Full Title:** | Analyzing the determinants for using health research evidence in health planning in Tanzania: a cross-sectional study. |
| **Short Title:** | The use of health research evidence during health planning |
| **Corresponding Author:** | Pius Kagoma, MD, MPH  University of Dodoma College of Health and Allied Sciences: The University of Dodoma College of Health Sciences  DODOMA, TANZANIA, UNITED REPUBLIC OF |
| **Keywords:** | Determinants; Research evidence use; Health planning; Tanzania |
| **Abstract:** | Introduction  Achieving Universal Health Coverage (UHC) requires utilizing research evidence to inform the decision-making process. However, little information is available on the determinants for using research evidence in planning in Lower Middle-Income Countries (LMICs), including Tanzania. This paper aims to investigate the determinants of using health research evidence in health planning in Tanzania.  Materials and methods  This study employed a cross-sectional study design. Data on the use of health research evidence and its determinants were collected using a structured questionnaire from 422 respondents from 9 regions of Tanzania from April to May 2024. The data were analyzed using STATA version 18 for descriptive and inferential statistics. The association between variables was determined using a chi-square test at a 95% confidence level.  Results  The study revealed that 66.2% of participants strongly agreed to use health research evidence during planning. However, significant barriers were identified, including lack of dissemination (74.5%), inadequate human and non-human resources (70.0%), and insufficient knowledge and training in research (63.7%). A chi-square test confirmed significant associations between these barriers and the reduced use of research evidence (p<0.05). Conversely, more than 70% of respondents identified opportunities such as the availability of research coordinators, university partnerships, available research budgets, and internet access, all significantly associated with increased health research evidence use. More than 50% of participants reported motivational factors that like continuous quality improvement agenda in the healthcare sector, availability of short and long-term courses, on-the-job training, and provision of incentives like extra duty allowances were positively linked to research utilization. |
| **Order of Authors:** | Pius Kagoma, MD, MPH |
|  | Richard Mongi, PhD |
|  | Albino Kalolo, MD, PhD |
| **Additional Information:** |  |
| **Question** | **Response** |
| **Financial Disclosure**  Enter a financial disclosure statement that | The author(s) received no specific funding for this work. |

| describes the sources of funding for the work included in this submission. Review the [submission guidelines](http://journals.plos.org/plosone/s/submission-guidelines#loc-financial-disclosure-statement) for detailed requirements. View published research articles from [*PLOS ONE*](https://journals.plos.org/plosone/) for specific examples.  This statement is required for submission and **will appear in the published article** if the submission is accepted. Please make sure it is accurate. |  |
| --- | --- |
| **Funded studies**  Enter a statement with the following details:   - Initials of the authors who received each award - Grant numbers awarded to each author - The full name of each funder - URL of each funder website - Did the sponsors or funders play any role in the study design, data collection and analysis, decision to publish, or preparation of the manuscript? |  |
| Did you receive funding for this work? |  |
| **Competing Interests**  Use the instructions below to enter a competing interest statement for this submission. On behalf of all authors, disclose any [competing interests](http://journals.plos.org/plosone/s/competing-interests) that could be perceived to bias this work—acknowledging all financial support and any other relevant financial or non- financial competing interests.  This statement is required for submission and **will appear in the published article** if the submission is accepted. Please make sure it is accurate and that any funding sources listed in your Funding Information later in the submission form are also declared in your Financial Disclosure statement.  View published research articles from  [*PLOS ONE*](http://journals.plos.org/plosone/) for specific examples. | The authors have declared that no competing interests exist. |

| **NO authors have competing interests**  Enter: *The authors have declared that no competing interests exist.*  **Authors with competing interests**  Enter competing interest details beginning with this statement:  *I have read the journal's policy and the authors of this manuscript have the following competing interests: [insert competing interests here]* |  |
| --- | --- |
| * typeset |  |
| **Ethics Statement**  Enter an ethics statement for this submission. This statement is required if the study involved:   - Human participants - Human specimens or tissue - Vertebrate animals or cephalopods - Vertebrate embryos or tissues - Field research   Write "N/A" if the submission does not require an ethics statement.  General guidance is provided below. Consult the [submission guidelines](http://journals.plos.org/plosone/s/submission-guidelines#loc-guidelines-for-specific-study-types) for detailed instructions. **Make sure that all information entered here is included in the Methods section of the manuscript.** | The study was granted ethical approval by the University of Dodoma Ethical Committee with Ref. No. MA.84/261/102/’A’/64/91. Permission to conduct the study and consent to participate in the study was sought from relevant authorities and participants, respectively. Participants received information about the purpose of the study and data protection. |

| **Format for specific study types**  **Human Subject Research (involving human participants and/or tissue)**   - Give the name of the institutional review board or ethics committee that approved the study - Include the approval number and/or a statement indicating approval of this research - Indicate the form of consent obtained (written/oral) or the reason that consent was not obtained (e.g. the data were analyzed anonymously)   **Animal Research (involving vertebrate animals, embryos or tissues)**   - Provide the name of the Institutional Animal Care and Use Committee (IACUC) or other relevant ethics board that reviewed the study protocol, and indicate whether they approved this research or granted a formal waiver of ethical approval - Include an approval number if one was obtained - If the study involved *non-human primates*, add *additional details* about animal welfare and steps taken to ameliorate suffering - If anesthesia, euthanasia, or any kind of animal sacrifice is part of the study, include briefly which substances and/or methods were applied   **Field Research**  Include the following details if this study involves the collection of plant, animal, or other materials from a natural setting:   - Field permit number - Name of the institution or relevant body that granted permission |  |
| --- | --- |
|  |  |
| **Data Availability**  Authors are required to make all data underlying the findings described fully available, without restriction, and from the time of publication. PLOS allows rare exceptions to address legal and ethical concerns. See the [PLOS Data Policy](http://journals.plos.org/plosone/s/data-availability) and [FAQ](http://journals.plos.org/plosone/s/data-availability#loc-faqs-for-data-policy) for detailed information. | Yes - all data are fully available without restriction |

| A Data Availability Statement describing where the data can be found is required at submission. Your answers to this question constitute the Data Availability Statement and **will be published in the article**, if accepted. |  |
| --- | --- |
| **Important:** Stating ‘data available on request from the author’ is not sufficient. If your data are only available upon request, select ‘No’ for the first question and explain your exceptional situation in the text box. |  |
| Do the authors confirm that all data underlying the findings described in their manuscript are fully available without restriction? |  |
| **Describe where the data may be found in full sentences. If you are copying our sample text, replace any instances of XXX with the appropriate details.**   - If the data are **held or will be held in a public repository**, include URLs, accession numbers or DOIs. If this information will only be available after acceptance, indicate this by ticking the box below. For example: *All XXX files are available from the XXX database (accession number(s) XXX, XXX.*). - If the data are all contained **within the manuscript and/or Supporting Information files**, enter the following: *All relevant data are within the manuscript and its Supporting Information files.* - If neither of these applies but you are able to provide **details of access elsewhere**, with or without limitations, please do so. For example:   *Data cannot be shared publicly because of [XXX]. Data are available from the XXX Institutional Data Access / Ethics Committee (contact via XXX) for researchers who meet the criteria for access to confidential data.*  *The data underlying the results presented in the study are available from (include the name of the third party* | All data is available in the manuscript and the repository |

| *and contact information or URL).*   - This text is appropriate if the data are owned by a third party and authors do not have permission to share the data.   * typeset |  |
| --- | --- |
| Additional data availability information: | Tick here if the URLs/accession numbers/DOIs will be available only after acceptance of the manuscript for publication so that we can ensure their inclusion before publication. |

Manuscript	[Click here to access/download;Manuscript;Main document.docx](https://www2.cloud.editorialmanager.com/pone/download.aspx?id=38605153&guid=5a516d15-ce95-46b3-8811-1dffe5dccb6c&scheme=1)[<https://www2.cloud.editorialmanager.com/pone/download.aspx?id=38605153&guid=5a516d15-ce95-46b3-8811-1dffe5dccb6c&scheme=1>](https://www2.cloud.editorialmanager.com/pone/download.aspx?id=38605153&guid=5a516d15-ce95-46b3-8811-1dffe5dccb6c&scheme=1)

1 ***Analyzing the determinants for using health research evidence***
2 ***in health planning in Tanzania: a cross-sectional study.***
3 Pius Kagoma*^1&2,^ Richard Mongi^1^, Albino Kalolo^3,^ ^4^
4 *^1^Department of Public Health and Community Nursing, the University of Dodoma, Dodoma,*
5 *Tanzania;*
6 *^2^Division of Health, Social Welfare and Nutrition Services, President’s Office-Regional*
7 *Administration Local Government, Dodoma, Tanzania;*
8 *^3^Department of Public Health, St. Francis University College of Health and Allied Sciences,*
9 *Morogoro, Tanzania;*
10 *^4^Centre	for	Reforms,	Innovation,	Health	Policies,	and	Implementation	Research,*
11 *Dodoma,Tanzania.*
12 *Corresponding author: Pius Kagoma; +255 (0) 76 5909942; P.O. Box 1923 Dodoma, Tanzania;
13 [piuskagoma@gmail.com](mailto:piuskagoma@gmail.com)
14 Richard	Mongi;	+255	(0)	67	6953510;	P.O.	Box	259,	Dodoma,	Tanzania;
15 [richiemongi@gmail.com](mailto:richiemongi@gmail.com)
16 Albino	Kalolo;	+255	(0)	764340819;	P.O.	Box	175,	Ifakara,	Morogoro,	Tanzania;
17 [kaloloa@gmail.com](mailto:kaloloa@gmail.com)

# 18 Abstract

19 **Introduction:** Achieving Universal Health Coverage (UHC) requires utilizing research
20 evidence to inform the decision-making process. However, little information is available on the
21 determinants for using research evidence in planning in Lower Middle-Income Countries
22 (LMICs), including Tanzania. This paper aims to investigate the determinants of using health
23 research evidence in health planning in Tanzania.

24

25 **Materials and methods:** This study employed a cross-sectional study design. Data on health
26 research evidence and its determinants were collected using a structured questionnaire from 422
27 respondents from 9 regions of Tanzania from October to December 2023. The data were
28 analyzed using STATA version 18 for descriptive and inferential statistics. The association
29 between variables was determined using a chi-square test at a 95% confidence level.
30 **Results:** The study revealed that 66.2% of participants strongly agreed to use health research
31 evidence during planning. However, significant barriers were identified, including lack of
32 dissemination (74.5%), inadequate human and non-human resources (70.0%), and insufficient
33 knowledge and training in research (63.7%). A chi-square test confirmed significant associations
34 between these barriers and the reduced use of research evidence (p<0.05). Conversely, more than
35 70% of respondents identified opportunities such as the availability of research coordinators,
36 university partnerships, available research budgets, and internet access, all significantly
37 associated with increased health research evidence use. More than 50% of participants reported
38 motivational factors that like continuous quality improvement agenda in the healthcare sector,
39 availability of short and long-term courses, on-the-job training, and provision of incentives like
40 extra duty allowances were positively linked to research utilization.
41 **Conclusion:** The study found that 66.2% of participants used health research evidence in
42 planning, but barriers like lack of dissemination, resource shortages, and inadequate training
43 persisted. Interventions should focus on improving dissemination, resources, and training. Future
44 research should explore strategies for enhancing these interventions.
45 **Keywords:** Determinants, Research evidence use, Health planning, Tanzania.

46

47

# 48 INTRODUCTION

49 Health research evidence plays an essential role in shaping effective health policies and
50 interventions, which are crucial for improving public health outcomes (1). In resource-limited
51 settings like Tanzania, where health challenges are complex and resources are constrained, the
52 strategic use of research evidence is particularly important. It can guide decision-makers in
53 identifying priority areas, optimizing resource allocation, and implementing interventions that
54 are both cost-effective and impactful. Moreover, the use of evidence can help health planning
55 teams avoid repeating past failures and introduce innovative solutions (2). Despite its recognized
56 importance, the extent to which health research evidence is utilized by health planning teams in
57 Tanzania remains unclear. Understanding the factors that influence this utilization is essential for
58 promoting evidence-based decision-making, which is critical for achieving the ambitious health
59 targets set forth in the Sustainable Development Goals (SDGs), particularly Universal Health
60 Coverage (UHC)(3).
61 Efforts to promote the integration of research evidence into health planning and decision-making
62 have been made in various settings. These include the establishment of knowledge translation
63 platforms, research dissemination strategies, health conferences, and capacity-building initiatives
64 targeting health planning teams. However, the success of these efforts varies significantly across
65 contexts, largely depending on factors such as institutional support, the capacity of the health
66 workforce, and access to reliable data. In Tanzania, while some initiatives aim to bridge the gap
67 between research and practice, the actual impact of these efforts on health planning remains
68 under-explored.
69 Research across different settings has demonstrated that the use of health research evidence in
70 decision-making is influenced by a variety of factors. These include organizational culture, the
71 availability of resources, the expertise of the planning teams, and access to research outputs [4].
72 In a study conducted by Kagoma et al (4), it was found that health planning teams in Tanzania
73 consist of professionals from various disciplines, including Administrators (9%), Medical
74 doctors (25.36%), Nurses (23.46%), Laboratory scientists (8.53%), Pharmacists (6.64%),
75 Radiographers (1.66%), Environmental health officers (3.08%), Nutrition officers (3.08%),
76 Social welfare officers (4.74%), and others (14.46%). Their primary responsibility is to plan,
77 implement, and evaluate health interventions [4]. However, several factors may limit their ability
78 to fully integrate research evidence into their work (5). These include inadequate dissemination
79 of research findings, lack of access to research evidence, limited skills in interpreting research
80 data, and insufficient organizational support (3,6–8).
81 While previous studies have explored various factors influencing the use of health research
82 evidence in policy and planning, there remains a gap in understanding the specific determinants
83 that affect the utilization of health research evidence among health planning teams in
84 Tanzania(4,9). This study seeks to fill this gap by analyzing the key factors that influence
85 evidence use among these teams at both the regional and council levels, guided by the COM-B
86 Model (Capability, Opportunity, and Motivation) as the theoretical framework (10), which has
87 been widely used in understanding evidence-based decision-making processes. The COM-B
88 model is particularly suited to the Tanzanian context because it focuses on the essential
89 components of behavior change Capability, Opportunity, and Motivation which are critical for
90 understanding and addressing the factors influencing the use of health research evidence by
91 planning teams. By examining these elements, the model helps identify specific barriers and
92 enablers that can be targeted to improve evidence-based decision-making in Tanzania's resource-
93 limited. By applying this theoretical lens, the study aims to provide a deeper understanding of the
94 barriers and facilitators to using research evidence in health planning, with the following specific
95 objectives: 1) Analyze the current usage of health research evidence among planning team
96 members at the regional and council levels, 2) Analyze the capacity of health planning team
97 members to utilize health research evidence, 3) Identify opportunities for enhancing the use of
98 health research evidence in health planning, 4) Identify opportunities for enhancing the use of
99 health research evidence in health planning, 5) Explore the motivations for using health research
100 evidence among health planning members at both regional and council levels. The findings of
101 this study are expected to contribute to the development of strategies that enhance the integration
102 of research evidence into health planning processes, ultimately leading to more effective health
103 interventions and improved health outcomes in Tanzania.

## 104 Conceptual framework

105 The conceptual framework for this study was developed after several consultative meetings with
106 different stakeholders and researchers. The theory was adopted and modified from the
107 (Capability, Opportunity, and Motivation) COM-B model. The COM- B Model at the center of a
108 proposed framework is a behavior system involving three essential conditions: Capability,
109 Opportunity, and Motivation, what we term the ‘COM-B system(10,11). This study investigated
110 how the capability, opportunity, and motivation determinants can influence the behavior of the
111 use of health research evidence during health planning as shown in Figure 1

112

| 113 |  |
| --- | --- |
| 114 | ………………………………………………………………… |
| 115 | Insert figure 1 |
| 116 | …………………………………………………………………. |
| 117 | **Figure 1**: The domains of the COM-B Model modified from Michie et al,.2011(10) |
| 118 | **Materials and Methods** |
| 119 | **Study setting** |
| 120 | This study was conducted between October and December 2023 in the United Republic of |
| 121 | Tanzania, a Lower and Middle low-income country located in East Africa, with a population of |
| 122 | about 62 million people. The allocated budget for the country’s Ministry of Health in 2023/2024 |
| 123 | was estimated to be 443.6 million US dollars, with 1.2 million US dollars (0.27%) allocated for |
| 124 | evidence production. The health research evidence users in Tanzania involve three important |
| 125 | Ministries, namely; the Ministry of Health (MoH), The President’s Office Regional |
| 126 | Administration and Local Government (PO-RALG), and Health management. The health |
| 127 | evidence producers in Tanzania are the National Institute for Medical Research (NIMR), the |
| 128 | Tanzania Commission for Science and Technology (COSTECH), Public and Private |
| 129 | Universities, Health related institutions or authorities, local and international non-governmental |
| 130 | organizations (NGOs), and civil society organizations as shown in Table 1. |
| 131  132 | **Table 1: A list of health research evidence producers and users in Tanzania** |

133

134

| **SN** | **Evidence users** |
| --- | --- |
| 1 | Ministry of Health |
| 2 | President’s Office Regional Administration and Local Government |
| 3 | Ministry of Education Science and Technology (MEST) |
| 4 | Prime Minister’s Office |
| 5 | Regional Health Management Teams |

| 6 | Council Health Management Teams |
| --- | --- |
| 7 | Healthcare workers |
| 8 | Non-Governmental Organizations (NGOs) |
| 9 | Civil Society Organizations (CSOs) |
| 10 | Private Sector |
| **Evidence producers** | |
| 1 | National Institute for Medical Research (NIMR) |
| 2 | Tanzania Commission for Science and Technology (COSTECH) |
| 3 | Public Universities; The Muhimbili University of Health and Allied Sciences  (MUHAS), University of Dar es Salaam (UDSM), University of Dodoma (UDOM) |
| 4 | Private Universities; Catholic University of Health and Allied Sciences (CUHAS), Kilimanjaro Christian Medical Centre (KCMC), Hubert Kairuki Memorial University (HKMU), St. John’s University of Tanzania (SJUT), St. Augustine University of Tanzania (SAUT), Kampala International University (KIU), St. Francis University College of Health and Allied Sciences  (SFUCHAS) |
| 5 | Kilimanjaro Clinical Research Institute (KCRI) |
| 6 | Ifakara Health Institute (IHI) |
| 7 | Non-Governmental Organizations (NGOs) |
| 8 | Civil society organizations (CSOs) |
| 9 | Private sector |

135

136

## 137 The study sites

138 The study site was eighteen (18) Councils of the Local Government Authorities within the nine
139 (9) regions out of 26 Regions of Tanzania Mainland from nine (9) geographical zones as
140 summarized in Table 2 and Figure 2. The nine zones are selected to seek the country’s
141 geographical representation. Together, these regions have a total population of 21,119,700,
142 which represents 35.7% of the Tanzanian population. These regions are heterogeneous in
143 population size, distribution of health facilities, human resources for health, and institutions
144 carrying out health research activities. A similar approach has been used in major Tanzanian
145 health studies(4,5,12); This approach provided a comprehensive understanding of the status quo
146 for the use of health research evidence in health planning, and heterogeneous study population
147 for random sampling hence acting as a representative snapshot of all regions in Tanzania. The
148 study was conducted in 63 randomly selected health facilities, nine (9) Regional Referral
149 Hospitals (RRHs), eighteen (18) District Hospitals (DHs), Eighteen (18) Health Centres (HCs),
150 and eighteen (18) Dispensaries. To ensure the inclusion of urban and rural health facilities,
151 stratification was conducted followed by random sampling.

152

153

154

155

156

157	**Table 2**: Summary of study sites

| **ZONE** | **REGION** | **COUNCIL** |
| --- | --- | --- |
| Northern |  | Siha District |
|  | Kilimanjaro | Moshi Municipal |
| Central zone |  | Chamwino District |
|  | Dodoma | Dodoma City |
| Dar es Salaam |  | Kigamboni Municipal |
|  | Dar es Salaam | Dar es Salaam City |
| Eastern |  | Morogoro Municipal |
|  | Mororgoro | Gairo District |
| Western |  | Kigoma District |
|  | Kigoma | Kigoma Municipal |
| Southwest highland |  | Mbeya City |
|  | Mbeya | Kyela District |
| Lake |  | Kwimba District |
|  | Mwanza | Mwanza City |
| Southern |  | Mtwara Municipal |
|  | Mtwara | Nanyamba |
| Southern Highland | Iringa | Iringa Municipal |
|  |  | Iringa District |

158

159

160 …………………………………………………………………
161 Insert figure 2
162 …………………………………………………………………….
163 **Figure 2**: Distribution of sampled facilities by zones, regions, and districts

164

## 165 The health planning landscape in Tanzania

166 The health planning process in Tanzania is conducted at two levels; At the Council level, the
167 health facility prepares its plans as well and the Council Health Management Team (CHMT)
168 prepares its plan, whereby plans from the facility and CHMT are later consolidated to form
169 Comprehensive Council plan (CCHP). The preparation of health facility plans is guided by the
170 Health facility planning guidelines and CCHP guidelines.(13). At the regional level, the Regional
171 Health Management Teams (RHMTs) prepare their plans using the RHMT planning guide and the
172 Regional Referral Hospitals (RRHs) plans using the Comprehensive Operational Plan Guide
173 (CHOP)(14). When the plans are completed are sent to the Ministry of Health and President’s
174 Office-Regional Administration and Local Government for final assessment before being sent to
175 the Ministry of Finance for funding. At all levels, we are planning to use the guidelines and the
176 routine data collected from each level.(14).

## 177 Study Design

178 The study adopts a quantitative cross-sectional design focusing on nine regions of Tanzania.
179 (15,16).

## 180 Sample size and Sampling procedures

181 The sample size involved the health planning team members from the RRHs, DHs, HCs, and
182 Dispensaries randomly selected from the nine regions. The sample size was 422 calculated from
183 the Cochrane formula (1977). To date, in Tanzania there is no cited reference for the percentage
184 of the use of health research evidence in health planning therefore the marginal error of 50% was
185 used (17).

186

187

188 Where by
189 N-sample size
190 Z-confidence	interval	95%	1.96,	P-
191 proportional from previous study d = margin
192 of error, which is approximately 5% Thus,

| 193 | **n= (1.96)^2^ x 50(100 –50)** |
| --- | --- |
| 194 | **(5)^2^** |
| 195 | =**384** including 10% of non-response sample 384 plus 10%=38+384=422 |
| 196 |  |

197 This study employed a multistage sampling technique for the selection of the study units as
198 summarized in Figure 3. The sampling stages are zones, regions, councils, and public primary
199 health facilities. The first stage was a random selection of one region from each of the nine
200 Zones of the country. In the second stage, in each selected region, councils were clustered into
201 rural and urban, and then one rural and one urban council will be selected from each region
202 followed by a random selection of the health facilities, see Figure 3. The technique is convenient
203 for studying large and diverse populations (5). The sampling stages were zones, regions,
204 councils, and public primary health facilities.
205 ……………………………………………………..
206 Insert figure 3
207 ……………………………………………………

208

## 209 Variables and their measurements.

### 210 Dependent variable

211 The dependent variable of this study i.e. the use of health research evidence was obtained from a
212 set of four (4) questions that were measured. The questions were a mixture of binary and
213 multiple-choice questions. Those questions were divided into four areas. The mean score was
214 calculated from those sets of questions; the score had a value of 0 and 1. Those respondents who
215 scored 1 were regarded to have used the health research evidence, while those who scored 0 were
216 regarded to have not used the health research evidence. For multiple choice questions, the
217 respondents were provided with a set of predefined options, allowing them to select the one that
218 best represents their response, which will capture varying degrees or categories on the use of
219 health research evidence.

### 220 Independent Variables

221 The independent variables in this study encompass numerous factors including demographic
222 information such as sex, age, and education level; professional attributes such as position within
223 the health facility and years of schooling; and contextual elements including the type of health
224 facility, stakeholders group represented district, and region. The study aimed to gain insights into
225 the determinants that influence the use of health research evidence during health planning. The
226 determinants had a combination of three constructs which are, capability determinants,
227 opportunity determinants, and motivation determinants both derived from the COM-B Model.
228 **Capability determinants** were obtained from a set of twelve (12) Likert scale questions with a
229 scale of 1 to 5 (1 = strongly agree, 2 = somewhat agree, 3 = neither agree nor disagree, 4 =
230 somewhat disagree, 5 = strongly disagree) and one multiple choice question. The mean and
231 standard deviation of the responses to assess central tendency and variability were calculated.
232 For a multiple-choice question, the frequency distribution of each response category was
233 analyzed. Additionally, a chi-square test was calculated to determine if the distribution of
234 responses was significantly different from what would be expected by chance.
235 **Opportunity determinants,** were obtained from a set of seventeen (17) Likert scale questions
236 with a scale of 1 to 5 (1 = strongly agree, 2 = somewhat agree, 3 = neither agree nor disagree, 4 =
237 somewhat disagree, 5 = strongly disagree). We assigned numerical values (1 to 5) to these
238 responses, where lower values indicate stronger agreement. Next, the mean or median was
239 calculated of these numerical values to quantify the overall tendency. The multiple regression
240 analysis was used to assess the relationship between the independent variable and dependent
241 variables.
242 **Motivation determinants** were obtained from a set of eight (8) Likert scale questions with a
243 scale of 1 to 5 (1 = strongly agree, 2 = somewhat agree, 3 = neither agree nor disagree, 4 =
244 somewhat disagree, 5 = strongly disagree). We assigned numerical values (1 to 5) to these
245 responses, where lower values indicate stronger agreement. Next, the mean or median was
246 calculated of these numerical values to quantify the overall tendency. The multiple regression
247 analysis was used to assess the relationship between the independent variable and dependent
248 variables.

## 249 Data collection procedures and tools

250 Quantitative data was collected face to face person by using the Swahili version of the survey
251 after translation of the English version of the survey and document review checklist (see
252 supplemental file) to planning team members at national, regional, council, and health facility
253 levels. The questions from the tool were adopted and modified from previous studies (18,19).
254 The survey collected information on the use of health research evidence and its determinants
255 from the sampled participants using Open Data Kit software (ODK).
256 The document review checklist was used to guide the document review to see whether the
257 available plans have any evidence of being prepared using health research evidence. The
258 checklist contains a list of questions that will help the review of the plans made at the facility,
259 council, regional, and national levels. The checklist is used to collect information on the
260 available health plans if they are prepared by using health research evidence. Data was collected
261 by a research assistant, who was trained for three days in data collection methods, tools, and
262 ethics. They were selected according to their experience in the health system. Pilot testing of the
263 tools was conducted at Dodoma RHMT and Chamwino District Council at Chamwino CHMT,
264 Chamwino Council Hospital, Chamwino Health Centre, and Buigiri Dispensary to enhance the
265 validity, reliability, and effectiveness of the tool in capturing the intended information.

## 266 Data analysis

267 Responses were reported on all survey items in all response categories and summarized using
268 frequencies and percentages for categorical variables and means and standard deviation for non-
269 categorical variables were computed. Given that the outcome variable had two categories
270 (0=Negative, 1=Positive), A binary logistic regression model was used to assess factors
271 associated with the perception of implementers on the prime vendor system. The model results
272 are regression parameter estimates and odds ratios (OR). The data analysis was conducted using
273 STATA version 18, and the significance of all statistical tests was established at a 5%
274 significance level.

275

## 276 Data validity and reliability

277 To ensure validity and reliability, the validity of quantitative data collection tools was reviewed
278 by independent subject matter experts from the University of Dodoma (UDOM). The reliability
279 assessment of the questionnaire was done using the internal consistency test, with the alpha
280 reliability coefficient being the statistic. (20,21). The range of the alpha coefficient, also known
281 as Cronbach's alpha was 0.71 acceptable to this study. Moreover, exploratory factor analysis to
282 establish the construct validity of the questionnaire and pilot study conducted at Chamwino DC
283 before field data collection to ensure clarity of the data collection tool.

284

## 285 Ethics and Dissemination

286 The study was granted ethical approval by the University of Dodoma Ethical Committee with
287 **Ref. No. MA.84/261/102/’A’/64/91.** Permission to conduct the study and consent to participate
288 in the study was sought from relevant authorities and participants, respectively. Participants
289 received information about the purpose of the study and data protection. The findings of the
290 study were disseminated to relevant stakeholders through collaborative communication with the
291 Ministry of Health, RHMT/CHMT, Tanzania Health Summit as well as publications to target
292 researchers, practitioners, implementers, and policymakers.

293

# 294 Results

295 The finding from this study is presented in three parts using tables, percentages, and charts, Part
296 one shows the respondents' demographic characteristics and stakeholder group. Part two shows
297 the proportion of the respondents who reported using Health research evidence in health
298 planning and, part three shows the association between health research use in planning and the
299 COM-B constructs (capability, Motivation, and opportunity).

## 300 Demographic characteristics

301 Table 1 describes the respondents’ characteristics and the stakeholder group they represent.
302 There was an almost equal number of female 50.2 % and male 49.8% respondents with a mean
303 age of 39.51 ±7.86years old. Most of them (42.7%) had undergraduate education followed by a
304 Diploma (36.3%) and less than one-third (14.0%) had a master’s degree and above. Few (7.1%)
305 respondents ‘had certificate-level education. Professional background results revealed that the
306 majority 107 (25.4%) of the respondents were medical doctors followed by nurses 99 (23.5%).
307 Physiotherapists and panning offices were very few each accounting for 0. 24% of the
308 respondents.

309

310	Table 3: Demographic characteristics of respondents

| **Variable** | **Frequency** | **Percent** | **Mean ±SD** |
| --- | --- | --- | --- |
| **Age** |  |  | 39.51 ±7.86 |
| <25 | 3 | 0.7 |  |
| 26-35 | 159 | 37.7 |  |
| 36-45 | 156 | 37.0 |  |
| 46-55 | 93 | 22.4 |  |
| >46 | 11 | 2.6 |  |
| **Region** |  |  |  |
| Mbeya | 48 | 11.4 |  |
| Dodoma | 50 | 11.9 |  |
| Iringa | 41 | 9.7 |  |
| Mtwara | 49 | 11.6 |  |
| Mwanza | 50 | 11.85 |  |
| Kigoma | 51 | 12.1 |  |
| Kilimanjaro | 45 | 10.7 |  |
| Morogoro | 48 | 11.4 |  |
| Dar es salaam | 40 | 9.5 |  |
| **Type of health facility** |  |  |  |
| Dispensary | 37 | 8.8 |  |
| Health center | 82 | 19.4 |  |
| Council Hospital | 97 | 23.0 |  |
| Regional referral hospital | 81 | 19.2 |  |
| RHMT | 87 | 20.6 |  |
| CHMT | 38 | 9.0 |  |
| Gender |  |  |  |
| Male | 210 | 49.76 |  |
| Female | 212 | 50.24 |  |
| **Professional background** |  |  |  |
| Doctor | 107 | 25.36 |  |

| Nurse | 99 | 23.46 |  |
| --- | --- | --- | --- |
| Laboratory scientist | 36 | 8.53 |  |
| Pharmacist | 28 | 6.64 |  |
| Radiographer | 7 | 1.66 |  |
| Environmental Health officer | 13 | 3.08 |  |
| Nutrition officer | 13 | 3.08 |  |
| Social Welfare Officer | 20 | 4.74 |  |
| Physiotherapist | 1 | 0.24 |  |
| Biomedical engineer | 3 | 0.71 |  |
| Planning officer | 1 | 0.24 |  |
| Health Secretary | 38 | 9.00 |  |
| Other | 56 | 13.3 |  |
| **Stakeholders' group** |  |  |  |
| RHMT | 49 | 11.6 |  |
| HMT | 280 | 66.4 |  |
| CHMT | 90 | 21.3 |  |
| FBO | 1 | 0.2 |  |
| HGFC | 2 | 0.5 |  |
| **Highest level of education** |  |  |  |
| Certificate | 30 | 7.1 |  |
| Diploma Certificate | 153 | 36.3 |  |
| Undergraduate degree | 180 | 42.7 |  |
| Master’s Degree | 56 | 13.3 |  |
| PhD | 3 | 0.7 |  |
| **The position holds currently in health planning.** |  |  |  |
| Chairperson | 30 | 7.1 |  |
| Secretary | 45 | 10.7 |  |
| Technical Advisor | 11 | 2.6 |  |
| Member | 335 | 79.4 |  |
| Other | 1 | 0.2 |  |
| **Years participate** |  |  | 5.08 ±3.96 |
| <5 | 283 | 67.1 |  |
| 6-10 | 103 | 24.4 |  |
| >10 | 36 | 8.5 |  |

311

## 312 Proportion of the use of health research evidence

313 **Table 4** provides data on the use of health research evidence in a particular context. It shows that
314 the majority of participants (96.7%) have used routine data, with 49.5% reporting a high extent
315 of use. Additionally, evidence from routine data was the most commonly used type of evidence
316 during health planning, with 98.8% of participants reporting its use. Most participants (66.2%)
317 have used health research evidence as shown in Figure 4, and a significant majority (98.8%)
318 have used health planning guidelines. Moreover, the majority of participants (98.8%) also
319 indicated the importance of the use of health research evidence, with 82.6% considering it very
320 important. These findings suggest a strong reliance on routine data and a high recognition of the
321 importance of health research evidence in the context studied.
322 Table 4: The proportion of the use of health research evidence during health planning

| Variable | Frequency | Percent |
| --- | --- | --- |
| **Ever used routine data** |  |  |
| No | 14 | 3.3 |
| Yes | 408 | 96.7 |
| **Extent of use of routine data** |  |  |
| Low | 41 | 10.1 |
| Medium | 165 | 40.4 |
| High | 202 | 49.5 |
| **Type of evidence used during health planning (Multiple response)** |  |  |
| Evidence from routine data | 403 | 98.8 |
| Policy documents | 280 | 68.6 |
| Research publications (general) | 133 | 32.6 |
| Systematic reviews/ meta-analysis | 41 | 10.1 |
| Randomized Control trials | 17 | 4.2 |
| Experimental studies | 30 | 7.4 |
| Non-experimental studies | 20 | 4.9 |
| Expert opinions | 271 | 66.4 |
| Policy beliefs | 162 | 39.7 |
| Other | 17 | 4.2 |
| **Use of health research evidence** |  |  |
| No | 138 | 33.8 |
| Yes | 270 | 66.2 |
| **Use of the Health planning guidelines** |  |  |
| No | 5 | 1.2 |
| Yes | 403 | 98.8 |
| **Use of the Ruling party manifesto** |  |  |
| No | 44 | 10.8 |
| Yes | 364 | 89.2 |
| **Use of the Policy documents** |  |  |
| No | 137 | 33.6 |
| Yes | 271 | 66.4 |
| **Importance of the use of health research evidence** |  |  |
| No | 5 | 1.2 |
| Yes | 403 | 98.8 |
| **Level of importance of the use of health research evidence** |  |  |
| Very unimportant | 5 | 1.2 |
| Unimportant | 2 | 0.5 |

| Neutral | 14 | 3.4 |
| --- | --- | --- |
| Important | 50 | 12.3 |
| Very important | 337 | 82.6 |

323

324

325

326 **………………………………………………………………………………**

### 327 Insert figure 4

328 **………………………………………………………………………………..**

329

330 **Figure 4**: Proportion of the use of health research evidence.

## 331 The factors associated with the use of health research evidence during health

332 **planning.**
333 The table presents the results of a binary logistic regression analysis examining factors
334 associated with the use of research evidence during health planning. The analysis includes
335 variables such as age, region, type of health facility, gender, professional background,
336 stakeholder group, highest level of education, position held in health planning, and years of
337 participation. The adjusted odds ratios (OR), confidence intervals (CI), and p-values are provided
338 to indicate the strength and statistical significance of associations.
339 *Significant Variables and Interpretation*
340 •	**Region**: The analysis compares various regions to the reference category, Kilimanjaro.
341 The odds ratios (OR) show how likely individuals from other regions are to use health
342 research evidence in health planning compared to those from Kilimanjaro. For instance, if
343 a region has an OR greater than 1, individuals from that region are more likely to use
344 research evidence than those from Kilimanjaro. If the OR is less than 1, they are less
345 likely. The p-values indicate whether these differences are statistically significant.
346 •	**Level of Education**: Compared to individuals with a diploma (reference category), those
347 with higher education levels were more likely to use research evidence:

348	⮚ Undergraduate degree (OR = 2.396, CI: [1.094, 5.249], p-value = 0.0290):

349	Those with an undergraduate degree are over twice as likely to use

350	research evidence compared to diploma holders.

351	⮚ Master's degree (OR = 2.278, CI: [0.921, 5.631], p-value = 0.0747):

352 Similar to undergraduates, master's degree holders are also more likely to
353 use research evidence in health planning although were not significant.
354 These OR values reflect the increased likelihood of individuals with higher education
355 levels using research evidence compared to the reference group, with p-values indicating
356 the strength of this relationship.

### 357 • Years of Participation:

358	⮚ 6–10 years (OR = 1.092, CI: [0.677,1.761], p-value = 0.7184): Individuals

359	with 6–10 years of participation in health planning show a higher

360	likelihood of using research evidence compared to those with fewer years,

361	though this was not statistically significant.

362	⮚ More than 10 years (OR = 1.399, CI: [0.648,3.019], p-value = 0.3924):

363 Those with over 10 years of participation have a 40% higher likelihood of
364 using research evidence compared to those with fewer years of experience,
365 and this difference was also not statistically significant.
366 The adjusted odds ratios reflect the likelihood of each category of the variable influencing the
367 use of research evidence, controlling for other variables in the model. Significance is determined
368 by both the size of the OR and the p-value, with confidence intervals providing a range within
369 which the true effect likely falls.
370 Table 5: Binary logistic regression for factors associated with the use of research evidence during
371 health planning.

372

| Variable | Not use | Use | Unadjusted logistic analysis | | Adjusted logistic analysis | |
| --- | --- | --- | --- | --- | --- | --- |
|  | N (%) | N (%) | OR [95%CI] | p-  value | OR [95%CI] | p-value |
| **Age** |  |  |  |  |  |  |
| <35 | 52(32.1) | 110(67.9) | ref |  |  |  |
| 36-45 | 56(35.9) | 100(64.1) | 0.844[0.530,1.343] | 0.4747 |  |  |
| >45 | 35(33.7) | 69(66.4) | 0.932[0.552,1.573] | 0.7919 |  |  |
| **Region** |  |  |  |  |  |  |
| Kilimanjaro | 26(57.8) | 19(42.2) | ref |  | ref |  |
| Mbeya | 17(35.4) | 31(64.6) | 2.495[1.081,5.760] | 0.0322 | 2.604[1.074,6.315] | 0.0342 |
| Dodoma | 17(34.0) | 33(66.0) | 2.656[1.156,6.104] | 0.0214 | 2.514[1.053,6.001] | 0.0378 |
| Iringa | 10(24.4) | 31(75.6) | 4.242[1.680,10.71] | 0.0022 | 4.707[1.772,12.499] | 0.0019 |
| Mtwara | 21(42.9) | 28(57.1) | 1.825[0.805,4.137] | 0.1499 | 1.801[0.727,4.466] | 0.2039 |
| Mwanza | 12(24.0) | 38(76.0) | 4.333[1.801,10.43] | 0.0011 | 4.794[1.893,12.140] | 0.0009 |
| Kigoma | 10(19.6) | 41(80.4) | 5.611[2.259,13.94] | 0.0002 | 5.922[2.224,15.769] | 0.0004 |
| Morogoro | 17(35.4) | 31(64.6) | 2.495[1.081,5.760] | 0.0322 | 2.180[0.901,5.274] | 0.0839 |
| Dar es salaam | 13(32.5) | 27(67.5) | 2.842[1.170,6.904] | 0.0211 | 3.229[1.211,8.611] | 0.0192 |
| **Type of health facility** |  |  |  |  |  |  |
| Dispensary | 17(46.0) | 20(54.1) | ref |  | ref |  |
| Health center | 25(30.5) | 57(69.5) | 1.938[0.871,4.311] | 0.1048 | 2.029[0.829,4.964] | 0.1211 |
| Council  Hospital | 45(46.4) | 52(53.6) | 0.982[0.459,2.100] | 0.9631 | 0.809[0.323,2.026] | 0.6511 |
| RRH | 19(23.5) | 62(76.5) | 2.774[1.214,6.335] | 0.0155 | 2.663[1.895,7.920] | 0.0383 |
| RHMT | 27(31.0) | 60(69.0) | 1.889[0.857,4.163] | 0.1147 | 0.350[0.031,3.902] | 0.3932 |
| CHMT | 10(26.3) | 28(73.7) | 2.380[0.903,6.273] | 0.0795 | 5.647[0.602,52.94] | 0.3167 |
| Gender |  |  |  |  |  |  |
| Male | 71(33.8) | 139(66.2) | 1.007[0.673,1.507] | 0.9736 |  |  |
| Female | 72(34.0) | 140(66.0) | ref |  |  |  |
| **Professional background** |  |  |  |  |  |  |
| Doctor | 37(34.6) | 70(65.4) | ref |  |  |  |
| Nurse | 35(35.4) | 64(64.7) | 0.967[0.545,1.714] | 0.9073 |  |  |
| Lab scientist  &Pharmacist | 25(35.2) | 46(64.8) | 0.973[0.518,1.825] | 0.9310 |  |  |
| Non-medical | 28(31.5) | 61(68.5) | 1.152[0.633,2.096] | 0.6444 |  |  |
| Other | 18(32.1) | 38(67.7) | 1.116[0.561,2.220] | 0.7548 |  |  |
| **Stakeholders' group** |  |  |  |  |  |  |
| RHMT | 13(26.5) | 36(73.5) | 1.609[0.816,3.171] | 0.1697 | 0.843[0.177,4.012] | 0.8297 |
| CHMT | 26(28.9) | 64(71.1) | 1.430[0.854,2.396] | 0.1741 | 5.647[0.602,52.93] | 0.1295 |
| HMT | 104(36.8) | 179(63.3) | ref |  | ref |  |
| **Highest level of education** |  |  |  |  |  |  |
| Certificate | 15(50.0) | 15(50.0) | ref |  | ref |  |
| Diploma | 57(37.3) | 96(62.8) | 1.684[0.767,3.700] | 0.1943 | 1.609[0.670,3.867] | 0.2874 |
| Undergraduate | 53(29.4) | 127(70.6) | 2.396[1.094,5.249] | 0.0290 | 1.824[0.710,4.685] | 0.2120 |

| Master’s  Degree | 18(30.5) | 41(69.5) | 2.278[0.921,5.631] | 0.0747 | 2.082[0.684,6.337] | 0.1968 |
| --- | --- | --- | --- | --- | --- | --- |
| **Position holds currently in health planning** |  |  |  |  |  |  |
| Secretary | 19(42.2) | 26(57.8) | ref |  | ref |  |
| Chairperson | 8(26.7) | 22(73.3) | 2.009[0.738,5.475] | 0.1724 | 1.933[0.647,5.780] | 0.2381 |
| Technical | 4(36.4) | 7(63.6) | 1.279[0.327,5.000] | 0.7236 | 0.789[0.173,3.599] | 0.7600 |
| Member | 112(33.3) | 224(66.7) | 1.462[0.776,2.754] | 0.2404 | 1.328[0.651,2.708] | 0.4354 |
| **Years participate** |  |  |  |  |  |  |
| <5 | 99(35.0) | 184(65.0) | ref |  |  |  |
| 6-10 | 34(33.0) | 69(67.0) | 1.092[0.677,1.761] | 0.7184 |  |  |
| >10 | 10(27.8) | 26(72.2) | 1.399[0.648,3.019] | 0.3924 |  |  |

373

374

### 375 Capability: The knowledge and skills of health research evidence use

376 Table 6 presents the findings from a survey on the capacity to use health research evidence
377 (knowledge and skills). Each row in the table represents a different variable related to the use of
378 health research evidence, and the columns display the responses categorized into five levels of
379 importance: Very Unimportant (VUIM), Unimportant (UIM), Neutral, Important (IM), and Very
380 Important (VIM).
381 From the data, it's clear that the majority of respondents perceive the use of health research
382 evidence as very important or important across the various variables. In the variable "Gives the
383 latest information," the majority of respondents rated it as Very Important (65.7%) or Important
384 (24.5%). Similarly, in the variable "Help the policymakers understand a specific problem," a
385 substantial proportion of respondents indicated that it is Very Important (66.7%) or Important
386 (24.3%). Overall, the data suggests that there is a strong recognition of the significance of health
387 research evidence in various aspects of policymaking and intervention implementation.

388

389	Table 6: Knowledge and skills of health research evidence use

| Variable | VUIM n  (%) | UIM n  (%) | Neutral n  (%) | IM n (%) | VIM n (%) |
| --- | --- | --- | --- | --- | --- |
| Gives the latest information | 5(1.2) | 3(1.2) | 32(7.8) | 100(24.51) | 268(65.7) |
| Help the policymakers  understand a specific problem | 3(0.7) | 4(1.0) | 30(7.4) | 99(24.3) | 272(66.7) |
| Help the policymakers to | 3(0.7) | 5(1.2) | 24(5.9) | 113(27.7) | 263(64.5) |

| implement various health  interventions |  |  |  |  |  |
| --- | --- | --- | --- | --- | --- |
| Avoid repeating the failures of  others | 3(0.7) | 3(0.7) | 19(4.7) | 101(24.8) | 282(69.1) |
| Introduces health planners to  new ideas | 3(0.7) | 2(0.5) | 19(4.7) | 107(26.2) | 277(67.9) |

390

391 Table 7 provides information on the obstacles hindering the utilization of health research
392 evidence. The variables include Lack of necessary knowledge and training in research,
393 Inadequate human and non-human resources, Difficulty in accessing health research evidence,
394 Perceptions that health research is for academic purposes, Lack of involvement in research
395 activities, and Traditional ways of planning, as well as the minimal usage of health research
396 evidence among planning team members at the regional and council levels, and lack of
397 dissemination.
398 The data is presented in terms of the number and percentage of participants who strongly
399 disagree (SDSA), disagree (DSA), are neutral, agree (AG), and strongly agree (SAGR) with each
400 obstacle. This data provides insight into the specific challenges that impact the utilization of
401 health research evidence within the context of the study. The highest percentage is for
402 "Perceptions that health research is for academic purposes" at 22.6%, and the lowest percentage
403 is for "Lack of necessary knowledge and training in research" at 2.9%.

404

### 405 Table 7: Obstacles that hinder the utilization of health research evidence

| Variable | SDSA n (%) | DSA n  (%) | Neutral n  (%) | AG n (%) | SAGR n  (%) |
| --- | --- | --- | --- | --- | --- |
| Lack of necessary knowledge  and training in research | 12(2.94) | 18(4.4) | 7(1.7) | 67(16.4) | 304(74.5) |
| Inadequate human and non-  human resources | 16(3.92) | 18(4.4) | 7(1.7) | 81(19.9) | 286(70.1) |
| Difficult in accessing health  research evidence | 36(8.82) | 41(10.1) | 24(5.9) | 110(27.0) | 197(48.3) |
| Perceptions that health research | 92(22.55) | 40(9.8) | 16(3.9) | 81(19.9) | 179(43.9) |

| is for academic purposes |  |  |  |  |  |
| --- | --- | --- | --- | --- | --- |
| Lack of involvement in research  activities | 15(3.68) | 25(6.1) | 11(2.7) | 93(22.8) | 264(64.7) |
| Traditional ways of planning, and Minimal usage of health research evidence among planning team members at the  regional and council levels | 36(8.82) | 21(5.2) | 13(3.2) | 92(22.6) | 246(60.3) |
| lack of dissemination | 11(2.70) | 15(3.7) | 17(4.2) | 105(25.7) | 260(63.7) |

406

407

## 408 Opportunities for the use of health research evidence

409 Table 8 indicates the opportunities for the use of health research evidence, helping stakeholders
410 to identify areas for improvement and investment to optimize the use of health research
411 evidence. The table presents the opportunities for utilizing health research evidence, categorized
412 into physical and social opportunities.
413 For physical opportunities, the table highlights the availability of resources such as research
414 coordinators, equipment, internet access, a planned budget for research, and the presence of
415 symposiums for research discussions. The percentages indicate the distribution of responses,
416 ranging from strongly disagree to strongly agree, reflecting the varying levels of agreement
417 among the respondents regarding these physical opportunities. In the physical opportunities
418 category, the highest percentage is 76.3%, representing the availability of the internet, while the
419 lowest percentage is 0.7%, indicating the availability of Internet.
420 In the social opportunities section, the table emphasizes the presence of disparities among
421 different populations, the involvement of community members in health planning teams, and the
422 enhancement of health literacy in society. The percentages provide an insight into the attitudes
423 and perceptions of the respondents toward these social opportunities. In the social opportunities
424 category, the highest percentage is 55.9%, reflecting improved health literacy in society, and the
425 lowest percentage is 2.4%, which represents the presence of disparities among different
426 populations allowing for more equitable representation.
427 Table 8: Opportunities for the use of health research evidence

| Variable | SDSA n  (%) | DSA n  (%) | Neutral n  (%) | AG n (%) | SAGR n  (%) |
| --- | --- | --- | --- | --- | --- |
| **Physical opportunities** |  |  |  |  |  |
| Enabling the utilization of information  generated from the healthcare system | 6(1.4) | 9(2.1) | 18(4.3) | 130(30.8) | 259(61.4) |
| Availability of research coordinators | 9(2.1) | 18(4.3) | 22(5.2) | 93(22.0) | 280(66.4) |
| Availability of equipment | 4(1.0) | 16(3.8) | 21(5.0) | 88(20.9) | 293(69.4) |
| Availability of internet | 3(0.7) | 9(2.1) | 19(4.5) | 69(16.4) | 322(76.3) |
| Availability of planned budget for  research | 11(2.6) | 17(4.0) | 27(6.4) | 64(15.2) | 303(71.8) |
| Presence of symposiums for research  discussions | 9(2.1) | 17(4.0) | 23(5.5) | 106(25.1) | 267(63.3) |
| **Social opportunities** |  |  |  |  |  |
| The presence of disparities among different populations allows for more  equitable | 10(2.4) | 24(5.7) | 38(9.0) | 147(34.8) | 203(48.1) |
| The presence of community members  in the health planning teams | 11(2.6) | 28(6.6) | 55(13.0) | 119(28.2) | 209(49.5) |
| Improved health literacy in the society, empowering individuals to  make informed | 4(1.0) | 19(4.5) | 18(4.3) | 145(34.4) | 236(55.9) |

428

## 429 Motivations for the use of health research evidence

430 Table 9, based on the data presented, we can observe that the automatic motivations for the use
431 of health research evidence are primarily centered around the availability of transparency and
432 accountability mechanisms 66.4% strongly agreeing, and 23.2% agreeing with the statement
433 availability of job training 73.2% strongly agreeing and 21.3% agreeing respectively. Provision
434 of incentives to health planners, with 72.4% strongly agreeing and 15.4% agreeing respectively.
435 In contrast, reflective motivations such as continuous quality improvement in the healthcare
436 system and the presence of active stakeholder engagement in health planning also garnered
437 significant support, with 63.51% and 59.48% strongly agreeing or agreeing, respectively. This
438 indicates a strong emphasis on the proactive involvement of various stakeholders and the
439 continuous enhancement of the healthcare system based on research evidence.

440

441	Table 9: Motivations of the use of health research evidence

| Variable | SDSA n (%) | DSA n (%) | Neutral n (%) | AG n (%) | SAGR n (%) |
| --- | --- | --- | --- | --- | --- |
| **Automatic motivations** |  |  |  |  |  |
| Provision of incentives to health  planners | 19(4.5) | 21(5.0) | 13(3.1) | 65(15.4) | 304(72.0) |
| Availability of job training | 3(0.7) | 4(1.0) | 16(3.8) | 90(21.3) | 309(73.2) |
| Availability of Short-term courses | 5(1.2) | 13(3.1) | 21(5.0) | 75(17.8) | 308(73.0) |
| Availability of Long-term course | 18(4.3) | 24(5.7) | 24(5.7) | 105(24.9) | 251(59.5) |
| **Reflective motivations** |  |  |  |  |  |
| Presence of interaction between policymakers, implementers,  researchers, and academicians | 3(0.7) | 17(4.0) | 21(5.0) | 111(26.3) | 270(64.0) |
| Presence of active stakeholder  engagement in health planning | 6(1.4) | 16(3.8) | 33(7.8) | 116(27.5) | 251(59.5) |
| Continuous quality improvement in  the healthcare system | 1(0.2) | 10(2.4) | 12(2.8) | 131(31.0) | 268(63.5) |
| Availability of transparency and  accountability mechanisms | 5(1.2) | 12(2.8) | 27(6.4) | 98(23.2) | 280(66.4) |

442

## 443 Findings from document review checklist

444 The analyzed data from Table 10 indicates that the majority of planning teams incorporate
445 research evidence into their health plans. A substantial 97.8% of respondents confirmed having a
446 current fiscal year health plan, and 98.9% reported having a planning guideline. Most plans
447 include key health frameworks and guides, with 70.8% having the CCHP, 83.2% using the HF
448 Planning Guide, and 89.9% incorporating situational analysis. Scientific engagement is
449 evidenced by 75.3% of teams receiving invitation letters for conferences. However, challenges
450 remain, as only 22.5% have a budget for research, and only 23.6% allocate budget specifically
451 for research within the health plan. Additionally, just 28.1% include a research coordinator in the
452 planning team, and 43.8% of the plans are well-cited. This highlights a strong foundation in
453 research usage but also gaps in financial and personnel support for research-driven planning.
454 Table 10: looking for evidence that planning teams use health research evidence in their plans

| Variable | No | Yes | Not applicable |
| --- | --- | --- | --- |
|  | n (%) | n (%) | n (%) |
| Do you have a current fiscal year health plan? | 1(1.1) | 87(97.8) | 1(1.1) |
| Do you have a planning guideline? | 1(1.12) | 88(98.9) | 0(0.0) |
| Presence of CCHP | 12(13.5) | 63(70.8) | 14(15.7) |
| HF Planning Guide | 13(14.6) | 74(83.2) | 2(2.3) |
| RHMT guide | 26(29.2) | 20(22.5) | 43(48.3) |
| CHOP | 30(33.7) | 24(27.0) | 35(39.3) |
| Presence of situational analysis in the plan | 8(9.0) | 80(89.9) | 1(1.1) |
| Presence of invitation letters to attend various  scientific conferences | 22(24.7) | 67(75.3) | 0(0.0) |
| Are the well-cited plans | 50(56.2) | 39(43.8) | 0(0.0) |
| Is there a budget for research available | 69(77.5) | 20(22.5) | 0(0.0) |
| Is there a research-allocated budget in their  health plan? | 68(76.4) | 21(23.6) | 0(0.0) |
| Is the research coordinator among the planning  team members who prepared the plan | 64(71.9) | 25(28.1) | 0(0.0) |

455

456

# 457 Discussion

458 This study represents the first comprehensive analysis of the use of health research evidence in
459 Tanzania. It aimed to analyze the determinants of the use of health research evidence by deep-
460 diving into the three determinants influencing behavior i.e. Capability, Opportunity, and
461 Motivation. The findings from this study indicated that all three determinants significantly
462 impact the ability of health planners to use health research evidence during health planning.
463 However, we found that the use of research evidence is associated with having a degree, more
464 than 10 years of involvement in health planning activities, and prior knowledge in research. We
465 found several facilitator determinants such as organizational support infrastructures like, the
466 availability of computers and the internet, the availability of research experts and research
467 coordinators, availability of academic institutions that give a chance for dissemination of
468 research. On the other hand, barrier determinants included lack of human resources, lack of
469 funding for research activities, lack of necessary knowledge and training, perceptions that
470 research is for academic purposes, and lack of dissemination.

## 471 Proportion of the use of health research evidence

472 The findings of this study on the proportion of the use of health research evidence demonstrate a
473 high level of reliance on health research evidence, particularly routine data, during health
474 planning. The majority of participants (96.7%) reported using routine data, with nearly half
475 (49.5%) using it extensively, which is consistent with findings from similar studies. For instance,
476 a study by Lavis et al (22) found that routine data and health statistics are frequently utilized in
477 policy-making and health-planning processes, underscoring the central role of such data in
478 evidence-based decision-making. Moreover, the widespread use of health planning guidelines
479 (98.8%) and the high proportion of participants (98.8%) recognizing the importance of health
480 research evidence align with findings from studies on the use of research evidence in health
481 decision-making process which emphasize the role of guidelines and evidence in shaping health
482 policies (23,24). The high rate of health research evidence use (66.2%) in this study also mirrors
483 the findings which reported similar levels of research evidence use in healthcare settings(25).
484 These results highlight the growing recognition and integration of research evidence in health
485 planning, reflecting broader trends in evidence-informed policymaking across health systems in
486 Tanzania and globally.

## 487 Capability: The knowledge and skills of health research evidence use

488 Explicitly, capability determinants such as knowledge and skills were found to be critical, with
489 health planning team members who had higher levels of education (Undergraduate, a master
490 level, and above) and access to training more likely to use health research evidence to almost
491 70%, with the capability constraints faces more those with certificate and diploma level, lack of
492 necessary knowledge and training in research accounts up to 70%, being the most barrier to
493 utilization of health research evidence, inadequate human and non-human resources, difficult in
494 accessing research evidence, perception that research is for academic purposes and lack of
495 dissemination. These findings are consistent with systematic reviews conducted in other low-
496 and middle-income countries, where similar capability constraints such as poor access to good
497 quality relevant research, and lack of timely research output have been identified as major
498 barriers to evidence-based health planning (26,27).

499

## 500 Opportunities for the use of health research evidence

501 In comparing our results with existing literature on Opportunity determinants (both physical and
502 social), it is evident that the availability of resources and institutional support plays a crucial role
503 in enabling the use of research evidence. Our study revealed that health planning teams with
504 better access to physical opportunities such as health information system, availability of research
505 coordinators, equipment, internet, research budgets, presence of symposiums for research
506 discussion, the existence of academic institutions such as universities, and social opportunities
507 like the presence of community members representatives within the health planning teams and
508 improved health literacy in the community were more inclined to utilize research findings in
509 their work. This aligns with findings from two studies conducted in LMICs (28,29), which
510 highlighted the importance of institutional support in facilitating the use of research evidence in
511 health policy. However, our study also noted a distinct challenge in the Tanzanian context with
512 limited access to relevant health research evidence due to infrastructural constraints, a barrier
513 less emphasized in studies from more resource-rich settings (30,31).

## 514 Motivations for the use of health research evidence

515 Particularly the automatic motivations to utilize health research evidence were also found to be
516 significant, especially on the provision of incentives to health planners to greater than 72%.
517 Other motivations include job training, and short and long-term courses that influenced the
518 motivations to use the health research evidence. The automatic motivation factors included the
519 presence of interaction between policymakers, implementers, researchers, and academicians, and
520 continuous quality improvement agenda in the health sector. Health planners who perceived the
521 use of research evidence as beneficial and aligned with their professional goals were more likely
522 to engage with it actively. This is in line with the COM-B model, which underscores the role of
523 motivation in behavior change (10). Our findings add to the body of literature by highlighting the
524 specific motivational barriers in Tanzania, such as a lack of a clear roadmap or framework for
525 the utilization of health research evidence, which can reduce the enthusiasm for utilizing research
526 in health planning (32)(33).

## 527 Document review checklist

528 The findings from the documents review checklist revealed a high level of integration of research
529 evidence in health planning aligning with similar studies that underscore the importance of
530 research-informed frameworks in public health planning (34)(35). The near-universal adoption
531 of fiscal-year health plans (97.8%) and planning guidelines (98.9%) is consistent with global
532 trends in evidence-based health planning, which emphasizes standardized planning processes
533 (36). The presence of documents like CCHP and HF Planning guide mirrors findings from an
534 urban strategic planning using blue-green infrastructure (37), who highlighted their role in
535 improving health outcomes, the limited financial resources dedicated to health research in this
536 study 22.5% suggests a common barrier. Similarly, the low inclusion of research coordinators
537 (28.1%) echoes observations by a study designed for research approach during learning (38) on
538 the limited interdisciplinary engagement in health planning. These gaps in budget allocation and
539 personnel highlight persistent structural challenges to fully operationalizing research evidence-
540 backed plans, despite a broad commitment to evidence-based frameworks.
541 The study provided valuable insights into the factors influencing the use of health research
542 evidence during health planning in Tanzania. The results from the binary logistic regression
543 analysis highlight significant associations between region, level of education, and years of
544 participation in health planning. Notably, region was a significant predictor, with participants
545 from regions other than Kilimanjaro demonstrating varying likelihoods of using research
546 evidence, as indicated by the odds ratios. Educational attainment also emerged as a key
547 determinant; individuals with undergraduate degrees were over twice as likely to use health
548 research evidence compared to those with a diploma, with statistically significant results (p-value

549	= 0.029). However, while master's degree holders showed a similar trend, the relationship was

550 not statistically significant (p-value = 0.0747). The analysis of years of participation revealed
551 that those with more than 10 years of experience were 40% more likely to use health research
552 evidence, though this association was not statistically significant. These findings align with other
553 studies that emphasize the importance of education and experience in evidence-based health
554 planning (24,26). The adjusted odds ratios and confidence intervals provide a nuanced
555 understanding of the strength and variability of these associations, contributing to a growing
556 body of literature on the determinants of research utilization in public health policy and planning.
557 The implications of these findings are essential for improving health planning in Tanzania. To
558 enhance the use of health research evidence, interventions must address all three determinants:
559 enhancing the capability of health planners through capacity building, improving opportunities
560 by ensuring better access to human and non-human resources and organizational support, and
561 fostering motivation by recognizing and rewarding health planners that use evidence-based
562 decision-making. These strategies align with recommendations from similar studies in other
563 LMICs, suggesting that a multifaceted approach including the development of middle range
564 theory (MRT) outlining the main facilitators for utilization of evidence for malaria treatment
565 policy in Uganda and the importance of measuring total brain activity in neuroimaging are
566 essential for promoting the use of research in health planning (39)(40).

# 567 Conclusion

568 This study provides a comprehensive assessment of the determinants influencing the use of
569 health research evidence in Tanzania, focusing on capability, opportunity, and motivation. The
570 findings demonstrate that all three determinants significantly impact the behavior of health
571 planners, highlighting those factors such as higher education levels, years of experience in health
572 planning, and prior research knowledge contribute to greater use of health research evidence.
573 Organizational infrastructure and institutional support further facilitate this process, while
574 barriers include insufficient human resources, lack of funding, inadequate training, and
575 perceptions that research is solely for academic purposes.
576 The study also reveals a high reliance on routine data and health planning guidelines, indicating
577 the growing recognition of research evidence in health decision-making. However, addressing
578 the barriers to research evidence use will require targeted interventions that strengthen the
579 capability of health planners, improve access to resources and institutional support, and enhance
580 motivation through incentives and professional development.
581 Future research should explore the long-term impact of these interventions, as well as contextual
582 factors like political will and leadership, to fully understand how these determinants collectively
583 shape health planning behavior. A multifaceted approach i.e. a generic framework for
584 strengthening Knowledge Management in Tanzania (KMT) that will have a triad pathway:
585 Capacity building on healthcare workers and researchers, infrastructure development, and
586 funding that will improve access to health research database and resources within the healthcare
587 system, and organizational support through the establishment of guidelines for the use of health
588 research evidence in the decision-making process. This is essential for promoting the use of
589 health research evidence, ultimately improving health outcomes in Tanzania and other low- and
590 middle-income countries with similar challenges (41,42)

591

592	**Abbreviations**

CCHP	Comprehensive Council Health Planning

CHOP	Comprehensive Hospital Operational Plan

LGAs	Local Government Authorities

LMICs	Lower-and Middle-Income Countries

MoH	Ministry of Health

PO-RALG	President’s Office-Regional Administration and

Local Government

RHMT	Regional Health Management Team

SDGs	Sustainable Development Goals

UHC	Universal Health Coverage

WHO	World Health Organization

593

## 594 Acknowledgments

595 We would like to acknowledge the contribution of the University of Dodoma faculty members
596 from the School of Nursing and Public Health for their input in the process of development of
597 this manuscript. We would like to express our sincere thanks to the study facilities, the
598 RHMTs, and the CHMTs for their support. We are grateful to the research assistants (Edmund
599 Bunyaga, Theresia Ngungulu, Jestina Nyondo, Devina Mafuta Micky Masanyaji, and Ally
600 Kinyaga) for their assistance during data collection.

## 601 Author Contributions

602 PK; Conceptualization, design of the work, data collection and analysis, preparing the
603 manuscript draft, developing the methodology, results, discussion, and conclusion. RM,
604 Conceptualization, critically reviewing the methodology and critically reviewing the manuscript,
605 AK, Conceptualization, critically reviewing the methodology. All authors revised the work and
606 approved the paper to be submitted for publication.

# 607 REFERENCES

608 1.	Khalid FJI. The Impact of Poor Planning and Management on the Duration of Construction
609 Projects. Multi-Knowl Electron Compr J Educ Sci Publ [Internet]. 2019;(2):1–20. Available
610 from: [www.mecsj.com](http://www.mecsj.com/)
611 2.	Onwujekwe O, Etiaba E, Mbachu C, Arize I, Nwankwor C, Ezenwaka U, et al. Does
612 improving the skills of researchers and decision-makers in health policy and systems
613 research lead to enhanced evidence-based decision making in Nigeria?—A short term
614 evaluation. Oelke N, editor. PLOS ONE [Internet]. 2020 Sep 3 [cited 2024 Feb
615 16];15(9):e0238365. Available from: https://dx.plos.org/10.1371/journal.pone.0238365
616 3.	Henriksson DK, Peterson SS, Waiswa P, Fredriksson M. Decision-making in district health
617 planning in Uganda: Does use of district-specific evidence matter? Health Res Policy Syst.
618 2019 Jun 6;17(1).
619 4.	Kagoma P, Mongi R, Kapologwe NA, Kengia J, Kalolo A. Health research evidence: its
620 current usage in health planning, determinants and readiness to use knowledge translation
621 tools among health planning teams in Tanzania—an exploratory mixed-methods study
622 protocol. BMJ Open [Internet]. 2024 Jun [cited 2024 Jun 27];14(6):e081517. Available from:
623 https://bmjopen.bmj.com/lookup/doi/10.1136/bmjopen-2023-081517
624 5.	Kengia JT, Kalolo A, Barash D, Chwa C, Hayirli TC, Kapologwe NA, et al. Research
625 capacity, motivators and barriers to conducting research among healthcare providers in
626 Tanzania’s public health system: a mixed methods study. Hum Resour Health [Internet].
627 2023 Sep 5 [cited 2024 Feb 9];21(1):73. Available from: https://human-resources-
628 health.biomedcentral.com/articles/10.1186/s12960-023-00858-w
629 6.	Van De Goor I, Hämäläinen RM, Syed A, Juel Lau C, Sandu P, Spitters H, et al.
630 Determinants of evidence use in public health policy making: Results from a study across six
631 EU countries. Health Policy [Internet]. 2017 Mar [cited 2024 Feb 26];121(3):273–81.
632 Available from: https://linkinghub.elsevier.com/retrieve/pii/S0168851017300192
633 7.	Van Kammen J, Savigny DD, Sewankambo N. Using knowledge brokering to promote
634 evidence-based policy-making: the need for support structures. Bull World Health Organ.
635 2006;84(8).
636 8.	Guilbert JJ. The World Health Report 2006: Working together for health [1]. Educ Health

637	Change Learn Pract. 2006;19(3):385–7.

638 9.	Mboera LEG, Rumisha SF, Mbata D, Mremi IR, Lyimo EP, Joachim C. Data utilisation and
639 factors influencing the performance of the health management information system in
640 Tanzania. BMC Health Serv Res. 2021;21(1):4–11.
641 10. Michie S, van Stralen MM, West R. The behaviour change wheel: A new method for
642 characterising and designing behaviour change interventions. Implement Sci. 2011 Apr
643 23;6(1).
644 11. De Leo A, Bayes S, Bloxsome D, Butt J. Exploring the usability of the COM-B model and
645 Theoretical Domains Framework (TDF) to define the helpers of and hindrances to evidence-
646 based practice in midwifery. Implement Sci Commun [Internet]. 2021 Dec [cited 2024 Aug
647 12];2(1):7. Available from:
648 https://implementationsciencecomms.biomedcentral.com/articles/10.1186/s43058-020-
649 00100-x
650 12. Kapologwe NA, Kalolo A, Kibusi SM, Chaula Z, Nswilla A, Teuscher T, et al.
651 Understanding the implementation of Direct Health Facility Financing and its effect on
652 health system performance in Tanzania: A non-controlled before and after mixed method
653 study protocol. Health Res Policy Syst. 2019 Jan 30;17(1).
654 13. United THE, Of R. Comprehensive Council Health Planning Guideline Fifth Edition. 2019;
655 14. Ministry of Health. Guideline for Developing Comprehensive Hospital Operational Plan (
656 CHOP ) for Regional Referral Hospitals. 2016;(August).
657 15. Mwilike B, Nalwadda G, Kagawa M, Malima K, Mselle L, Horiuchi S. Knowledge of
658 danger signs during pregnancy and subsequent healthcare seeking actions among women in
659 Urban Tanzania: a cross-sectional study. BMC Pregnancy Childbirth [Internet]. 2018 Dec
660 [cited 2024 Sep 21];18(1):4. Available from:
661 https://bmcpregnancychildbirth.biomedcentral.com/articles/10.1186/s12884-017-1628-6
662 16. Lyimo AA, Guo J, Mushy SE, Mwilike BE. Use of contraceptives and associated factors
663 among male adolescents in rural secondary schools, Coast Region, Tanzania: a school-based
664 cross-sectional study. Contracept Reprod Med [Internet]. 2024 Mar 1 [cited 2024 Sep
665 21];9(1):8. Available from:
666 https://contraceptionmedicine.biomedcentral.com/articles/10.1186/s40834-024-00268-w
667 17. Wahed T, Kaukab SST, Saha NC, Khan IA, Khanam F, Chowdhury F, et al. Knowledge of,
668 attitudes toward, and preventive practices relating to cholera and oral cholera vaccine among
669 urban high-risk groups: findings of a cross-sectional study in Dhaka, Bangladesh. BMC
670 Public Health [Internet]. 2013 Dec [cited 2024 Feb 9];13(1):242. Available from:
671 https://bmcpublichealth.biomedcentral.com/articles/10.1186/1471-2458-13-242
672 18. Subedi D. Explanatory sequential mixed method design as the third research community of
673 knowledge claim. Am J Educ Res. 2016;4(7):570–7.
674 19. Shiyanbola OO, Rao D. Using an exploratory sequential mixed methods design to adapt an
675 Illness Perception Questionnaire for African Americans with diabetes : the mixed data
676 integration process. 2021;9(1):796–817.
677 20. Surucu L, Maslakci A. Validity and Reliability in Quantitative Research. Bus Manag Stud

678	Int J. 2020;8(3):2694–726.

679 21. Jiyenze MK, Tundui C, Sirili N, Mollel H. Effects of implementing a health sector strategic
680 plan : Eidence from Tanzania. East Afr Med J. 2022;99(9):5147–55.
681 22. Lavis J. Assessing country-level efforts to link research to action. Bull World Health Organ
682 [Internet]. 2006 Aug 1 [cited 2024 Oct 4];84(8):620–8. Available from:
683 https:/[/www.ncbi.nlm.nih.gov/pmc/articles/PMC2627430/pdf/16917649.pdf/](http://www.ncbi.nlm.nih.gov/pmc/articles/PMC2627430/pdf/16917649.pdf/)
684 23. Hanney SR, Kanya L, Pokhrel S, Jones TH, Boaz A. How to strengthen a health research
685 system: WHO’s review, whose literature and who is providing leadership? Health Res Policy
686 Syst [Internet]. 2020 Dec [cited 2024 Oct 4];18(1):72. Available from: https://health-policy-
687 systems.biomedcentral.com/articles/10.1186/s12961-020-00581-1
688 24. Orton L, Lloyd-Williams F, Taylor-Robinson D, O’Flaherty M, Capewell S. The Use of
689 Research Evidence in Public Health Decision Making Processes: Systematic Review. Ross
690 JS, editor. PLoS ONE [Internet]. 2011 Jul 26 [cited 2024 Oct 4];6(7):e21704. Available
691 from: https://dx.plos.org/10.1371/journal.pone.0021704
692 25. Innvær S, Vist G, Trommald M, Oxman A. Health policy-makers’ perceptions of their use of
693 evidence: a systematic review. J Health Serv Res Policy [Internet]. 2002 Oct 1 [cited 2024
694 Oct 3];7(4):239–44. Available from: https://doi.org/10.1258/135581902320432778
695 26. Oliver K, Innvar S, Lorenc T, Woodman J, Thomas J. A systematic review of barriers to and
696 facilitators of the use of evidence by policymakers. BMC Health Serv Res [Internet]. 2014
697 Dec [cited 2024 Apr 8];14(1):2. Available from:
698 https://bmchealthservres.biomedcentral.com/articles/10.1186/1472-6963-14-2
699 27. Koon AD. A scoping review of the uses and institutionalisation of knowledge for health
700 policy in low- and middle-income countries. 2020;
701 28. Lavis JN, Paulsen EJ, Oxman AD, Moynihan R. Evidence-informed health policy 2 – Survey
702 of organizations that support the use of research evidence. Implement Sci. 2008;3(1):1–17.
703 29. Lavis JN, Wilson MG, Grimshaw JM, Haynes RB, Hanna S, Raina P, et al. Effects of an
704 evidence service on health-system policy makers’ use of research evidence: A protocol for a
705 randomised controlled trial. 2011;
706 30. Moat KA, Lavis JN. 10 best resources for . . . evidence-informed health policy making.
707 31. Gavine A. Maximising the availability and use of high-quality evidence for policymaking:
708 collaborative, targeted and efficient evidence reviews. PALGRAVE Commun. 2018;
709 32. Armstrong R, Waters E, Dobbins M, Anderson L, Moore L, Petticrew M, et al. Knowledge
710 translation strategies to improve the use of evidence in public health decision making in local
711 government: intervention design and implementation plan. Implement Sci [Internet]. 2013
712 Dec [cited 2024 Feb 16];8(1):121. Available from:
713 <http://implementationscience.biomedcentral.com/articles/10.1186/1748-5908-8-121>
714 33. Armstrong JS. Guidelines for Science: Evidence and Checklists.
715 34. Smith JD, Hasan M. Quantitative approaches for the evaluation of implementation research
716 studies. Psychiatry Res [Internet]. 2020 Jan [cited 2024 Oct 28];283:112521. Available from:
717 https://linkinghub.elsevier.com/retrieve/pii/S0165178119307024
718 35. Roberts-Lewis SF, Baxter HA, Mein G, Quirke-McFarlane S, Leggat FJ, Garner HM, et al.
719 The Use of Social Media for Dissemination of Research Evidence to Health and Social Care
720 Practitioners: Protocol for a Systematic Review. JMIR Res Protoc [Internet]. 2023 May 12
721 [cited 2024 Oct 28];12:e45684. Available from:
722 https:/[/www.researchprotocols.org/2023/1/e45684](http://www.researchprotocols.org/2023/1/e45684)
723 36. Brown G, Kyttä M, Reed P. Using community surveys with participatory mapping to
724 monitor comprehensive plan implementation. Landsc Urban Plan [Internet]. 2022 Feb [cited
725 2024 Oct 28];218:104306. Available from:
726 https://linkinghub.elsevier.com/retrieve/pii/S0169204621002693
727 37. Sörensen J, Persson AS, Olsson JA. A data management framework for strategic urban
728 planning using blue-green infrastructure. J Environ Manage [Internet]. 2021 Dec [cited 2024
729 Oct 28];299:113658. Available from:
730 https://linkinghub.elsevier.com/retrieve/pii/S0301479721017205
731 38. Lim FV, Nguyen TTH. Design-based research approach for teacher learning: a case study
732 from Singapore. ELT J [Internet]. 2022 Oct 14 [cited 2024 Oct 28];76(4):452–64. Available
733 from: https://academic.oup.com/eltj/article/76/4/452/6316630
734 39. Nabyonga-Orem J, Ssengooba F, Macq J, Criel B. Malaria treatment policy change in
735 Uganda: what role did evidence play? 2014;
736 40. Hyder F, Rothman DL. Evidence for the importance of measuring total brain activity in
737 neuroimaging.
738 41. Bennett NJ. Using perceptions as evidence to improve conservation and environmental
739 management. Conserv Biol. 2016;30(3).
740 42. Hollingworth SA. What do we need to know? Data sources to support evidence-based
741 decisions using health technology assessment in Ghana. . Epidemiology. 2020;

742

Figure	[Click here to access/download;Figure;Figure 1.docx](https://www2.cloud.editorialmanager.com/pone/download.aspx?id=38605154&guid=8a739d9e-1e49-4b8d-a191-612aa2fd5bfa&scheme=1)[<https://www2.cloud.editorialmanager.com/pone/download.aspx?id=38605154&guid=8a739d9e-1e49-4b8d-a191-612aa2fd5bfa&scheme=1>](https://www2.cloud.editorialmanager.com/pone/download.aspx?id=38605154&guid=8a739d9e-1e49-4b8d-a191-612aa2fd5bfa&scheme=1)

Figure	[Click here to access/download;Figure;Figure 2.docx](https://www2.cloud.editorialmanager.com/pone/download.aspx?id=38605155&guid=926ffc2f-9446-4d38-b865-3acdd79e94ed&scheme=1)[<https://www2.cloud.editorialmanager.com/pone/download.aspx?id=38605155&guid=926ffc2f-9446-4d38-b865-3acdd79e94ed&scheme=1>](https://www2.cloud.editorialmanager.com/pone/download.aspx?id=38605155&guid=926ffc2f-9446-4d38-b865-3acdd79e94ed&scheme=1)

Figure	[Click here to access/download;Figure;Figure 3.docx](https://www2.cloud.editorialmanager.com/pone/download.aspx?id=38605156&guid=591bd630-8ea8-4891-8541-14ffb2561819&scheme=1)[<https://www2.cloud.editorialmanager.com/pone/download.aspx?id=38605156&guid=591bd630-8ea8-4891-8541-14ffb2561819&scheme=1>](https://www2.cloud.editorialmanager.com/pone/download.aspx?id=38605156&guid=591bd630-8ea8-4891-8541-14ffb2561819&scheme=1)

Zones

9 zones

Regions

9 Regions

*Randomly selected 1 Region per Zone (In each region One Regional Referral Hospital selected)*

Districts

9 Urban Districts

*Randomly selected 1 urban district per region*

9 Rural Districts

*Randomly selected 1 rural district per region*

Facilities

2 DHs ,2HCs and 2 Dispensaries

2 DHs ,2HCs and 2 Dispensaries

Survey

Sample

N=422 randomly selected Planning team members

from the facilities.

Interview

Sample

N=52 purposefully selected interviewees (RMO, RPLO, Private

representative, DMO, DPLO, PrivateRepresentative, Facility in charges from RRHs, DHs, HCs and Dispensaries)

**Fig.3**	Sampling design

Figure	[Click here to access/download;Figure;Figure 4.docx](https://www2.cloud.editorialmanager.com/pone/download.aspx?id=38605157&guid=ee7e3496-3c8b-495a-b481-4ba68a9115da&scheme=1)[<https://www2.cloud.editorialmanager.com/pone/download.aspx?id=38605157&guid=ee7e3496-3c8b-495a-b481-4ba68a9115da&scheme=1>](https://www2.cloud.editorialmanager.com/pone/download.aspx?id=38605157&guid=ee7e3496-3c8b-495a-b481-4ba68a9115da&scheme=1)

Supporting Information

[Click here to access/download](https://www2.cloud.editorialmanager.com/pone/download.aspx?id=38605159&guid=eaf98f22-24f1-4d90-b463-679151d745cd&scheme=1)

[**Supporting Information** Supporting information.docx](https://www2.cloud.editorialmanager.com/pone/download.aspx?id=38605159&guid=eaf98f22-24f1-4d90-b463-679151d745cd&scheme=1)
