# Supplementary material for: Analysis of the determinants for using health research evidence in health planning in Tanzania: a cross-sectional study
Source: PLoS One. 2025 May 28;20(5):e0316508. doi: 10.1371/journal.pone.0316508 (PMC12118992; doi:10.1371/journal.pone.0316508)
Supplement: Attachment — Submitted filename: Response to Reviewers.docx [file pone.0316508.s005.docx]

***Analyzing the determinants for using health research evidence in health planning in Tanzania: a cross-sectional study***

**Response to reviewer’s comments**

We would like to thank the reviewer for the comments provided to improve this study. We have revised the manuscript as shown in the point-to-point responses to the reviewer’s comments provided in the table matrix below.

| **Comments** | **Authors Response** |
| --- | --- |
| **Journal requirements:** |  |
| 1. Please ensure that your manuscript meets PLOS ONE's style requirements, including those for file naming. The PLOS ONE style templates can be found at  <https://journals.plos.org/plosone/s/file?id=wjVg/PLOSOne_formatting_sample_main_body.pdf> and  <https://journals.plos.org/plosone/s/file?id=ba62/PLOSOne_formatting_sample_title_authors_affiliations.pdf> | Thank you for your comment. We have read PLOS ONE’s style requirement and have used it to make revisions including file naming for our dataset |
| 2. Please provide additional details regarding participant consent. In the ethics statement in the Methods and online submission information, please ensure that you have specified (1) whether consent was informed and (2) what type you obtained (for instance, written or verbal, and if verbal, how it was documented and witnessed). If your study included minors, state whether you obtained consent from parents or guardians. If the need for consent was waived by the ethics committee, please include this information. If you are reporting a retrospective study of medical records or archived samples, please ensure that you have discussed whether all data were fully anonymized before you accessed them and/or whether the IRB or ethics committee waived the requirement for informed consent. If patients provided informed written consent to have data from their medical records used in research, please include this information. | Thank you for the comment. We confirm that informed written consent was obtained from all participants, details have been clarified in the Ethics Statement of the manuscript. |
| 3. When completing the data availability statement of the submission form, you indicated that you will make your data available on acceptance. We strongly recommend all authors decide on a data sharing plan before acceptance, as the process can be lengthy and hold up publication timelines. Please note that, though access restrictions are acceptable now, your entire data will need to be made freely accessible if your manuscript is accepted for publication. This policy applies to all data except where public deposition would breach compliance with the protocol approved by your research ethics board. If you are unable to adhere to our open data policy, please kindly revise your statement to explain your reasoning and we will seek the editor's input on an exemption. Please be assured that, once you have provided your new statement, the assessment of your exemption will not hold up the peer review process. | Thank you for your guidance. We confirm that we will make the data fully available before acceptance ensuring compliance with our research ethics board, we have clarified this in the Data Availability Statement and are open to further guidance from the editor. |
| 4. Please amend either the abstract on the online submission form (via Edit Submission) or the abstract in the manuscript so that they are identical. | Thank you for your feedback. We have carefully reviewed both the abstract in the online submission form and the abstract in the manuscript to ensure they are identical. |
| 5. We note that Figure 2 in your submission contain [map/satellite] images which may be copyrighted. All PLOS content is published under the Creative Commons Attribution License (CC BY 4.0), which means that the manuscript, images, and Supporting Information files will be freely available online, and any third party is permitted to access, download, copy, distribute, and use these materials in any way, even commercially, with proper attribution. For these reasons, we cannot publish previously copyrighted maps or satellite images created using proprietary data, such as Google software (Google Maps, Street View, and Earth). For more information, see our copyright guidelines: <http://journals.plos.org/plosone/s/licenses-and-copyright>.  We require you to either (1) present written permission from the copyright holder to publish these figures specifically under the CC BY 4.0 license, or (2) remove the figures from your submission:  1. You may seek permission from the original copyright holder of Figure 2 to publish the content specifically under the CC BY 4.0 license.  We recommend that you contact the original copyright holder with the Content Permission Form (<http://journals.plos.org/plosone/s/file?id=7c09/content-permission-form.pdf>) and the following text:  “I request permission for the open-access journal PLOS ONE to publish XXX under the Creative Commons Attribution License (CCAL) CC BY 4.0 (<http://creativecommons.org/licenses/by/4.0/>). Please be aware that this license allows unrestricted use and distribution, even commercially, by third parties. Please reply and provide explicit written permission to publish XXX under a CC BY license and complete the attached form.”  Please upload the completed Content Permission Form or other proof of granted permissions as an "Other" file with your submission.  In the figure caption of the copyrighted figure, please include the following text: “Reprinted from [ref] under a CC BY license, with permission from [name of publisher], original copyright [original copyright year].”  2. If you are unable to obtain permission from the original copyright holder to publish these figures under the CC BY 4.0 license or if the copyright holder’s requirements are incompatible with the CC BY 4.0 license, please either i) remove the figure or ii) supply a replacement figure that complies with the CC BY 4.0 license. Please check copyright information on all replacement figures and update the figure caption with source information. If applicable, please specify in the figure caption text when a figure is similar but not identical to the original image and is therefore for illustrative purposes only.  The following resources for replacing copyrighted map figures may be helpful:  USGS National Map Viewer (public domain): <http://viewer.nationalmap.gov/viewer/>  The Gateway to Astronaut Photography of Earth (public domain): <http://eol.jsc.nasa.gov/sseop/clickmap/>  Maps at the CIA (public domain): <https://www.cia.gov/library/publications/the-world-factbook/index.html> and <https://www.cia.gov/library/publications/cia-maps-publications/index.html>  NASA Earth Observatory (public domain): <http://earthobservatory.nasa.gov/>  Landsat: <http://landsat.visibleearth.nasa.gov/>  USGS EROS (Earth Resources Observatory and Science (EROS) Center) (public domain): [http://eros.usgs.gov/#](http://eros.usgs.gov/)  Natural Earth (public domain): <http://www.naturalearthdata.com/> | Thank you for your feedback. We have reviewed Figure 2 and acknowledge the copyright requirements. To comply with the CC BY 4.0 license, we have removed the figure as required. |
| 6. Please include captions for your Supporting Information files at the end of your manuscript, and update any in-text citations to match accordingly. Please see our Supporting Information guidelines for more information: <http://journals.plos.org/plosone/s/supporting-information>. | Thank you for your feedback. We have added captions for the Supporting Information files at the end of the manuscript and updated the in-text citations accordingly. |
| **Additional Editor Comments:** |  |
| In the methodology section, you have narrated the scoring tools (likert scales and questions), but no source was cited. Please insert the citation for the source(s) in the text with their corresponding references in the reference list. | Thank you for your nice comment. We have added the appropriate citations for the scoring tools (Likert scales and questions) in the methodology section and included the corresponding references in the reference list. |
| Please read critically the comments raised by the reviewer and address each comment accordingly. |  |
| **Reviewer #1** |  |
| Specific comments: 1. Methodology-The methodology is not clear. For example, there is no specific subsection to describe the characteristics of the study participants. Also, calculation of the sample size was wrongly done by considering marginal error of 50% which does not exist. -Definition or measurement of the dependent variable which is use of health research evidence is not clear. Authors stated that four questions were used for assessing the use of health research evidence without stating the source of this approach. This affects the authenticity and also reproducibility of the results from the current study. | Thank you for your comment. We have revised the methodology section to clearly define the study participants' characteristics and corrected the sample size calculation by using an appropriate margin of error. The previously used 50% was an assumed proportion; however, there was an error in how it was presented, which we have now rectified for clarity. Additionally, we have provided a more explicit description of how the use of health research evidence was assessed, including details on the measurement of the dependent variable. To enhance the authenticity and reproducibility of our study, we have also included citations for the questions used in the assessment. |
| Results: Are not precise and lack a clear message to the readers. | Thank you for your valuable feedback. We appreciate your insights and have carefully revised the results section to enhance clarity and ensure a precise and coherent message for the readers. We have also refined our language and structure to improve readability and effectively communicate our key findings. |
| **Reviewer #2:** |  |
| I have read the manuscript and I found it good and I do not have any significant comments regarding it. Introduction is good, methodology was described very well. Results also were clear. Discussion and conclusion were good as well | Thank you so much for your thoughtful review and kind words. We truly appreciate your time and effort in reviewing our manuscript. Your constructive feedback encourages us, and we are grateful that you found the work well-structured and clear. Thank you again for your support. |
