# Supplementary material for: Analysis of the determinants for using health research evidence in health planning in Tanzania: a cross-sectional study
Source: PLoS One. 2025 May 28;20(5):e0316508. doi: 10.1371/journal.pone.0316508 (PMC12118992; doi:10.1371/journal.pone.0316508)
Supplement: Attachment — Submitted filename: Response_to_Reviewers_auresp_2.docx [file pone.0316508.s006.docx]

***Analysis of the determinants for using health research evidence in health planning in Tanzania: a cross-sectional study***

**Response to reviewer’s comments**

We would like to thank the reviewers for the comments provided to improve this study. We have revised the manuscript as shown in the point-to-point responses to the reviewer’s comments provided in the table matrix below.

| **Comments** | **Authors Response** |
| --- | --- |
| **ACADEMIC EDITOR COMMENTS** |  |
| 1. Title: Replace Analyzing with “Analysis of.” | Thank you for your comment. We have replaced “analyzing” with “Analysis of” in the title. |
| 2. Keep only the email of the corresponding author. | Thank you for your valuable feedback. We have revised the manuscript accordingly and retained only the email address of the corresponding author, as advised. |
| 3. Abstract:   - Introduction should be about the use of health research evidence and not about universal health coverage. - Your study object is to investigate the determinants of the utilization of health research evidence and not the barriers to the utilization of health research evidence. To align with your study objective, please remove all results and discussion regarding barriers to the use of health research evidence. You can produce another manuscript on the barriers associated with the utilization of health research evidence. Otherwise, rephrase your title to include the “barriers for” ……. For instance, **“Analysis of the determinants and barriers for using health research evidence in health planning in Tanzania.”** | Thank you for your insightful comments and guidance. We have revised the introduction of the abstract to focus specifically on the use of health research evidence, as recommended, and not the barriers to the utilization of the health research evidence. Additionally, we have aligned the results with the study objective by presenting all factors as determinants of utilization without framing them as barriers. We have agreed to produce another manuscript on the barriers associated with the utilization of health research evidence. |
| 4. Results:   - Delete all introductory sections of each results subheading: page 12 lines 285-289; page 16 lines 323-328; and other similar sections throughout the results section. They are not relevant to the research article and are reserved for the submission of the dissertation report. - In regression analysis, describe only the results for the adjusted model. The unadjusted model can be traced in the table. - Avoid describing the analysis parameters. Describe the intended results right away. For example: “In the adjusted regression model, the study participants from regional referral hospitals were significantly more likely to use health research evidence than those from dispensaries: (aOR = 2.67, CI: [1.89-7.92], p-value = 0.038).” - In regression analysis, use “than” instead of “compared to” (used in the chi-square test). - In all tables of results, please remove all vertical lines and retain only three horizontal lines: one under the title, one above the column heading, and one between the column headings and the body of the table. - I suggest you download and read at least three original research articles from the PLOS One Journal and learn how the results are presented. Your results are presented in the dissertation report format. | Thank you very much for your thoughtful and constructive comments. We have carefully addressed each of the suggested revisions to improve the clarity and quality of the results section. Specifically, I have removed all introductory texts under each results subheading, reported only the adjusted regression model findings, and revised the descriptions to focus directly on the key results. Additionally, we have replaced “compared to” with “than” in the regression analysis and reformatted all result tables according to the standard format by eliminating vertical lines and retaining only the recommended horizontal lines. I also appreciate the suggestion to review articles from PLOS One, which has been very helpful in aligning my work with the journal. |
| 5. Discussion:   - Your interpretation of the findings lacks an alternative explanation of the results and study strengths and limitations. Also, maintain objectivity and logical flow, and avoid repeating the results section. Please download and read at least three original research articles from the PLOS One Journal to improve your discussion and conclusion sections. | Thank you for your valuable comment. We have revised the discussion section to include alternative explanations, clearly highlight the study's strengths and limitations, and ensure objectivity and logical flow while avoiding repetition of the results. We have also reviewed relevant articles from the PLOS One Journal to enhance the quality of the discussion and conclusion. |
| 5. Conclusion   - Keep your conclusion relatively short by summarizing the most striking findings relevant to your study objective(s) | Thank you for the helpful suggestion; We have revised the conclusion to be relatively short by focusing on the most striking findings aligned with the study objectives. |
